# Regioselective approach to 5-arylsulfonylisoxazoles and their antimicrobial activity

**DOI:** 10.3762/bjoc.22.45

**Published:** 2026-04-17

**Authors:** Artem S Sazonov, Dmitry A Vasilenko, Denis V Porfiriev, Yuri K Grishin, Rimma A Gazzaeva, Alisa P Chernyshova, Maxim A Kryakvin, Anna A Baranova, Vera A Alferova, Elena B Averina

**Affiliations:** 1 Department of Chemistry, Lomonosov Moscow State University, Leninskie Gory, 1-3, Moscow 119991, Russian Federation; 2 North-Ossetian State University, Vatutina st. 46, Vladikavkaz, 362025 Russian Federation; 3 Center for Molecular and Cellular Biology, Moscow, 121205, Russian Federation; 4 Shemyakin-Ovchinnikov Institute of Bioorganic Chemistry, Miklukho-Maklaya 16/10, 117997, Moscow, Russian Federation; 5 School of Bioengineering and Bioinformatics, Lomonosov Moscow State University, 119991 Moscow, Russian Federation

**Keywords:** antimicrobial activity, aromatic nucleophilic substitution, isoxazoles, oxidation, sulfonylisoxazoles

## Abstract

This study presents a novel regioselective synthesis of 3-electron-withdrawing-group-(EWG)-substituted 5-sulfonyl- and 5-sulfinylisoxazoles from 3-EWG-5-nitroisoxazoles via nucleophilic aromatic substitution with thiophenols followed by oxidation with *m*-chloroperbenzoic acid (mCPBA). The scope of the reactions was explored, demonstrating high yields across a variety of functional groups and substituents. Optimized conditions enabled selective oxidation of thioaryl groups to sulfonyl or sulfinyl derivatives. Biological evaluation revealed that several compounds, especially 3-nitro-5-sulfonyl- and 5-sulfinylisoxazoles, exhibit potent antimicrobial activity against Gram-positive bacteria, fungi, and notably low MICs comparable to those of standard drugs. The mechanism of action studies indicate that these compounds induce the bacterial SOS response without inhibiting DNA synthesis-related enzymes such as DNA polymerase I, DNA gyrase, or topoisomerases I and IV, suggesting activation via bacterial reductases. These findings highlight the potential of these isoxazole derivatives as promising antimicrobial agents and provide new insights into their mechanism of action.

## Introduction

**Figure 1:**
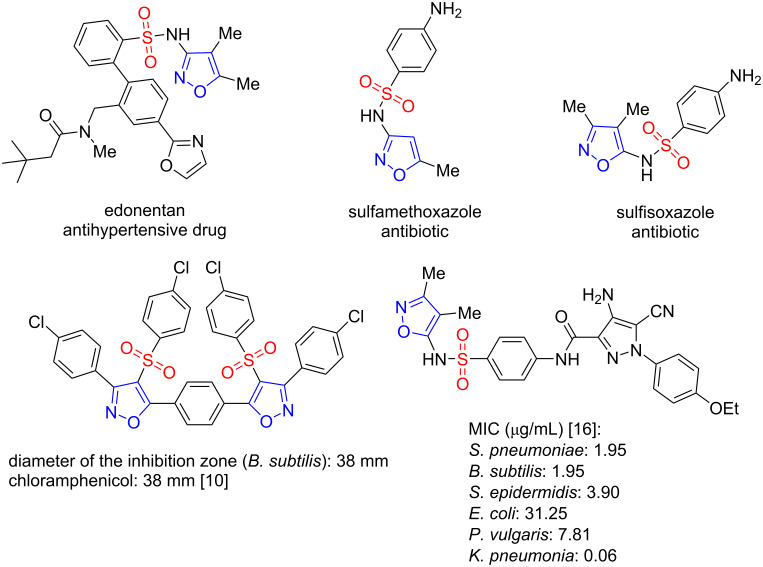
Examples of sulfonylisoxazoles with biological activities.

The isoxazole ring represents an important building block for the synthesis of promising compounds with a wide spectrum of biological activity [1–5]. The attractiveness of isoxazole derivatives for medicinal chemistry is determined by high-affinity binding to many targets, low toxicity of isoxazole-containing compounds and the use of the isoxazole ring as a bioisoster of the pyridine ring and carboxyl group [6–9]. On the other hand, the sulfonamide and sulfone groups are commonly found in the design of potential therapeutics with various types of biological activities, such as antimicrobial, antitubercular, antileishmanial, antiviral, anticancer, antidiabetic, anti-inflammatory, for the treatment of Alzheimer diseases, etc. [10–16]. Among the sulfonyl-containing heterocycles with confirmed efficacy against microorganisms (bacteria and fungi), sulfonylisoxazoles have attracted considerable attention as potential antibacterial drugs, with the isoxazole core in these compounds being essential for the activity [10]. Representative examples of biological active sulfonylisoxazoles, including marked drugs (see, for example, edonentan, sulfamethoxazole and sulfisoxazole) and compounds with the most promising activity are shown in Figure 1. Also, modern strategies to combat the antibiotic resistance include, in particular, the synthesis of novel antibacterial agents based on isoxazole derivatives [17]. In this regards, the development of efficient and convenient methods for the sulfonyl-containing isoxazoles synthesis has received great attention.

**Scheme 1:**
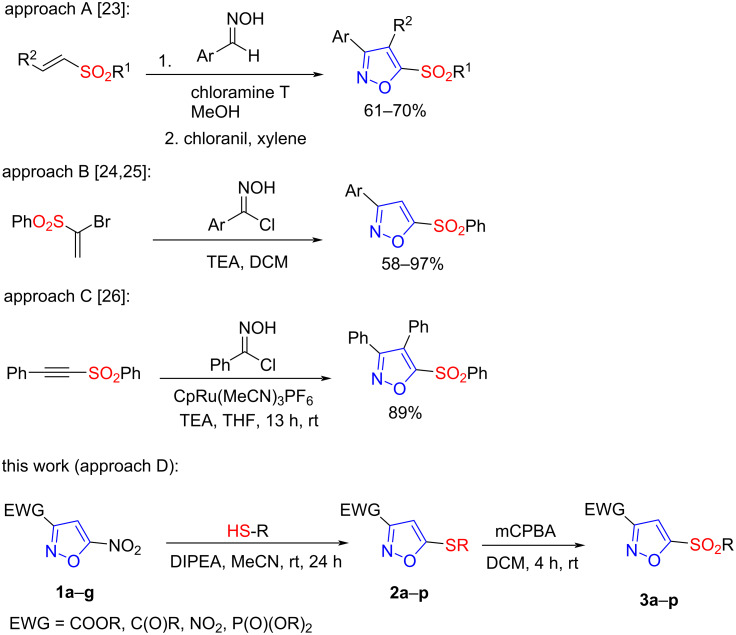
Reactions for preparing 5-sulfonylisoxazoles.

The most common method for the synthesis of substituted isoxazoles is based on the 1,3-dipolar cycloaddition of nitrile oxide with alkynes or alkenes followed by oxidation of the resulting isoxazoline product in the latter case [18–22]. This approach, in three variations, is also applicable to obtain 5-sulfonylisoxazoles (Scheme 1, approaches A, B, C). As shown in Scheme 1, nitrile oxides are generated in situ by oxidation of aldoximes with chloramine T (approach A) or by dehydrohalogenation of the corresponding oxime halide under basic conditions (approaches B and C). Subsequently, the nitrile oxide reacts with 1,2-disubstituted alkenes containing a sulfonyl group to form 4,5-dihydroisoxazoles, which are then oxidized with chloranil to provide isoxazole [23] (approach A). Another synthetic protocol involves the 1,3-dipolar cycloaddition of nitrile oxide to 1-bromo-1-EWG-alkenes, resulting in 4-unsubstituted isoxazoles with various aryl substituents in position 3 [24–25] (approach B). One example describes the Ru-catalyzed reaction of nitrile oxide with alkynyl sulfone providing 5-sulfonylisoxazole with high regioselectivity [26] (approach C).

Despite the widespread application of nitrile oxides in cycloaddition reactions, complete control of regioselectivity without metal catalysis is not always achievable. Another problem with this method is the tendency of nitrile oxides to form dimers (furoxanes), which entails a decrease in the yield of isoxazoles. Due to dimerization, nitrile oxides are generally limited to aryl-substituted derivatives, making the abovementioned approaches inapplicable for the synthesis of 5-sulfoisoxazoles with an additional functional group. To circumvent these limitations, we proposed a novel chemo- and regioselective synthetic approach to 5-sulfonylisoxazoles based on nucleophilic aromatic substitution of the nitro group in 3-EWG-5-nitroisoxazoles by treatment with thiophenols as S-nucleophiles and subsequent oxidation of the thioaryl group (Scheme 1, approach D). Previously, we reported that 3-EWG-5-nitroisoxazoles readily react with various *N,O,S*-nucleophiles affording nucleophilic substitution products of the 5-nitro group in high yields [27] and we also demonstrated that this approach is a powerful tool to functionalize the isoxazole ring [28–29]. However, the synthesis of sulfones **3** is problematic using the nucleophilic aromatic substitution reaction of the nitro group of 5-nitroisoxazoles **1**. Thus, the two-step approach presented in this work is the most reasonable and effective for the synthesis of 5-sulfonylisoxazoles.

## Results and Discussion

### Chemistry

**Scheme 2:**
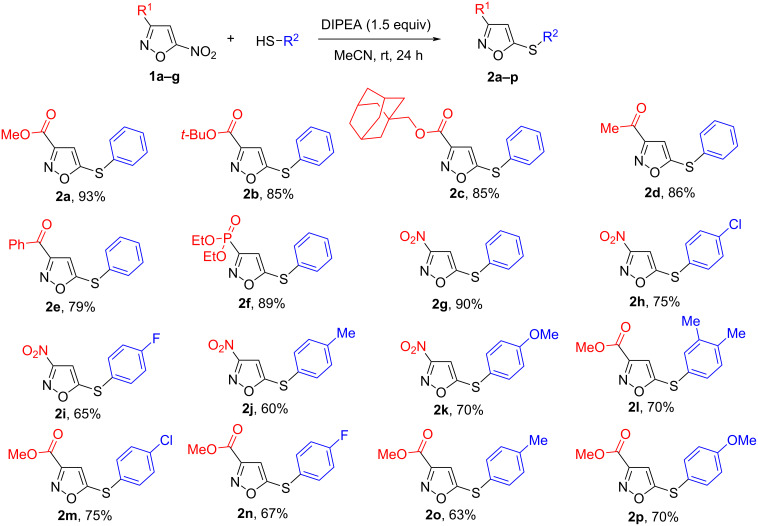
Scope of 5-nitroisoxazoles **1a**–**g** in the reaction with thiophenols.

Initially, a series of 3-EWG-5-thioarylisoxazoles **2a**–**p** were synthesized according to our previously reported procedure [27] from readily available 3-EWG-5-nitroisoxazoles [30] and thiophenols in the presence of DIPEA as a base (Scheme 2). It was found that 5-nitroisoxazoles bearing diverse functional groups react successfully with thiophenol, and a scope of *S*-nucleophiles was also explored in the S_N_Ar reactions with 3-nitro- and 3-methoxycarbonyl-substituted 5-nitroisoxazoles **1a** and **1g**. The obtained results revealed that the nature of the substituents in the isoxazole cycle as well as in the aromatic ring of thiophenols does not have a noticeable influence on the reaction yields. The S_N_Ar reactions of thiophenols with electron-withdrawing (F, Cl) or electron-donating groups (Me, OMe) at position 4 of the aromatic ring proceed smoothly, affording 3-EWG-5-thioisoxazoles **2a–p** in 60–93% yield. The slight decrease in the yield of products **2h–p** can be explained by their close chromatographic mobility with the starting thiophenols, which should be used in excess (1.5 equiv) for complete conversion in S_N_Ar reactions (Scheme 2).

To find the optimal conditions for the oxidation reaction, **2a** as a model substrate was treated with different equivalents of mCPBA under varying temperatures and reaction times (Table 1). It was found that the reaction proceeds completely to give the exhaustive oxidation product **3a**, when **2a** was treated with 2.5 equivalents of mCPBA at room temperature. Thus, we used these conditions as the standard reaction conditions for our further studies. Additionally, it was found that monooxidation product **4a** could also be obtained in the reaction by reducing the number of equivalents of mCPBA. Thus, using 1.0 or 1.2 equivalents of mCPBA provided a mixture with the monooxidation product **4a** as the major product in 74% yield. Unfortunately, changing the reaction time or temperature did not have any effect on the result.

**Table 1:** Optimization of oxidizing conditions.

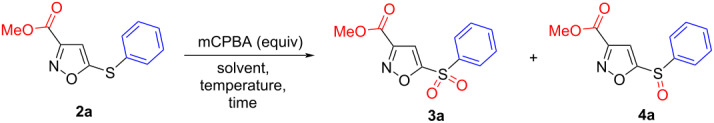							

No.	Solvent	Temperature (°C)	Time (h)	mCPBA (equiv)	Conversion^a^ (yield) (%)		

					**2a**	**3a**	**4a**

**1**	DCM	20	24	0.8	34	0	66
**2**	DCM	20	24	1.0	8	10	82
**3**	DCE	60	2	1.0	12	7	81
**4**	**DCM**	**20**	**24**	**1.2**	**8**	**9**	**83 (74)**
**5**	DCM	20	24	1.5	0	41	59
**6**	DCE	60	2	2.5	0	100	0
**7**	**DCE**	**60**	**0.5**	**2.5**	**0**	**100 (91)**	**0**
**8**	DCM	20	24	2.5	0	100	0
**9**	DCM	20	2	2.5	0	96	4
**10**	**DCM**	**20**	**4**	**2.5**	**0**	**100 (93)**	**0**

^a^The conversion was obtained from ^1^H NMR spectra of the reaction mixture.

The rate of oxidation of 5-thioisoxazoles **2** to sulfoxides **4**, and then of sulfoxides **4** to sulfones **3,** can vary significantly [31]. To verify this hypothesis, the progress of the reaction of 5-thioisoxazoles **2a** with mCPBA (2.5 equiv) in CDCl_3_ was monitored using ^1^H NMR (Figure S1 in Supporting Information File 1). The results revealed that the oxidation of isoxazole **2a** to sulfoxide **4a** proceeds quickly, and complete conversion of isoxazole **2a** is achieved within 5 min. In contrast, the subsequent reaction of **4a** to **3a** proceeds is much slower, and only after 4 hours does the C(4)–H signal of compound **4a** at δ 7.05 ppm in the ^1^H NMR spectra finally disappear.

**Scheme 3:**
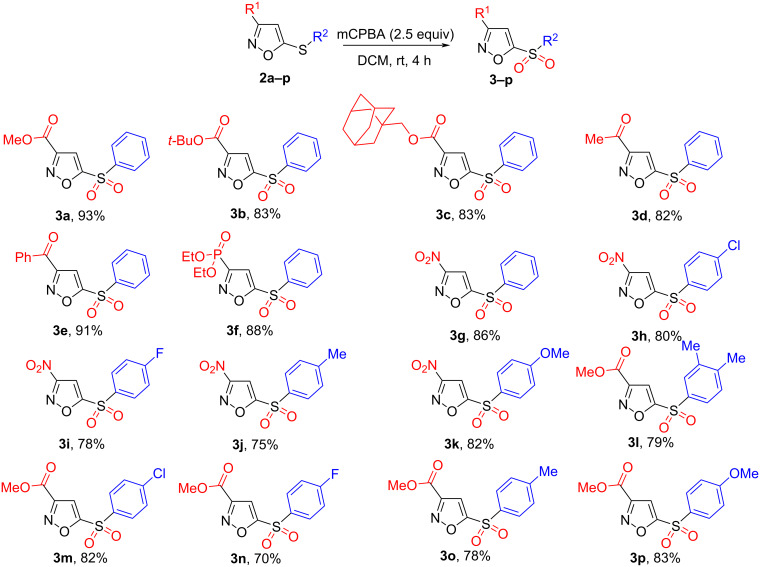
Scope of 5-thioisoxazoles **2a-p** in the reaction with mCPBA.

With optimized conditions in hand, we turned our attention to examine the substrate scope. As shown in Scheme 3, isoxazole derivatives bearing different electron-withdrawing groups in position 3 such as carboxyl, acetyl, phosphonate, or nitro groups were well tolerated under oxidation conditions (**3a**–**g**). Also, isoxazoles bearing either electron-donating or -withdrawing substituents at the phenyl rings of the thioaryl moieties reacted smoothly with mCPBA under identical conditions, affording the corresponding isoxazoles **3h**–**p** in excellent yields.

**Scheme 4:**
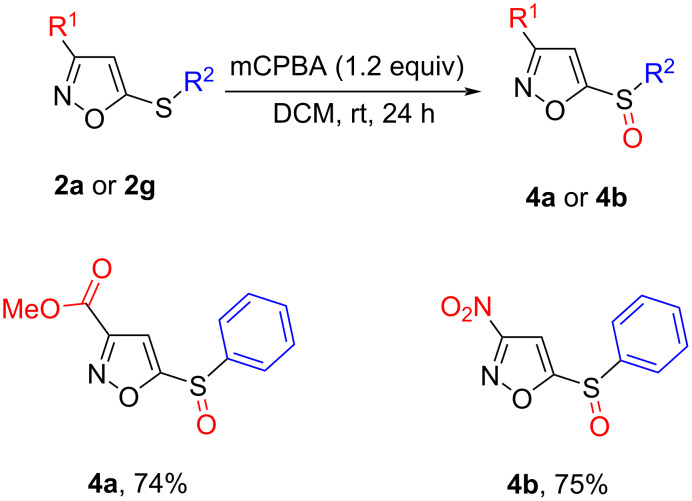
Oxidation of 5-thioisoxazoles into 5-sulfinylisoxazoles.

Also, when 3-nitro-5-thiophenylisoxazole (**2g**) and methyl 5-thiophenylisoxazole carboxylate **2a** reacted with 1.2 equiv mCPBA, monooxydated derivatives **4a** and **4b** were obtained (Scheme 4).

### Biology

In the second part of the study the screening of antibacterial and antifungal activities was performed by disc diffusion assays for a series of 5-sulfonylisoxazoles **3a–p**, as well as some of their synthetic precursors, 5-thioisoxazoles **2j**, **2k**, **2n**, **2l**, and 5-sulfinylisoxazoles **4a** and **4b**. A panel of Gram-positive bacteria strains (*Bacillus subtilis* ATCC 6633, *Enterococcus faecalis* ATCC 29212, *Staphylococcus aureus* ATCC 29213), Gram-negative bacteria strain (*Escherichia coli* ATCC 25922), and two fungi strains (*Aspergillus niger* INA 00760 and *Candida albicans* CBS 8837*)* were selected for testing. For the active compounds minimal inhibitory concentrations (MIC is in μg/mL) were determined, employing amphotericin B, vancomycin, ampicillin and clotrimazole as reference standards (Table 2). It was found, that most active isoxazole derivatives contain a nitro group in position 3 of the isoxazole cycle. Among 5-sulfonylisoxazoles, 3-nitro-substituted heterocycles **3g–k** exhibited potent antimicrobial activity with MICs ranging from 0.5 to 16 μg/mL against all tested microbial strains except *E. coli* (Table 2). The most active 3-nitroisoxazoles **3h**, **3i**, **3j** bearing 4-chloro, 4-fluorophenyl or 4-tolyl groups in position 5 of the isoxazole ring demonstrated the highest efficacy against *C. albicans* (MIC; 0.5–1 μg/mL) and *S. aureus* (MIC; 1–2 μg/mL). As can be seen from Table 2, the MICs of compounds **3h**, **3i**, **3k** for the fungal species *C. albicans* are notably lower compared to the well-known antifungal drug clotrimazole and are similar to the MIC of vancomycin for Gram-positive bacteria *S. aureus*. In the pair of 5-sulfinylisoxazoles **4a** and **4b**, the 3-nitro derivative **4b** turned out to be comparable or even more active against the studied strains, including *E. coli*, compared to the corresponding sulfonyl-substituted analog **3g**, whereas 5-sulfinylisoxazole **4a** bearing a methoxycarbonyl group conferred negligible activity (Table 2).

**Table 2:** MIC (μg/mL) against bacterial and fungal lines.

Compound	MIC (μg/mL)					

	*A. niger* INA 00760	*C. albicans* CBS 8837	*B. subtilis* ATCC 6633	*St. aureus* ATCC 29213	*Ent. faecalis* ATCC 29212	*E. coli* ATCC 25922

**3g**	16–32	4–8	2–4	2	**–**	128–256
**3h**	**2**	**0.5**	**2–4**	**1–2**	**4–8**	**–**
**3i**	**8**	**1–2**	**2**	**2–4**	**–**	**–**
**3j**	**4–8**	**0.5**	**2–4**	**1**	**8–16**	–
**3k**	8	2–4	4–8	4	16–32	**–**
**3m**	64	–	–	64	–	–
**3n**	128	–	>128	–	–	–
**3l**	>128	–	>128	–	–	–
**4a**	64–128	32–64	32	>128	**–**	>256
**4b**	4	8	1	1	**–**	32–64
amphotericin B	0.06–0.12	–	–	–	–	–
vancomycin	–	–	0.25	1	4	–
ampicillin	–	–	–	–	–	2
clotrimazole	–	4	–	–	–	–

Designation "–" means "not determined".

To evaluate the possible mechanism of antibiotic action on bacterial cells, we have studied the antibacterial activity of the isoxazole derivatives on the reporter strains *E. coli ΔtolC pDualrep2 (AmpR)* and *E. coli lptD^mut^ pDualrep2.1 (KanR)* as described previously [32–33]. Briefly, these reporter strains express different fluorescent proteins depending on the mechanism of action of the antibiotic. In case of activation of the antibiotic-induced SOS-response, the *rfp* gene was expressed in the cell of the reporter strains, and disruption of the translation mechanism led to the expression of the *katushka2S* gene. When scanning, the signal from the RFP protein was displayed in green pseudocolor, and from Katushka2S in red.

**Figure 2:**
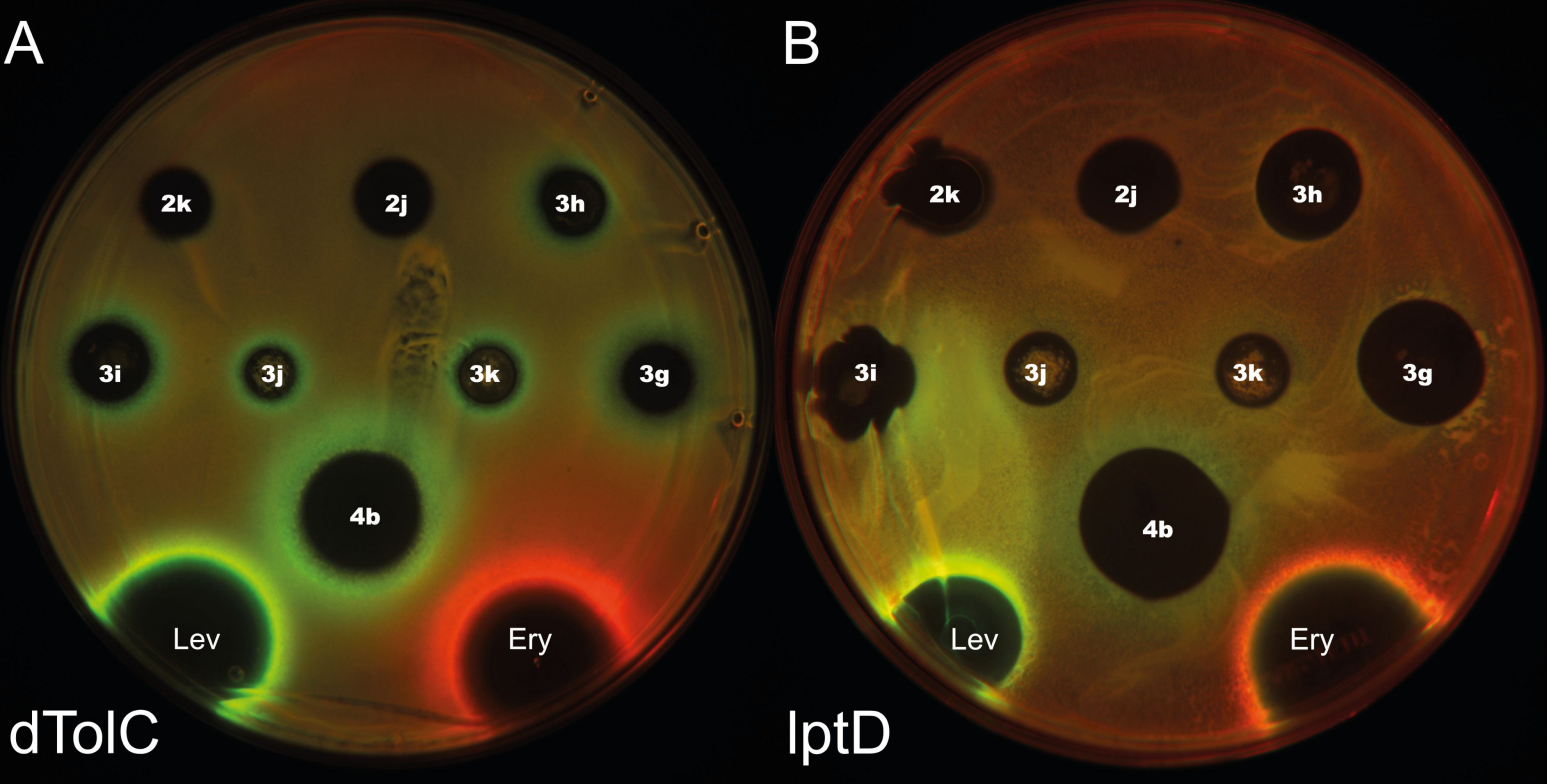
The samples of isoxazole derivatives trigger SOS response in *E. coli* reporter strains. A) Agar plate coated with *E. coli* Δ*tolC* pDualrep2 (AmpR), B) agar plate coated with *E. coli lptD^mut^* pDualrep2.1 (KanR). Both plates spotted with isoxazole samples (3 μL of 100 mM DMSO solutions) along with erythromycin (Ery, 5 mg/mL) and levofloxacin (Lev, 25 mg/mL). Plates were scanned in Cy3 (for TurboRFP) and Cy5 (for Katushka2S) channels, shown as green and red pseudocolor, respectively.

Most of the studied compounds demonstrated low antibacterial activity against the reporter strains, however, 3-nitrosulfonylisoxazoles **3h**, **3g** and monooxidated 3-nitrosulfonylisoxazole **4b** exhibited high activity against both reporter strains. Furthermore, these samples demonstrated strong induction of the RFP reporter protein, indicating that their antibacterial action is associated with induction of the SOS response in bacterial cells (Figure 2).

The SOS response is a global response to DNA damage which can occur due to several reasons, including, but not limited to, inhibition of enzymes such as DNA gyrase, DNA polymerase or topoisomerases I and IV. We conducted studies of inhibitory activity of samples **3h**, **3g** and **4b** against the above-mentioned enzymes as described previously [34].

**Figure 3:**
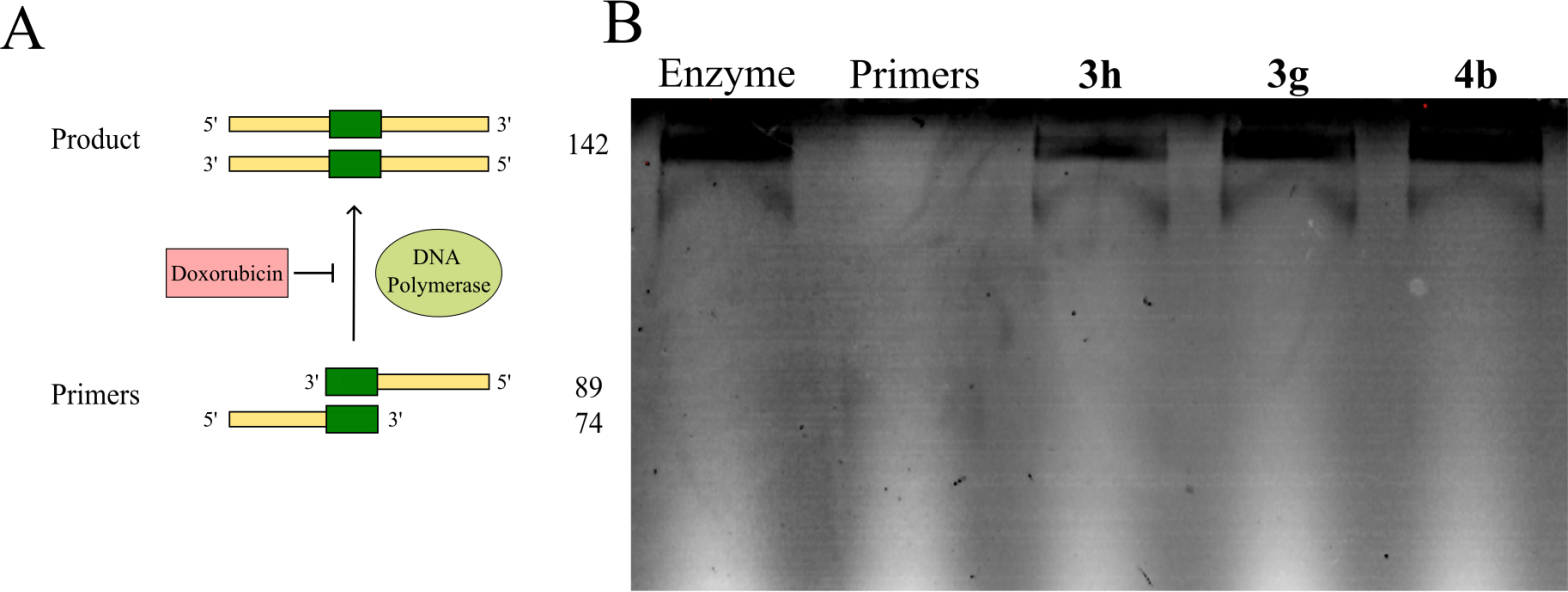
Inhibition of Klenow fragment of *E. coli* DNA polymerase I. A) The principal scheme of the Klenow fragment test. If DNA polymerase I is inhibited by antibiotic doxorubicin, the synthesis of the full-length product will be compromised, and a complete product will not be generated. B) The tested compounds (**3h**, **3g**, and **4b**) were incubated with primers, dNTPs, and DNA polymerase I, and subsequently analyzed by 10% urea-PAGE gel electrophoresis. No differences were observed compared to the enzyme-only lane, indicating that none of the compounds inhibited DNA polymerase I activity.

Firstly, we conducted the Klenow fragment test to assess potential inhibitory activity on DNA polymerase I. The principal scheme of this test is shown in Figure 3A. Briefly, two primers (74 bases and 89 bases) anneal to each other, and the Klenow fragment of DNA polymerase I extend them to form a complete product of 142 bases. If the DNA polymerase is inhibited by an antibiotic, the synthesis of the full-length product will be impaired, and the complete product will not be generated. Subsequently, inhibition of DNA polymerase can disrupt DNA replication, leading to stalled replication forks and impaired DNA synthesis. This disruption triggers cell cycle arrest, which can ultimately result in apoptosis and cell death. However, all tested samples **3h**, **3g** and **4b** exhibited no significant difference compared to the control lane (Figure 3B), indicating that they did not inhibit DNA polymerase I activity.

**Figure 4:**
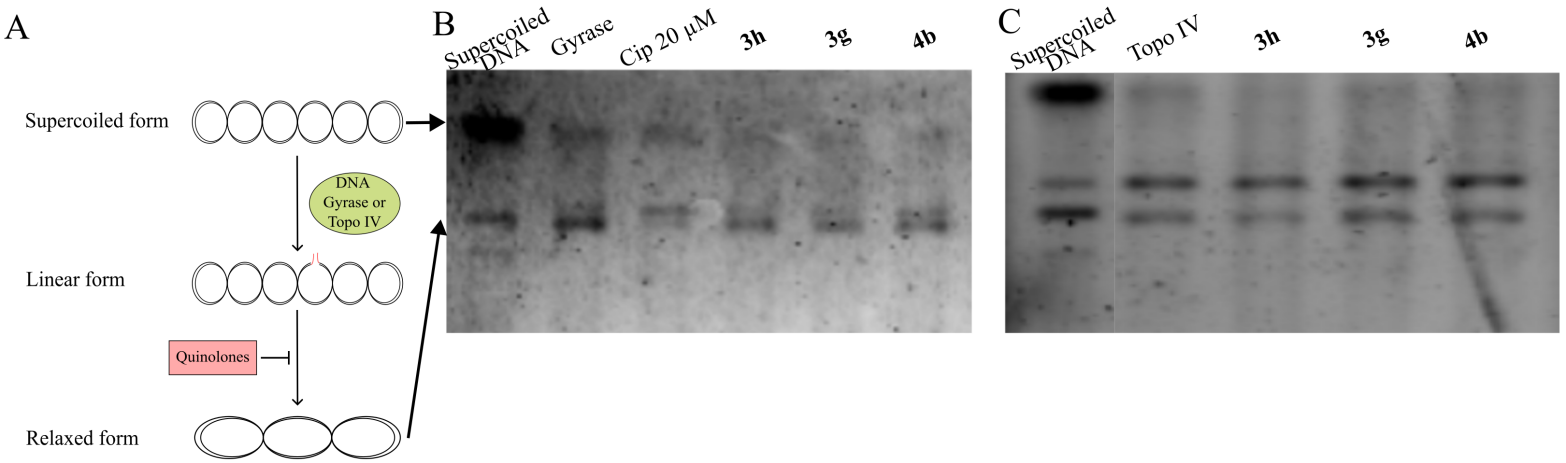
Inhibition of *E. coli* DNA gyrase and Topo IV cleavage activity. A) The principal scheme of *E. coli* DNA gyrase and Topo IV cleavage activity, which may be inhibited by quinolones. B) The tested compounds (**3h**, **3g**, and **4b**) along with control antibiotic Cip were incubated with supercoiled DNA and enzymes, and subsequently analyzed by 1% agarose gel electrophoresis. No differences were observed compared to the enzyme-only lanes, indicating that none of the compounds inhibited DNA gyrase or DNA Topo IV activity (C).

Next, we examined the potential inhibition of DNA cleavage mediated by DNA gyrase or DNA topoisomerase IV (Topo IV). In this test (Figure 4A), supercoiled DNA is present within the system. Topo IV catalyzes an ATP-dependent cleavage of both DNA strands, followed by the passage of the strands through the break and subsequent ligation, leading to DNA relaxation. DNA gyrase performs a similar function but without the requirement for ATP hydrolysis. The antibiotic ciprofloxacin (Cip), which inhibits DNA gyrase, was used as the positive control. In this test, all tested isoxazoles **3h**, **3g** and **4b** exhibited no significant difference compared to the control lane with enzyme (Gyrase or Topo IV) added (Figure 4B and 4C), indicating that they did not inhibit DNA gyrase or Topo IV activity.

**Figure 5:**
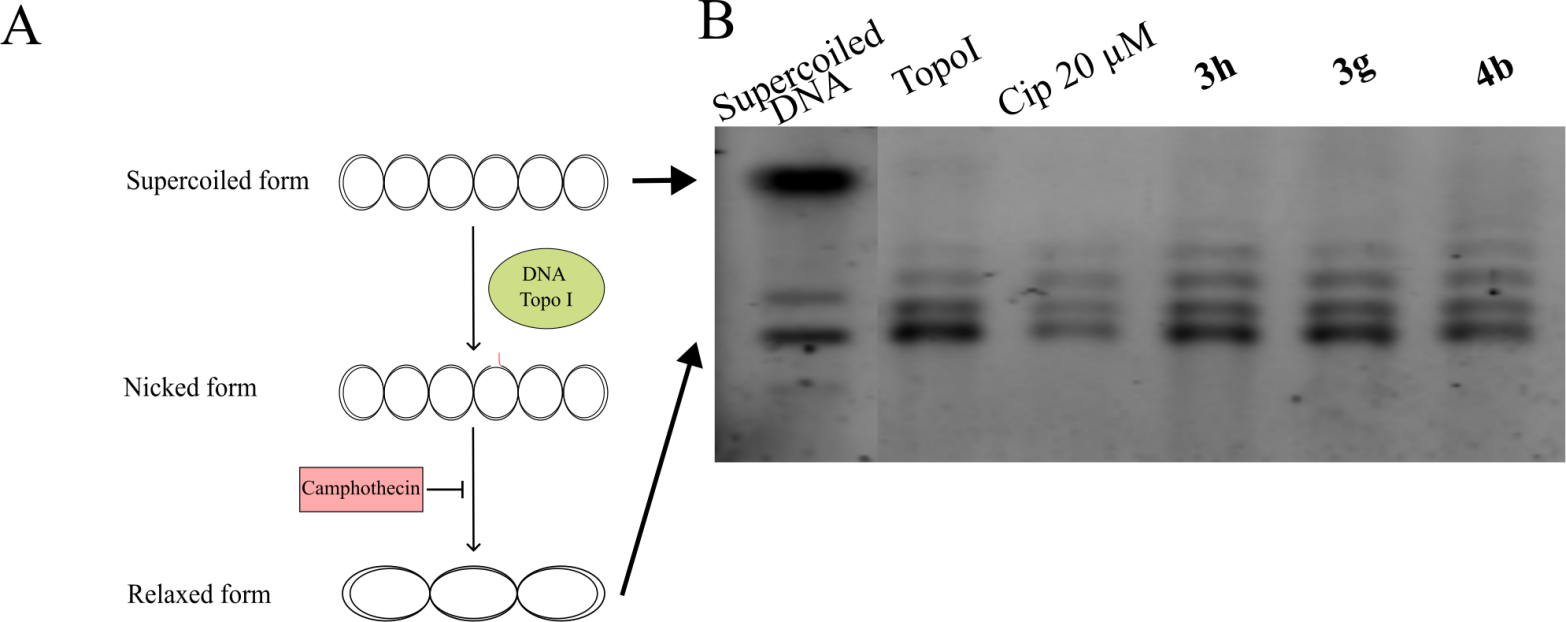
Inhibition of *E. coli* DNA Topo I cleavage activity. A) The principal scheme of *E. coli* DNA Topo I cleavage activity, which may be inhibited by campothecin. B) The tested compounds (**3h**, **3g**, and **4b**) were incubated with supercoiled DNA and enzyme, and subsequently analyzed by 1% agarose gel electrophoresis. No differences were observed compared to the enzyme-only lane, indicating that none of the compounds inhibited the DNA Topo I activity.

Further, we tested the possibility of similar DNA cleavage mediated by DNA topoisomerase I (Topo I). Unlike previously discussed DNA gyrase and Topo IV, which perform double-strand breaks and strand passage, Topo I catalyzes the introduction of a transient single-strand break in the DNA. This process occurs without the requirement for ATP hydrolysis, allowing for the relaxation of supercoiled DNA by alleviating torsional strain through the controlled cleavage and rejoining of one DNA strand (Figure 5A). Again, all tested samples **3h**, **3g** and **4b** exhibited no significant difference compared with the control lane corresponding to enzyme without antibiotic (labeled as Topo I) (Figure 5B), indicating that they did not inhibit Topo I activity.

**Figure 6:**
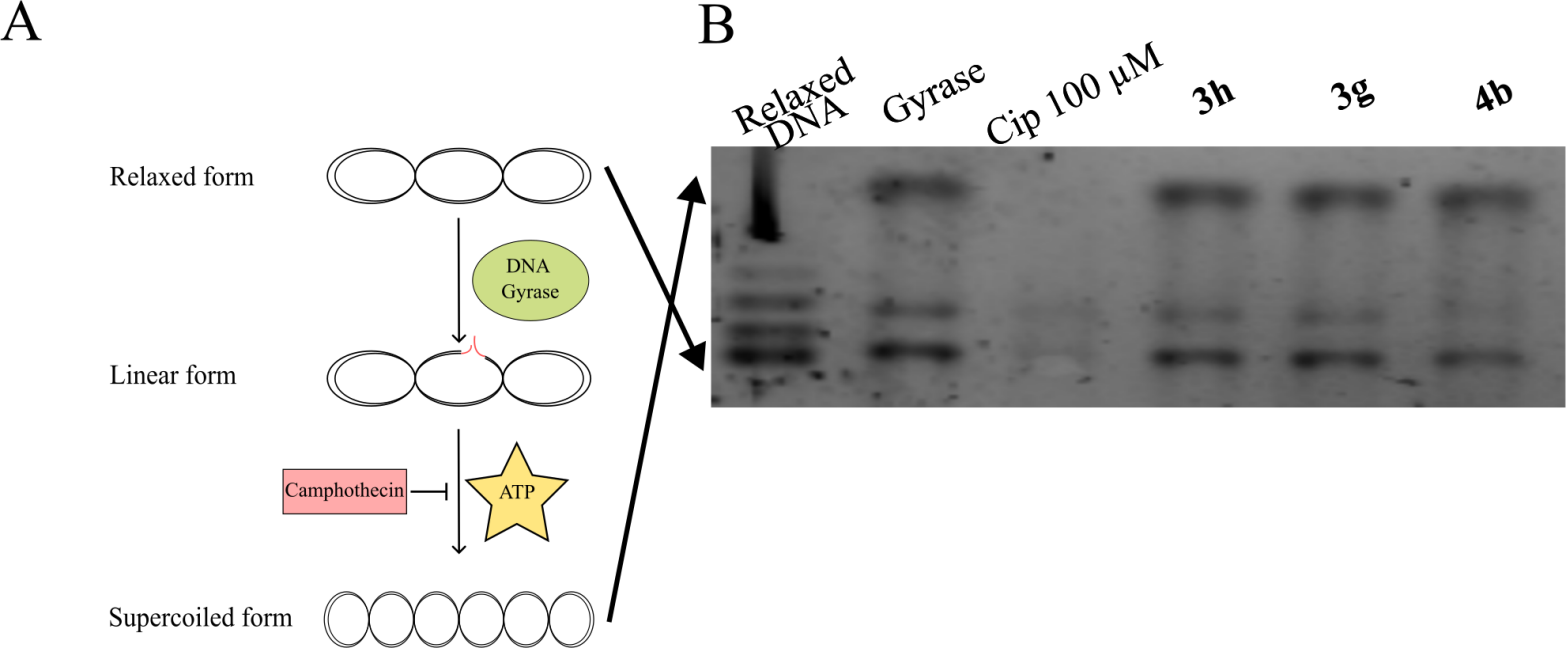
Inhibition of *E. coli* DNA gyrase supercoiling activity. A) The principal scheme of *E. coli* DNA gyrase supercoiling activity, which may be inhibited by ciprofloxacin. B) The tested compounds (**3h**, **3g**, and **4b**) were incubated with relaxed DNA and enzyme, and subsequently analyzed by 1% agarose gel electrophoresis. No differences were observed compared to the enzyme-only lane, indicating that none of the compounds inhibited the DNA gyrase activity.

The opposite process, supercoiling, is mediated by DNA gyrase, which introduces negative supercoils into DNA through an ATP-dependent mechanism involving double-strand cleavage, strand passage, and ligation (Figure 6A). All tested samples **3h**, **3g** and **4b** exhibited no significant difference compared to the control lane corresponding to enzyme without antibiotic (labeled as gyrase) (Figure 6B), indicating that they did not inhibit the DNA gyrase supercoiling activity.

**Figure 7:**
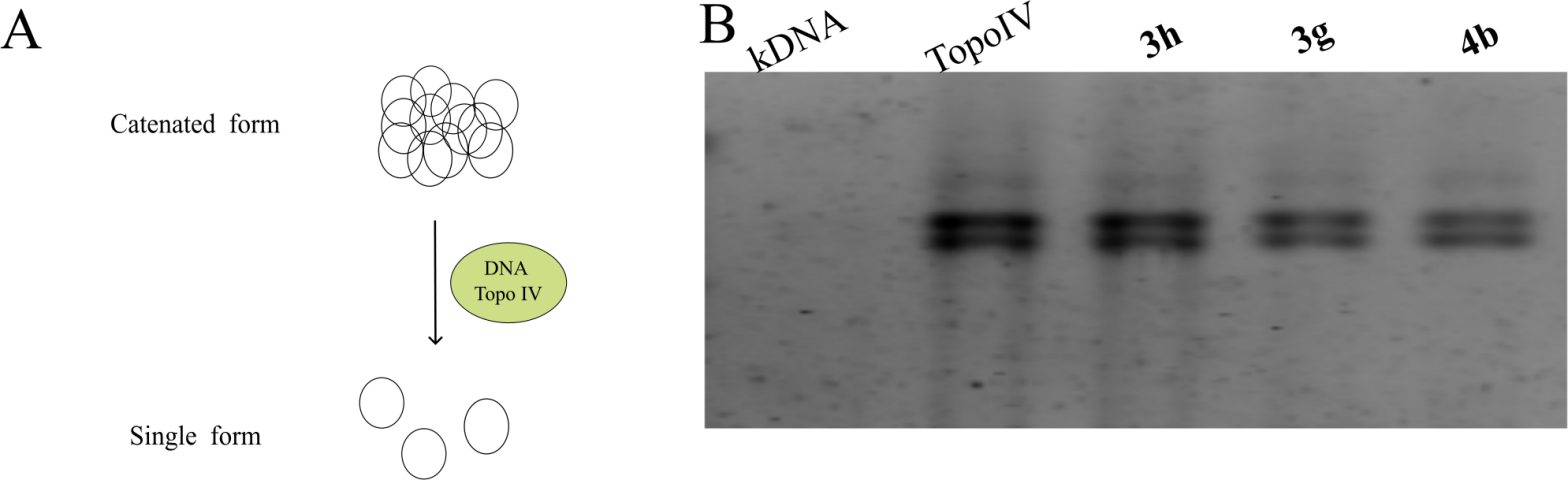
Inhibition of *E. coli* DNA Topo IV decatenation activity. A) The principal scheme of of *E. coli* DNA Topo IV decatenation, which may be inhibited by quinolones. B) The tested compounds (**3h**, **3g**, and **4b**) were incubated with kDNA and enzyme, and subsequently analyzed by 1% agarose gel electrophoresis. No differences were observed compared to the enzyme-only lane, indicating that none of the compounds inhibited DNA Topo IV decatenation activity.

Finally, we studied the inhibition of DNA Topo IV decatenation activity. This process of untangling or separating interlinked DNA molecules (Figure 7A) is an essential step in DNA replication and cell division. In bacteria, DNA Topo IV plays a crucial role in decatenating DNA molecules that have become interlinked during replication. Still, all tested compounds **3h**, **3g** and **4b** exhibited no significant difference compared to the control lane (Figure 7B), indicating that they did not inhibit DNA Topo IV decatenation activity.

## Conclusion

In conclusion, we have developed a novel regioselective approach to the synthesis of 3-EWG-5-sulfonylisoxazoles or 3-EWG-5-sulfinylisoxazoles from readily available 3-EWG-5-nitroisoxazoles. The method permits to obtain isoxazole derivatives with a variety of functional groups in the isoxazole cycle and aryl substituents in the sulfonyl fragment. The screening of the obtained compounds for antibacterial and antifungal activities revealed highly potent compounds with a broad spectrum of activity. A series of 3-nitroisoxazole, especially 5-sulfonyl derivatives **3h**, **3i** and **3j** demonstrated significant activity against bacterial and fungal strains comparable to standard drugs. Preliminary studies did not allow us to draw a decisive conclusion about the mechanism of action of the active compounds, but these will be the subject of our further research.

## Supporting Information

File 1 General synthetic and biological procedures, characterization data and copies of ^1^H, ^13^C{^1^H}, ^19^F, ^31^P, ^1^H-^13^C HSQC, ^1^H-^13^C HMBC NMR spectra, HRMS spectra and the results of the elemental analysis of all synthesized compounds.

## Data Availability

All data that supports the findings of this study is available in the published article and/or the supporting information of this article
