# Supplementary material for: Regioselective approach to 5-arylsulfonylisoxazoles and their antimicrobial activity
Source: Beilstein J Org Chem. 2026 Apr 17;22:592–602. doi: 10.3762/bjoc.22.45 (PMC13159296; doi:10.3762/bjoc.22.45)

BEILSTEIN JOURNAL OF ORGANIC CHEMISTRY

Supporting Information

for

Regioselective approach to 5-arylsulfonylisoxazoles and their antimicrobial activity

Artem S. Sazonov, Dmitry A. Vasilenko, Denis V. Porfiriev, Yuri K. Grishin, Rimma A. Gazzaeva, Alisa P. Chernyshova, Maxim A. Kryakvin, Anna A. Baranova, Vera A. Alferova and Elena B. Averina

Beilstein J. Org. Chem. **2026,** *22,* 592–602. doi:10.3762/bjoc.22.45

General synthetic and biological procedures, characterization data and copies of ^{1}H, $^{13}C\{^{1}H\}$, ^{19}F, ^{31}P, ^{1}H-^{13}C HSQC, ^{1}H-^{13}C HMBC NMR spectra, HRMS spectra and the results of the elemental analysis of all synthesized compounds

License and Terms: This is a supporting information file under the terms of the Creative Commons Attribution License (https://creativecommons.org/licenses/by/4.0). Please note that the reuse, redistribution and reproduction in particular requires that the author(s) and source are credited and that individual graphics may be subject to special legal provisions.
The license is subject to the *Beilstein Journal of Organic Chemistry* terms and conditions: (https://www.beilstein-journals.org/bjoc/terms)

Chemistry

NMR spectra were recorded on spectrometers Bruker Avance 400 and Agilent 400-MR (400.0 MHz for ^{1}H; 100.6 MHz for ^{13}C, 162.0 MHz for ^{31}P, 376.3 MHz for ^{19}F) at rt; the chemical shifts δ were measured in ppm with respect to the $CDCl_3$ (^{1}H: δ = 7.26 ppm, ^{13}C: δ = 77.16 ppm), H_3PO_4 as external standard for ^{31}P and $CFCl_3$ as external standard for ^{19}F. Chemical shifts (δ) are given in ppm; *J* values are given in Hz. When necessary, assignments of signals in NMR spectra were made using 2D techniques. Accurate mass measurements (HRMS) were performed on a Bruker micrOTOF II instrument using electrospray ionization (ESI). The measurements were done in a positive ion mode (interface capillary voltage 4500 V). Melting points (mp) are uncorrected. Thin layer chromatographic method (TLC) was conducted on DC-Fertigfolien ALUGRAM pre-coated silica gel 60-F254 plates; the detection was done by an UV lamp (254 and 365 nm) and chemical staining (5% aqueous solution of $KMnO_4$). Column chromatography was performed on silica gel (230–400 mesh, Merck).

5-Nitroisoxazoles **1a**–**g** were synthesized by described methods [1].

All other starting materials were commercially available.

All reagents except commercial products of satisfactory quality were purified by literature procedures prior to use.

NMR study of the reaction of 5-thioisoxazole 2a with mCPBA

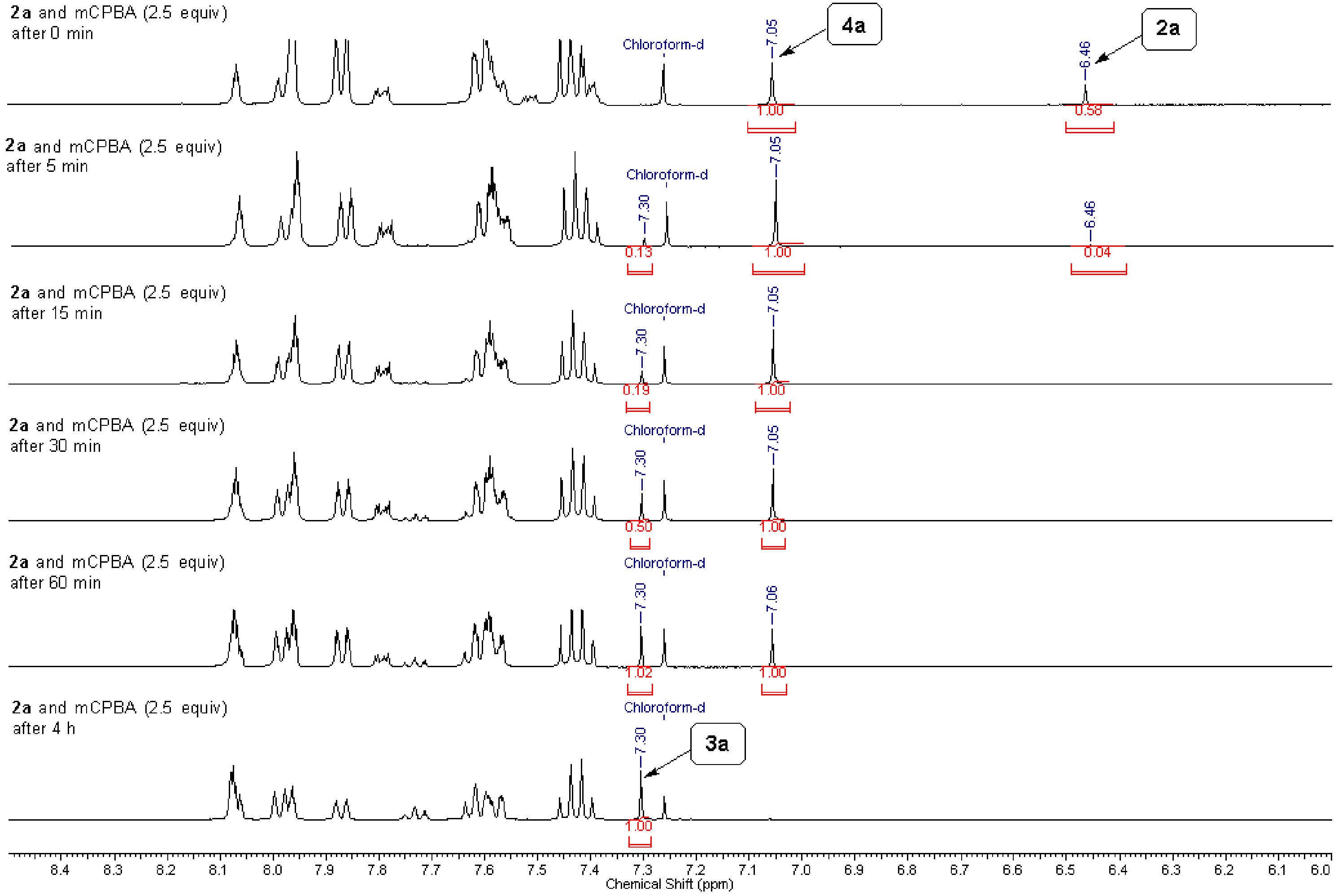

Figure S1. Time-dependent changes in ^{1}H NMR (400 MHz) spectra of the crude reaction mixture of **2a** and 2.5 equiv of mCPBA in $CDCl_3$.

General procedure for synthesis 2a–p

DIPEA (78 µL, 58 mg, 0.45 mmol), 5-nitroisoxazole **1** (0.29 mmol) and thiol (0.45 mmol) were mixed in MeCN (1 mL). The resulting mixture was stirred at rt for 24 h. After the reaction was finished, the mixture was poured into water (3 mL) and was extracted by CH_2Cl_2 (3 × 3 mL). Combined organic layers were washed successively with a saturated aqueous $NaHCO_3$ (5 mL), a saturated aqueous NaCl (5 mL) and dried over anhydrous $MgSO_4$. The solvent was removed under reduced pressure. The residue was purified by column chromatography using petroleum ether/ethyl acetate as the eluent to give the products **2a**–**p**.

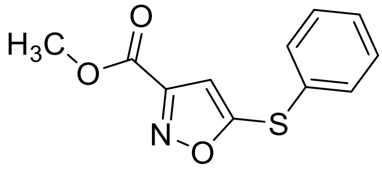

*Methyl 5-(phenylthio)isoxazole-3-carboxylate (**2a**)* [2]

Yield: 64 mg (93%); colorless oil; R_f = 0.20 (petroleum ether:EtOAc = 10:1).

^{1}H NMR (400 MHz, $CDCl_3$) δ 3.92 (s, 3H, CH_3O), 6.45 (s, 1H, CH), 7.34-7.41 (m, 3H, 3CH), 7.47-7.53 (m, 2H, 2CH).

^{13}C NMR (100 MHz, $CDCl_3$) δ 53.0 (CH_3O), 106.1 (CH), 129.3 (C), 129.6 (CH), 129.9 (2CH), 133.0 (2CH), 156.6 (C), 160.0 (C), 169.2 (C).

ESI-TOF *m/z*: [M+Na$^+$] calcd for $C_{11}H_9NNaO_3S^+$: 258.0195, found: 258.0196.

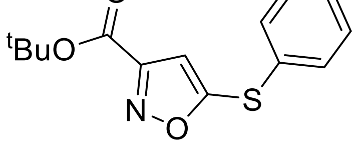

*tert-Butyl 5-(phenylthio)isoxazole-3-carboxylate (**2b**)* [2]

Yield: 68 mg (85%); colorless oil; R_f = 0.08 (petroleum ether:DCM = 2:1).

^{1}H NMR (400 MHz, $CDCl_3$) δ 1.58 (s, 9H, $3CH_3$), 6.45 (s, 1H, CH), 7.35-7.40 (m, 3H, 3CH), 7.46-7.52 (m, 2H, 2CH).

^{13}C NMR (100 MHz, $CDCl_3$) δ 28.1 ($3CH_3$), 84.0 (C-O), 106.7 (CH), 129.4 (CH), 129.8 (C), 129.9 (2CH), 132.8 (2CH), 158.2 (C), 158.7 (C), 168.3 (C).

ESI-TOF *m/z*: [$M+H^+$] calcd for $C_{14}H_{16}NO_3S^+$: 278.0845, found: 278.0848.

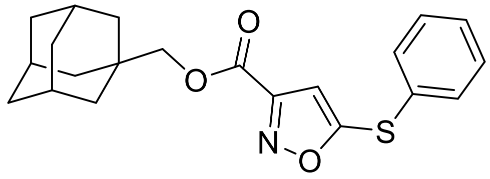

*(Adamantan-1-yl)methyl 5-(phenylthio)isoxazole-3-carboxylate (**2c**)* [2]

Yield: 122 mg (85%); colorless solid; R_f = 0.40 (petroleum ether:EtOAc = 20:1).

^{1}H NMR (400 MHz, $CDCl_3$) δ 1.57-1.61 (m, 6H, $3CH_2$), 1.62-1.69 (m, 3H, $3CH_2$), 1.69-1.76 (m, 3H, $3CH_2$), 1.96-2.02 (m, 3H, 3CH), 3.95 (s, 2H, CH_2O), 6.51 (s, 1H, CH), 7.36-7.41 (m, 3H, 3CH), 7.48-7.54 (m, 2H, 2CH).

^{13}C NMR (100 MHz, $CDCl_3$) δ 28.0 ($3CH_2$), 33.6 (C), 36.9 ($3CH_2$), 39.2 ($3CH_2$), 75.5 (CH_2O), 106.5 (CH), 129.48 (CH), 129.54 (C), 129.9 (2CH), 132.9 (2CH), 157.0 (C), 159.9 (C), 168.5 (C).

ESI-TOF *m/z*: [$M+NH_4^+$] calcd for $C_{21}H_{27}N_2O_3S^+$: 387.1737, found: 387.1735; m.p. = 73-74°C

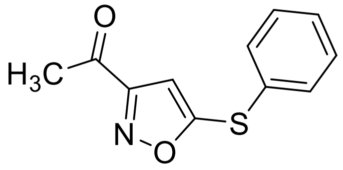

*1-(5-(Phenylthio)isoxazol-3-yl)ethan-1-one (**2d**)* [2]

Yield: 55 mg (86%); colorless oil; R_f = 0.41 (petroleum ether:EtOAc = 20:1).

^{1}H NMR (400 MHz, $CDCl_3$) δ 2.60 (s, 3H, CH_3), 6.41 (s, 1H, CH), 7.34-7.41 (m, 3H, 3CH), 7.47-7.54 (m, 2H, 2CH).

^{13}C NMR (100 MHz, $CDCl_3$) δ 27.2 (CH_3), 103.9 (CH), 129.4 (C), 129.6 (CH), 130.0 (2CH), 133.1 (2CH), 162.4 (C), 169.0 (C), 191.7 (C).

ESI-TOF *m/z*: [$M+H^+$] calcd for $C_{11}H_{10}NO_2S^+$: 220.0427, found: 220.0420.

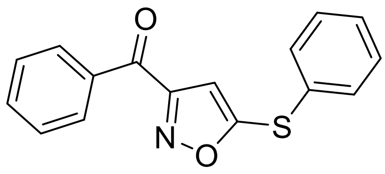

*Phenyl(5-(phenylthio)isoxazol-3-yl)methanone (**2e**)*

Yield: 64 mg (79 %); colorless oil, R_f = 0.14 (petroleum ether:DCM = 2:1).

^{1}H NMR (400 MHz, $CDCl_3$) δ 6.59 (s, 1H, CH), 7.36-7.44 (m, 3H, 3CH), 7.47- 7.53 (m, 2H, 2CH), 7.54-7.60 (m, 2H, 2CH), 7.60-7.66 (m, 1H, CH), 8.24-8.30 (m, 2H, 2CH).

^{13}C NMR (100 MHz, $CDCl_3$) δ 106.2 (CH), 128.6 (2CH), 129.4 (C), 129.6 (CH), 129.9 (2CH), 130.7 (2CH), 133.1 (2CH), 134.2 (CH), 135.5 (C), 162.3 (C), 168.1 (C), 185.2 (C).

ESI-TOF *m/z*: [$M+H^+$] calcd for $C_{16}H_{12}NO_2S^+$: 282.0583, found: 282.0585.

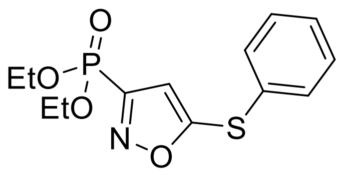

*Diethyl (5-(phenylthio)isoxazole-3-carbonyl)phosphonate (**2f**)* [2]

Yield: 81 mg (89%); colorless oil; R_f = 0.23 (petroleum ether:EtOAc = 2:1).

^{1}H NMR (400 MHz, $CDCl_3$) δ 1.34 (dt, $^3J_{HH}$ = 7.1 Hz, $^4J_{PH}$ = 0.7 Hz, 6H, $2CH_3$), 4.13-4.28 (m, 4H, $2CH_2O$), 6.26 (d, $^3J_{PH}$ = 0.9 Hz, 1H, CH), 7.35-7.43 (m, 3H, 3CH), 7.48-7.55 (m, 2H, 2CH).

^{13}C NMR (100 MHz, $CDCl_3$) δ 16.3 ($^3J_{CP}$ = 6.5 Hz, $2CH_3$), 63.9 ($^2J_{CP}$ = 5.9 Hz, $2CH_2$), 107.1 ($^2J_{CP}$ = 20.3 Hz, CH), 129.0 (C), 129.8 (CH), 130.0 (2CH), 133.4 (2CH), 156.4 ($^1J_{CP}$ = 210.8 Hz, C), 168.6 ($^3J_{CP}$ = 11.6 Hz, C).

^{31}P NMR (162 MHz, $CDCl_3$) δ 3.92

ESI-TOF *m/z*: [M+Na$^+$] calcd for $C_{13}H_{16}NNaO_4PS^+$: 336.0430, found: 336.0430.

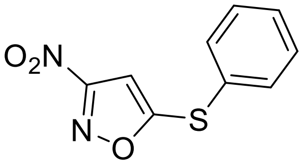

*3-Nitro-5-(phenylthio)isoxazole (**2g**)* [2]

Yield: 58 mg (90%); colorless oil; R_f = 0.35 (petroleum ether:DCM = 2:1).

^{1}H NMR (400 MHz, $CDCl_3$) δ 6.49 (s, 1H, CH), 7.45-7.50 (m, 3H, 3CH), 7.57-7.63 (m, 2H, 2CH).

^{13}C NMR (100 MHz, $CDCl_3$) δ 99.6 (CH), 127.3 (C), 130.4 (2CH), 130.7 (CH), 134.1 (2CH), 167.6 (C), 174.0 (C).

ESI-TOF *m/z*: [M+Na$^+$] calcd for $C_9H_6N_2NaO_3S^+$: 244.9991, found: 245.0005.

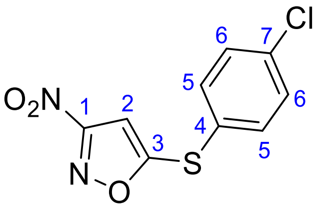

*5-((4-Chlorophenyl)thio)-3-nitroisoxazole (**2h**)*

Yield: 56 mg (75%), yellow oil, R_f = 0.44 (petroleum ether:DCM = 3:2)

^{1}H NMR (400 MHz, $CDCl_3$): δ 6.57 (s, 1H, C^2H), 7.40-7.45 (m, 2H, $2C^6H$), 7.50-7.55 (m, 2H, $2C^5H$).

^{13}C NMR (100 MHz, $CDCl_3$) δ 100.1 (C^2H), 125.7 (C^4), 130.6 ($2C^6H$), 135.3 ($2C^5H$), 137.3 (C^7), 167.6 (C^1-NO_2), 172.7 (C^3)

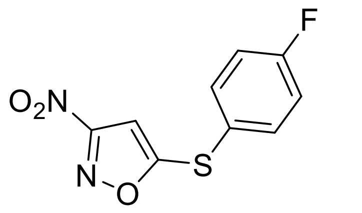

*5-((4-Fluorophenyl)thio)-3-nitroisoxazole (**2i**)*

Yield: 45 mg (65%), brown oil, R_f = 0.16 (petroleum ether:EtOAc = 20:1) ^{1}H NMR (400 MHz, $CDCl_3$) δ 6.50 (s, 1H, CH), 7.13-7.21 (m, 2H, 2CH,), 7.59-7.65 (m, 2H, 2CH).

^{13}C NMR (100 MHz, $CDCl_3$) δ 99.5 (CH), 117.7 ($^{2}J_{CF}$ = 22.0 Hz, 2CH), 122.3 ($^{4}J_{CF}$ = 3.9 Hz, C), 136.7 ($^{3}J_{CF}$ = 9.1 Hz, 2CH), 164.3 ($^{1}J_{CF}$ = 253.5 Hz, C), 167.6 (C), 173.5 (C).

^{19}F NMR (376 MHz, $CDCl_3$) δ -108.40 (tt, F, $^{4}J_{FH}$=5.1, $^{3}J_{FH}$ = 8.2 Hz).

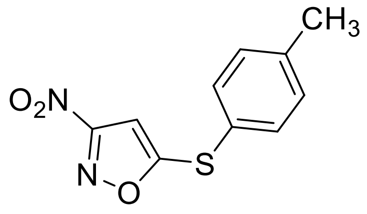

*3-Nitro-5-(p-tolylthio)isoxazole (**2j**)*

Yield: 41 mg (60%), light-yellow solid, R_f = 0.42 (petroleum ether:DCM = 3:2)

^{1}H NMR (400 MHz, $CDCl_3$) δ 2.41 (s, 3H, CH_3), 6.40 (s, 1H, CH), 7.26-7.30 (m, 2H, 2CH), 7.46-7.52 (m, 2H, 2CH).

^{13}C NMR (100 MHz, $CDCl_3$) δ 21.5 (CH_3), 98.9 (CH), 123.6 (C), 131.2 (2CH), 134.5 (2CH), 141.5 (C), 167.6 (C), 174.9 (C).

ESI-TOF *m/z*: [M+H$^+$] calcd for $C_{10}H_9N_2O_3S^+$: 237.0330, found: 237.0328; m.p. = 53-55°C.

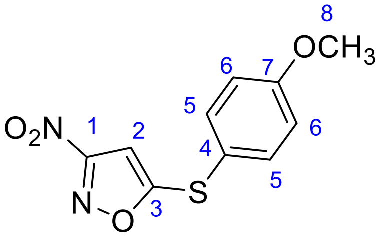

*5-((4-Methoxyphenyl)thio)-3-nitroisoxazole (**2k**)*

Yield: 51 mg (70%), brown-yellow oil, R_f = 0.22 (petroleum ether:EtOAc = 15:1)

^1H NMR (400 MHz, $CDCl_3$) δ 3.86 (s, 3H, C^8H_3), 6.34 (s, 1H, C^2H), 6.95-7.01 (m, 2H, C^6H), 7.52-7.58 (m, 2H, $2C^5H$).

^{13}C NMR (100 MHz, $CDCl_3$) δ 55.7 (C^8H_3), 98.3 (C^2H), 116.0 ($2C^6H$), 117.0 (C^4), 136.6 ($2C^5H$), 161.9 (C^7), 167.5 (C^1-NO_2), 175.6 (C^3).

ESI-TOF *m/z*: [M+H$^+$] calcd for $C_{10}H_9N_2O_4S^+$: 253.0273, found: 253.0278.

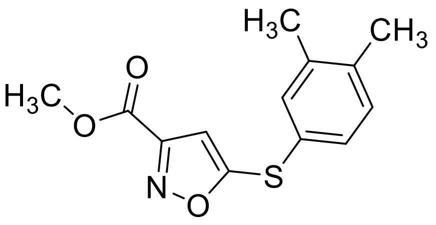

*Methyl 5-((3,4-dimethylphenyl)thio)isoxazole-3-carboxylate (**2l**)*

Yield: 53 mg (70%), light-yellow solid, R_f = 0.64 (petroleum ether:EtOAc = 5:1)
^1H NMR (400 MHz, $CDCl_3$) δ 2.24 (s, 3H, CH_3), 2.26 (s, 3H, CH_3), 3.92 (s, 3H, CH_3), 6.34 (s, 1H, CH), 7.14 (d, $^3J_{HH}$ = 7.7 Hz, 1H, CH), 7.26 (dd, $^3J_{HH}$ = 7.7, $^4J_{HH}$ = 2.1 Hz), 7.30 (d, $^3J_{HH}$ = 2.1 Hz).

^{13}C NMR (100 MHz, $CDCl_3$) δ 19.6 (CH_3), 19.7 (CH_3), 52.9 (CH_3), 105.0 (CH), 125.3 (C), 131.1 (2CH), 134.6 (CH), 138.6 (C), 139.0 (C), 156.5 (C), 160.1 (C), 170.4 (C).

ESI-TOF *m/z*: [M+H$^+$] calcd for $C_{13}H_{14}NO_3S^+$: 264.0689, found: 264.0684; m.p. = 57-59°C

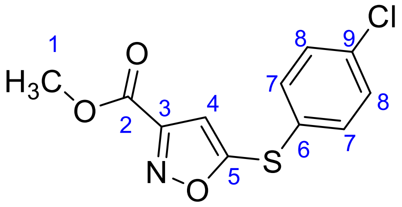

*Methyl 5-((4-chlorophenyl)thio)isoxazole-3-carboxylate (**2m**)*

Yield: 59 mg (75%), colorless solid, R_f = 0.21 (petroleum ether:EtOAc = 10:1)

^1H NMR (400 MHz, $CDCl_3$) δ 3.96 (s, 3H, C^1H_3), 6.52 (s, 1H, C^4H), 7.34-7.40 (m, 2H, $2C^8H$), 7.43-7.48 (m, 2H, $2C^7H$).

^{13}C NMR (100 MHz, $CDCl_3$) δ 53.1 (C^1H_3), 106.6 (C^4H), 127.9 (C^6), 130.2 ($2C^8H$), 134.3 ($2C^7H$), 136.2 (C^9), 156.8 (C^3), 160.0 (C^2), 168.2 (C^5).

ESI-TOF *m/z*: [M+H$^+$] calcd for $C_{11}H_9ClNO_3S^+$: 269.9986, found: 269.9981; m.p. = 47-49 °C.

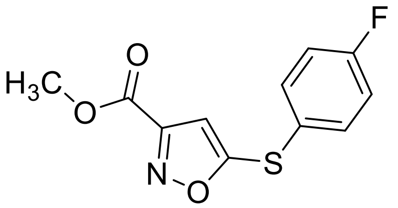

*Methyl 5-((4-fluorophenyl)thio)isoxazole-3-carboxylate (**2n**)*

Yield: 49 mg (67%), yellow oil, R_f = 0.32 (petroleum ether:EtOAc = 5:1)

^1H NMR (400 MHz, $CDCl_3$) δ 3.94 (s, 3H, CH_3), 6.42 (s, 1H, CH), 7.07-7.14 (m, 2H, 2CH), 7.52-7.58 (m, 2H, 2CH).

^{13}C NMR (100 MHz, $CDCl_3$) δ 53.1 (CH_3), 105.6 (CH), 117.3 (2CH, $^3J_{CF}$ = 22.3 Hz), 124.2 (C, $^4J_{CF}$ = 3.3 Hz), 135.9 (2CH, $^2J_{CF}$ = 8.7 Hz), 156.7 (C), 160.0 (C) 163.8 (C, $^1J_{CF}$ = 251 Hz), 169.2 (C).

^{19}F NMR (376 MHz, $CDCl_3$) δ -108.40 (tt, F, $^4J_{FH}$=5.1 Hz, $^3J_{FH}$=8.2 Hz).

ESI-TOF *m/z*: [M+H$^+$] calcd for $C_{11}H_9FNO_3S^+$: 254.0282, found: 254.0281.

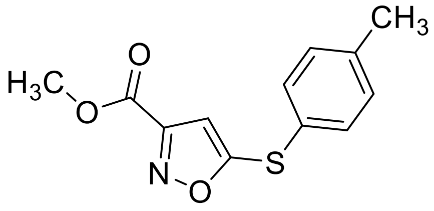

*Methyl 5-(p-tolylthio)isoxazole-3-carboxylate (**2o**)*

Yield: 46 mg (63%), yellow oil, R_f = 0.39 (petroleum ether:EtOAc = 5:1)

^1H NMR (400 MHz, $CDCl_3$) δ 2.36 (s, 3H, CH_3), 3.91 (s, 3H, CH_3), 6.35 (s, 1H, CH), 7.16-7.22 (m, 2H, 2CH), 7.38-7.44 (m, 2H, 2CH).

^{13}C NMR (100 MHz, $CDCl_3$) δ 21.3 (CH_3), 52.9 (CH_3), 105.1 (CH), 125.3 (C), 130.7 (2CH), 133.6 (2CH), 140.2 (C), 156.5 (C), 160.0 (C), 170.2 (C)

ESI-TOF *m/z*: [M+H$^+$] calcd for $C_{12}H_{12}NO_3S^+$: 250.0532, found: 250.0530.

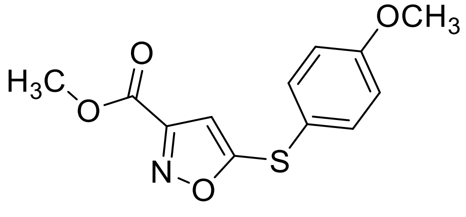

*Methyl 5-((4-methoxyphenyl)thio)isoxazole-3-carboxylate (**2p**)*

Yield: 50 mg (70%), yellow oil, R_f = 0.23 (petroleum ether:EtOAc = 5:1)

^1H NMR (400 MHz, $CDCl_3$) δ 3.90 (s, 3H, CH_3), 6.24 (s, 1H, CH), 6.87-6.94 (m, 2H, 2CH), 7.45-7.52 (m, 2H, 2CH).

^{13}C NMR (100 MHz, $CDCl_3$) δ 52.9 (CH_3), 55.5 (CH_3), 104.1 (CH), 115.5 (2CH), 118.7 (C), 136.0 (2CH), 156.4 (C), 160.1 (C), 161.2 (C), 171.1 (C)

ESI-TOF *m/z*: [M+NH_4^+] calcd for $C_{12}H_{15}N_2O_4S^+$: 283.0747, found: 283.0743.

General procedure for synthesis 3a–p

MCPBA (0.5 mmol, 167 mg, 70%) was added to the solution of **2** (0.2 mmol) in DCM (2 mL). The reaction mixture was stirred for 4 h at room temperature. Then the resulting mixture was poured into water (6 mL) and extracted with DCM (3 × 10 mL). Combined organic layers were washed with saturated aqueous $NaHCO_3$ (9 mL) and saturated aqueous NaCl (9 mL) and dried over anhydrous $MgSO_4$. The solvent was evaporated under reduced pressure and the residue was purified by column chromatography using petroleum ether/ethyl acetate as the eluent to give the products **3a–p**.

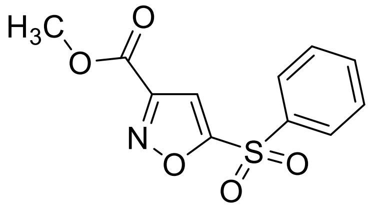

*Methyl 5-(phenylsulfonyl)isoxazole-3-carboxylate (**3a**)*

Yield: 50 mg (93%); colorless solid; R_f = 0.16 (petroleum ether:EtOAc = 5:1).

^{1}H NMR (400 MHz, $CDCl_3$) δ 3.97 (s, 3H, CH_3), 7.29 (s, 1H, CH), 7.58-7.65 (m, 2H, 2CH), 7.70-7.76 (m, 1H, CH), 8.04-8.09 (m, 2H, 2CH).

^{13}C NMR (100 MHz, $CDCl_3$) δ 53.5 (CH_3), 108.9 (CH), 128.8 (2CH), 130.0 (2CH), 135.5 (CH), 137.6 (C), 156.4 (C), 158.8 (C), 169.2 (C).

ESI-TOF *m/z*: [M+H^+] calcd for $C_{11}H_{11}NO_5S^+$: 268.0274, found 268.0275; m.p = 73-74°C.

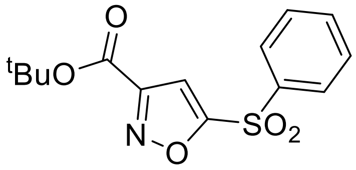

*tert-Butyl 5-(phenylsulfonyl)isoxazole-3-carboxylate (**3b**)*

Yield: 51 mg (83%); colorless solid, R_f = 0.35 (petroleum ether:EtOAc = 5:1)

^{1}H NMR (400 MHz, $CDCl_3$) δ 1.58 (s, 9H, $3CH_3$), 7.22 (s, 1H, CH), 7.57-7.64 (m, 2H, 2CH), 7.69-7.75 (m, 1H, CH), 8.03-8.09 (m, 2H, 2CH).

^{13}C NMR (100 MHz, $CDCl_3$) δ 28.1 ($3CH_3$), 85.0 (C), 108.9 (CH), 128.8 (2CH), 129.9 (2CH), 135.4 (CH), 137.8 (C), 157.3 (C), 157.8 (C), 168.8 (C).

ESI-TOF *m/z*: [M+Na$^+$] calcd for $C_{14}H_{15}NNaO_5S^+$: 332.0563, found: 332.0564; m.p. = 87-88°C.

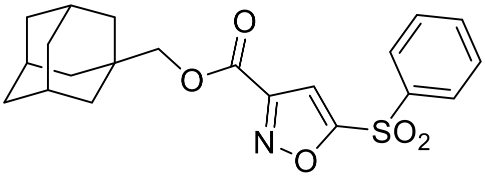

*(Adamantan-1-yl)methyl 5-(phenylsulfonyl)isoxazole-3-carboxylate (**3c**)*

Yield: 72 mg (83%); colorless solid; R_f = 0.36 (petroleum ether:EtOAc = 5:1).

^{1}H NMR (400 MHz, $CDCl_3$) δ 1.55-1.59 (m, 6H, $3CH_2$), 1.61-1.68 (m, 3H, $3CH_2$), 1.69-1.77 (m, 3H, $3CH_2$), 1.96-2.03 (m, 3H, 3CH), 3.96 (s, 2H, CH_2O), 7.28 (s, 1H, CH), 7.59-7.65 (m, 2H, 2CH), 7.70-7.76 (m, 1H, CH), 8.05-8.10 (m, 2H, 2CH).

^{13}C NMR (100 MHz, $CDCl_3$) δ 28.0 (3CH), 33.5 (C), 36.9 ($3CH_2$), 39.2 ($3CH_2$), 76.1 (C), 108.8 (CH), 128.9 (2CH), 130.0 (2CH), 135.4 (CH), 137.6 (C), 156.7 (C), 158.5 (C), 169.1 (C).

ESI-TOF *m/z*: $[M+NH_4^+]$ calcd for $C_{21}H_{27}N_2O_5S^+$: 419.1635, found: 419.0632; m.p. = 151-152°C.

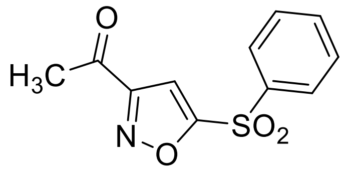

*1-(5-(Phenylsulfonyl)isoxazol-3-yl)ethan-1-one (**3d**)*

Yield: 41 mg (82%); colorless solid; R_f = 0.31 (petroleum ether:EtOAc = 5:1).

^{1}H NMR (400 MHz, $CDCl_3$) δ 2.63 (s, 3H, CH_3), 7.23 (s, 1H, CH), 7.58-7.65 (m, 2H, 2CH), 7.70-7.76 (m, 1H, CH), 8.04-8.09 (m, 2H, 2CH).

^{13}C NMR (100 MHz, $CDCl_3$) δ 27.5 (CH_3), 106.9 (CH), 128.8 (2CH), 130.0 (2CH), 135.4 (CH), 137.7 (C), 161.7 (C), 169.1 (C), 190.2 (C).

ESI-TOF *m/z*: $[M+K^+]$ calcd for $C_{11}H_9NKO_4S^+$: 289.9884, found: 289.9886; m.p. = 76-77°C.

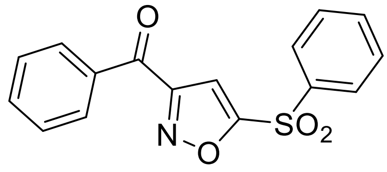

*Phenyl(5-(phenylsulfonyl)isoxazol-3-yl)methanone (**3e**)*

Yield: 57 mg (91%), light-yellow oil, R_f = 0.1 (petroleum ether:EtOAc = 10:1)

^{1}H NMR (400 MHz, $CDCl_3$) δ 7.40 (s, 1H, CH), 7.49-7.56 (m, 2H, 2CH), 7.60-7.71 (m, 3H, 3CH), 7.72-7.78 (m, 1H, CH), 8.09-8.15 (m, 2H, 2CH), 8.22-8.27 (m, 2H, 2CH).

^{13}C NMR (100 MHz, $CDCl_3$) δ 109.5 (CH), 128.9 (2CH), 129.0 (2CH) 130.0 (2CH), 130.8 (2CH), 134.87 (CH), 134.91 (C), 135.4 (CH), 137.8 (C), 161.7 (C), 168.4 (C), 183.9 (C), 184.9 (C).

ESI-TOF *m/z*: [M+H$^+$] calcd for $C_{16}H_{12}NO_4S^+$: 314.0482, found: 314.0480.

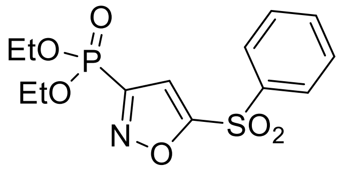

*Diethyl (5-(phenylsulfonyl)isoxazol-3-yl)phosphonate (**3f**)*

Yield: 61 mg (88%), light-yellow oil, R_f = 0.37 (petroleum ether:EtOAc = 1:1)

^{1}H NMR (400 MHz, $CDCl_3$) δ 1.37 (dt, 6H, 2CH_3, $^4J_{PH}$ = 0.6 Hz, $^3J_{HH}$ = 7.0 Hz), 4.18-4.33 (m, 4H, 2CH_2), 7.15 (d, 1H, CH, $^3J_{PH}$ = 0.8 Hz), 7.58-7.66 (m, 2H, 2CH), 7.70-7.77 (m, 1H, CH) 8.03-8.10 (m, 2H, 2CH).

^{13}C NMR (100 MHz, $CDCl_3$) δ 16.3 (d, 2CH_3, $^3J_{PC}$ = 6.3 Hz), 64.4 (d, 2CH_2, $^2J_{PC}$ = 6.1 Hz), 110.7 (d, CH, $^2J_{PC}$ = 19.4 Hz), 128.9 (2CH), 130.0 (2CH), 135.5 (CH), 137.7 (C), 156.7 (d, C, $^1J_{PC}$ = 211.6 Hz), 168.3 (d, C, $^3J_{PH}$ = 10.0 Hz).

^{31}P NMR (162 MHz, $CDCl_3$) δ 1.67.

Anal. calcd for $C_{13}H_{16}NO_6PS$: C, 45.22; H, 4.67; N, 4.06. Found: C, 45.39; H, 4.61; N, 4.12

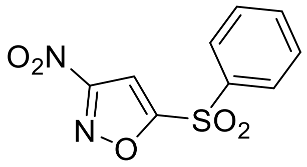

*3-Nitro-5-(phenylsulfonyl)isoxazole (**3g**)*

Yield: 44 mg (86%), colorless solid; R_f = 0.28 (petroleum ether:EtOAc = 5:1)

^{1}H NMR (400 MHz, $CDCl_3$) δ 7.44 (s, 1H, CH), 7.63-7.71 (m, 2H, 2CH), 7.76-7.83 (m, 1H, CH), 8.07-8.13 (m, 2H, 2CH).

^{13}C NMR (100 MHz, $CDCl_3$) δ 103.6 (CH), 129.2 (2CH), 130.3 (2CH), 136.2 (CH), 136.7 (C), 166.9 (C), 172.1 (C).

Anal. calcd for $C_9H_6N_2O_5S$: C, 42.52; H, 2.38; N, 11.02. Found: C, 42.54; H, 2.44; N, 11.03; m.p. = 101-102°C.

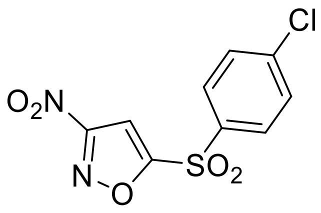

*5-((4-Chlorophenyl)sulfonyl)-3-nitroisoxazole (**3h**)*

Yield: 46 mg (80%), colorless solid; R_f = 0.43 (petroleum ether:EtOAc = 5:1)

^{1}H NMR (400 MHz, $CDCl_3$) δ 7.45 (s, 1H, CH), 7.62-7.67 (m, 2H, 2CH), 8.01-8.06 (m, 2H, 2CH).

^{13}C NMR (100 MHz, $CDCl_3$) δ 103.8 (CH), 130.6 (2CH), 130.7 (2CH), 135.0 (C), 143.5 (C), 166.9 (C-NO_2), 171.6 (C).

Anal. calcd for $C_9H_5ClN_2O_5S$: C, 37.45; H, 1.75; N, 9.70. Found: C, 37.43; H, 1.81; N, 9.69; m.p. = 109-111°C.

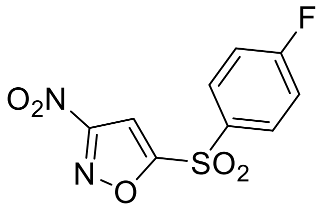

*5-((4-Fluorophenyl)sulfonyl)-3-nitroisoxazole (**3i**)*

Yield: 42 mg (78%), light-yellow solid; R_f = 0.53 (petroleum ether:EtOAc = 3:1)

^{1}H NMR (400 MHz, $CDCl_3$) δ 7.31-7.38 (m, 2H, 2CH), 7.44 (s, 1H, CH), 8.10-8.17 (m, 2H, 2CH).

^{13}C NMR (100 MHz, $CDCl_3$) δ 103.6 (CH), 117.9 ($^2J_{FC}$ = 23.0 Hz, 2CH), 132.4 ($^3J_{FC}$ = 10.1 Hz, 2CH), 132.6 ($^4J_{FC}$ = 3.1 Hz, C), 166.9 (C-NO_2), 167.4 ($^1J_{FC}$ = 261.3 Hz, C), 171.9 (C).

^{19}F NMR (376 MHz, $CDCl_3$) δ -98.25 (tt, $^3J_{FH}$ = 8.0 Hz, $^4J_{FH}$ = 4.9 Hz)

Anal. calcd for $C_9H_5FN_2O_5S$: C, 39.71; H, 1.85; N, 10.29. Found: C, 39.69; H, 1.78; N, 10.27; m.p. 108-110 °C.

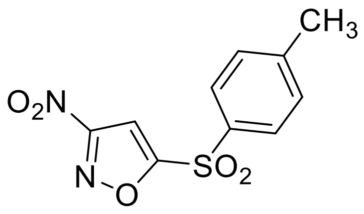

*3-Nitro-5-tosylisoxazole (**3j**)*

Yield: 40 mg (75%), light-yellow solid; R_f = 0.18 (petroleum ether:EtOAc = 10:1)

^{1}H NMR (400 MHz, $CDCl_3$) δ 2.48 (s, 3H, CH_3), 7.40 (s, 1H, CH), 7.42-7.48 (m, 2H, 2CH), 7.93-7.99 (m, 2H, 2CH).

^{13}C NMR (100 MHz, $CDCl_3$) δ 22.0 (CH_3), 103.2 (CH), 129.2 (2CH), 130.9 (2CH), 133.6 (C), 147.9 (C), 166.9 (C-NO_2), 172.4 (C).

ESI-TOF *m/z*: [M+Na$^+$] calcd for $C_{10}H_8N_2NaO_5S^+$: 291.0046, found: 291.0043; m.p. = 127-129°C.

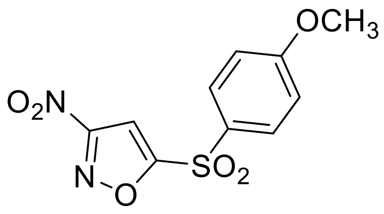

*5-((4-Methoxyphenyl)sulfonyl)-3-nitroisoxazole (**3k**)*

Yield: 47 mg (82%), white solid; R_f = 0.36 (petroleum ether:EtOAc = 3:1)

^{1}H NMR (400 MHz, $CDCl_3$) δ 3.92 (s, 3H, CH_3), 7.06-7.12 (m, 2H, 2CH), 7.37 (s, 1H, CH), 7.98-8.04 (m, 2H, 2CH).

^{13}C NMR (100 MHz, $CDCl_3$) δ 56.2 (CH_3), 102.8 (CH), 115.6 (2CH), 127.5 (C), 131.7 (2CH), 165.8 (C), 166.9 ($C-NO_2$), 172.8 (C).

ESI- *m/z*: [$M+NH_4^+$] calcd for $C_{10}H_{12}N_3O_6S^+$: 303.0474, found: 303.0474; m.p. = 113-115°C

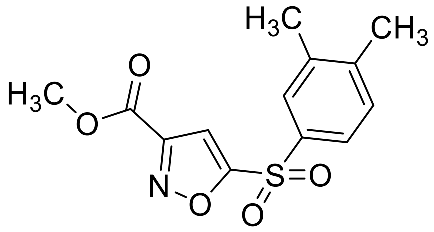

*Methyl 5-((3,4-dimethylphenyl)sulfonyl)isoxazole-3-carboxylate (**3l**)*

Yield: 47 mg (79%), white solid; R_f = 0.45 (petroleum ether:EtOAc = 3:1)

^{1}H NMR (400 MHz, $CDCl_3$) δ 2.33-2.37 (m, 6H, $2CH_3$), 3.98 (s, 3H, CH_3), 7.25 (s, 1H, CH), 7.33-7.38 (m, 1H, CH), 7.76-7.81 (m, 2H, 2CH).

^{13}C NMR (100 MHz, $CDCl_3$) δ 20.0 (CH_3), 20.4 (CH_3), 53.5 (CH_3), 108.4 (CH), 126.5 (CH), 129.5 (CH), 131.1 (CH), 134.6 (C), 139.1 (C), 145.8 (C), 156.3 (C), 159.0 (C), 169.8 (C)

ESI-TOF *m/z*: [$M+H^+$] calcd for $C_{13}H_{14}NO_5S^+$: 296.0587, found: 296.0583; m.p. = 105-107°C.

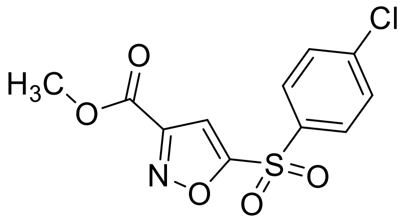

*Methyl 5-((4-chlorophenyl)sulfonyl)isoxazole-3-carboxylate (**3m**)*

Yield: 49 mg (82%), colorless solid; R_f = 0.24 (petroleum ether:EtOAc = 4:1)

^1H NMR (400 MHz, $CDCl_3$) δ 3.99 (s, 3H, CH_3), 7.31 (s, 1H, CH), 7.58-7.63 (m, 2H, 2CH), 7.99-8.04 (m, 2H, 2CH).

^{13}C NMR (100 MHz, $CDCl_3$) δ 53.5 (CH_3), 106.6 (CH), 126.4 (2CH), 130.4 (2CH), 139.1 (C), 139.4 (C), 156.4 (C), 159.2 (C), 174.4 (C).

ESI-TOF *m/z*: [M+H^+] calcd for $C_{11}H_9ClNO_5S^+$: 301.9884, found 301.9888; m.p. = 110-112°C.

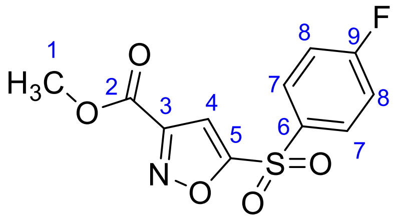

*Methyl 5-((4-fluorophenyl)sulfonyl)isoxazole-3-carboxylate (**3n**)*

Yield: 40 mg (70%), colorless solid; R_f = 0.18 (petroleum ether:EtOAc = 5:1)

^1H NMR (400 MHz, $CDCl_3$) δ 3.99 (s, 3H, C^1H_3), 7.27-7.34 (m, 3H, $2C^8H$+C^4H), 8.08-8.14 (m, 2H, $2C^7H$).

^{13}C NMR (100 MHz, $CDCl_3$) δ 53.6 (C^1H_3), 108.9 (C^4H), 117.5 ($^2J_{CF}$ = 23.0 Hz, $2C^8H$), 132.0 ($^3J_{CF}$ = 10.1 Hz, $2C^7H$), 133.6 (C^6), 156.4 (C^3), 158.8 (C^2), 167.0 ($^1J_{CF}$ = 259.8 Hz, C^9), 169.1 (C^5)

^{19}F NMR (376 MHz, $CDCl_3$) δ -108.40 (tt, $^3J_{FH}$=5.2 Hz, $^4J_{FH}$=8.2 Hz).

ESI-TOF *m/z*: [M+H^+] calcd for $C_{11}H_9FNO_5S^+$: 286.0180, found: 286.0179; m.p. = 90-92°C.

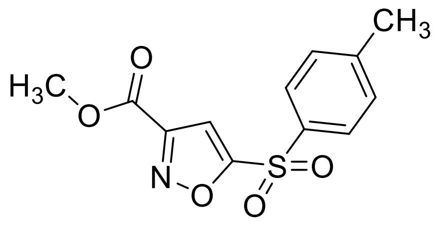

*Methyl 5-tosylisoxazole-3-carboxylate (**3o**)*

Yield: 44 mg (78%), colorless solid; R_f = 0.40 (petroleum ether:EtOAc = 3:1)

^{1}H NMR (400 MHz, $CDCl_3$) δ 2.46 (s, 3H, CH_3), 3.97 (s, 3H, CH_3), 7.25 (s, 1H, CH), 7.38-7.43 (m, 2H, 2CH), 7.92-7.96 (m, 2H, 2CH).

^{13}C NMR (100 MHz, $CDCl_3$) δ 21.9 (CH_3), 53.5 (CH_3), 108.5 (CH), 128.9 (2CH), 130.6 (2CH), 134.5 (C), 147.0 (C), 156.3 (C), 158.9 (C), 169.6 (C).

ESI-TOF *m/z*: [M+Na$^+$] calcd for $C_{12}H_{11}NNaO_5S^+$: 291.0046, found: 291.0043; m.p. = 95-97°C.

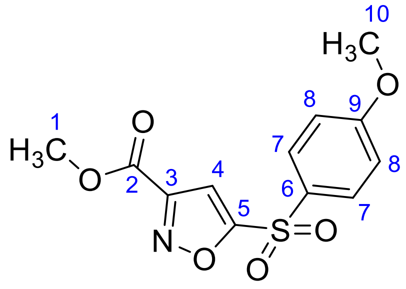

*Methyl 5-((4-methoxyphenyl)sulfonyl)isoxazole-3-carboxylate (**3p**)*

Yield: 49 mg (83%), colorless solid; R_f = 0.33 (petroleum ether:EtOAc = 5:1)

^{1}H NMR (400 MHz, $CDCl_3$) δ 3.90 (s, 3H, $C^{10}H_3$), 3.98 (s, 3H, C^1H_3), 7.02-7.09 (m, 2H, 2C^8H), 7.23 (s, 1H, C^4H), 7.96-8.02 (m, 2H, 2C^7H).

^{13}C NMR (100 MHz, $CDCl_3$) δ 53.5 (C^1H_3), 56.1 ($C^{10}H_3$), 108.1 (C^4H), 115.3 (2C^8H), 128.7 (C^6), 131.4 (2C^7H), 156.3 (C^3), 159.0 (C^9), 165.3 (C^2), 170.0 (C^5).

ESI-TOF *m/z*; [$M+NH_4^+$] calcd for $C_{12}H_{15}N_2O_6S^+$: 315.0645, found: 315.0645; m.p. = 139-141°C.

General procedure for synthesis 4a, 4b

MCPBA (0.24 mmol, 59 mg, 70%) was added to the solution of **2a** or **2g** (0.2 mmol) in DCM (2 mL). The reaction mixture was stirred for 24 h at room temperature. Then the resulting mixture was poured into water (6 mL) and extracted with DCM (3 × 10 mL). Combined organic layers were washed with saturated aqueous $NaHCO_3$ (9 mL) and saturated aqueous NaCl (9 mL) and dried over anhydrous $MgSO_4$. The solvent was evaporated under reduced pressure and the residue was purified by column chromatography using petroleum ether/ethyl acetate as the eluent to give the products **4a** and **4b**.

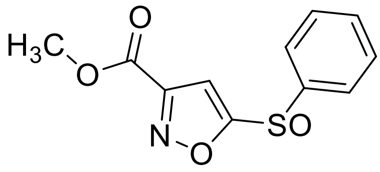

*Methyl 5-(phenylsulfinyl)isoxazole-3-carboxylate (**4a**)*

Yield: 37 mg (74%), colorless solid; R_f = 0.23 (petroleum ether:EtOAc = 3:1)

^1H NMR (400 MHz, $CDCl_3$) δ 3.95 (s, 3H, CH_3), 7.01 (s, H, CH), 7.53-7.61 (m, 3H, 3CH), 7.75-7.81 (m, 2H, 2CH).

^{13}C NMR (100 MHz, $CDCl_3$) δ 53.4 (CH_3), 106.4 (CH), 125.0 (2CH), 130.0 (2CH), 132.9 (CH), 140.6 (C), 156.3 (C), 159.3 (C), 174.9 (C).

ESI-TOF *m/z*: [$M+H^+$] calcd for $C_{11}H_{11}NO_4S^+$: 252.0325, found: 252.0332; m.p. = 66-67°C.

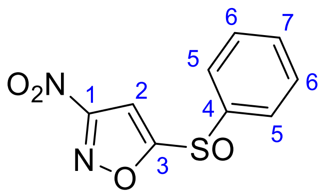

*3-nitro-5-(phenylsulfinyl)isoxazole (**4b**)*

Yield: 36 mg (75%), colorless solid; R_f = 0.19 (petroleum ether:EtOAc = 5:1)

^{1}H NMR (400 MHz, $CDCl_3$) δ 7.19 (s, H, C^2H), 7.57-7.67 (m, 3H, 3C^{6+7}H), 7.78-7.85 (m, 2H, 2C^5H).

^{13}C NMR (100 MHz, $CDCl_3$) δ 101.3 (C^2H), 125.0 (2C^5H), 130.4 (2C^6H), 133.5 (C^7H), 140.0 (C^4), 167.1 (br.s, C^1-NO_2), 178.8 (C^3).

Anal. calcd for $C_9H_6N_2O_4S$: C, 45.38; H, 2.54; N, 11.76. Found: C, 46.00; H, 2.40; N, 11.28; m.p. = 68-69°C.

Biology

Antibacterial activity

The antibacterial activity of the samples was evaluated using reporter strains *E. Coli lptD^{mut} pDualrep2 (KanR)* and *JW5503 ΔtolC pDualRep2* as previously described [3, 4]. 3 µL of a 100 mM DMSO solutions of the samples were applied to agar plates seeded with the reporter strains along with two control antibiotics: erythromycin (Ery, 5 mg/mL), inhibitor of protein synthesis, and levofloxacin (Lev, 25 mg/mL), inhibitor of DNA gyrase. Plates were incubated at 37 °C overnight and then scanned by ChemiDoc (Bio-Rad) in the modes 'Cy3-blot' for RFP and 'Cy5-blot'. In case of antibiotic-induced SOS-response activation of reporter strains' cells the expression of the *rfp* gene occurred, and disruption of translation mechanism led to the expression of *katushka2S* gene. While scanning, the signal from the RFP protein was displayed in green color, and from Katushka2S in red.

In vitro assays inhibitors of DNA gyrase, polymerase topoisomerase I and IV activities

Protein isolation

E. coli strain K-12 from the ASKA(-) [5] collection with a plasmid having a corresponding gene tagged at its N terminus by six histidine residues was used to obtain *E. coli* GyrA, GyrB, ParC, ParE and TopA.

For overexpression of proteins, 3 mL of an overnight culture of a strain with a corresponding plasmid in LB broth with 34 µg/mL of chloramphenicol (Cm) was diluted in 300 mL of LB broth with 34 µg/mL of Cm and grown at 37 °C with shaking at 200 rpm till the mid-log phase. Then overexpression was induced by adding 1 mM isopropyl-β-D-thiogalactopyranoside (IPTG) and continued overnight (16 h) at 18 °C with shaking at 200 rpm. This overnight cell culture was twice washed with phosphate-buffered saline (PBS) and centrifuged at 10000g for 10 min at 4 °C.

Then cell pellets were resuspended in 10 mL of lysis buffer (20 mM Tris-HCl pH 7.5, 500 mM NaCl, 5 mM imidazole, 7 mM β-mercaptoethanol, 1x Halt Protease Inhibitor Cocktail, 0.5 mg/mL lysozyme, 0.1% (v/v) Triton X-100, 5% (v/v) glycerol, DNase I 1U/mL, Milli-Q (MQ) ultrapure water), incubated for 15 min on ice and sonicated. The debris was removed by centrifugation at 12,000 *g* for 20 min at 4 °C, and the clear lysate was diluted with 10 mL of wash buffer (20 mM Tris-HCl pH 7.5, 500 mM NaCl, 5 mM imidazole, 7 mM β-mercaptoethanol, 5% (v/v) glycerol, MQ) and incubated for 1 h at 4 °C with 400 µL of nickel-nitrilotriacetic acid (Ni-NTA) resin suspension (Ni Sepharose 6 fast flow, catalog no. 17531804; GE Healthcare) under agitation. The resin was washed 3 times with 10 ml of wash buffer for 5 min at 4 °C under agitation. The protein of interest was then eluted with 1,5 mL of elution buffer (20 mM Tris-HCl pH 7,5, 500 mM NaCl, 200 mM imidazole, 7 mM β-mercaptoethanol, 5% (v/v) glycerol, MQ) for

1 h at 4 °C, dialyzed against dialysis buffer (20 mM Tris-HCl pH 7.5, 200 mM NaCl, 7 mM β-mercaptoethanol, 5% (v/v) glycerol, MQ) overnight (16h) at 4°C and diluted 1:1 (v/v) by glycerol. The purity and concentration of proteins were verified; all proteins had a single major band of corresponding size. The purified samples were stored at −20 °C.

Klenow fragment test

5 µL of the 1st solution (0.5 µL 10x buffer for the Klenov fragment, 0.5 µL dNTP (2mM), 1 µL mixture of primers (1 µM each, GGTTATAATGAATTTTGCTTATTAACGATAGAATTCTATCACATTCTTGATTCTTAAC TACGACCACAATTACG and AAGTATAAGGAGGAAAACATATGTGTCATCGTGATTATCGTTATTATAATGGTTATG GTGGTTGTGGTCGTAATTGTGGTCGTAGTTAA), 3 µL MQ) per point were heated to 95 °C and then slowly passively cooling it to room temperature. Then 2nd solution (0.5 µL 10x buffer for the Klenov fragment, 2.25 µL MQ, 0.25 µL Klenov fragment (5U), 2 µL test sample per point) were added to the 1st solution and incubated at 37 °C for 30 minutes. Control points was added: a point without an enzyme, a point with MQ instead of test sample and a point with a known intercalator (ethidium bromide, 100 µg/mL). Obtained samples were analyzed by 10% urea-PAGE. Gels were scanned using a Typhoon FLA 9500 Biomolecular Imager (GE Healthcare), and graphical illustrations were created using Inkscape (version 1.4.2.).

Topo1-mediated DNA relaxation

Reaction mix (3,3 µl 3X Gyrase buffer (105 mM Tris-HCl pH 7.5, 18 mM $MgCl_2$, 5.4 mM spermidine, 72 mM KCl, 15 mM dithiotreitol, 1.08 mg/mL BSA, 19.5% (v/v) glycerol, MQ), 1 µL supercoiled pHot (200 ng/µl), 0,5 µL TopA, 4,2 µL MQ, 1 µL test sample per point) were incubated at 37 °C for 60 minutes. Control points was added: a point without an enzyme and a point with MQ instead of test sample. After that, 1:4 (v/v) 5× stop

solution (5% (v/v) SDS, 25% (v/v) glycerol, 0.25 mg/mL of bromophenol blue) was added. Obtained samples were analyzed by 1% agarose gel. Gels were scanned using a Typhoon FLA 9500 Biomolecular Imager (GE Healthcare), and graphical illustrations were created using Inkscape (version 1.4.2.).

Topo4-mediated DNA cleavage

Reaction mix (2 µL 5X Topo buffer (200 mM Tris-HCl pH=7.5, 500 mM potassium glutamate, 50 mM magnesium acetate, 50 mM DTT, 250 µg/mL BSA, MQ), 1 µl supercoiled pHot (200 ng/µL), 0,5 µL ParC, 0,5 µL ParE, 5 µL MQ, 1 µL test sample per point) were incubated at 37 °C for 60 minutes. Control points was added: a point without an enzyme and a point with mQ water instead of test sample. Reaction was then treated with 0.45% (vol/vol) SDS and 0.45 mg/mL of proteinase K at 37 °C for 30 min. After treatment, 1:4 (v/v) 5× stop solution was added. Obtained samples were analyzed by 1% agarose gel. Gels were scanned using a Typhoon FLA 9500 Biomolecular Imager (GE Healthcare), and graphical illustrations were created using Inkscape (version 1.4.2.).

Gyrase-mediated DNA cleavage

Reaction mix (3,3 µL 3X Gyrase buffer, 1 µL supercoiled pHot (200 ng/µL), 0,1 µL GyrA, 0,9 µL GyrB, 3,7 µL MQ, 1 µL test sample per point) were incubated at 37 °C for 60 minutes. Control points was added: a point without an enzyme and a point with MQ instead of test sample. Reaction was then treated with 0.45% (vol/vol) SDS and 0.45 mg/mL of proteinase K at 37 °C for 30 min. After treatment, 1:4 (v/v) 5× stop solution was added. Obtained samples were analyzed by 1% agarose gel. Gels were scanned using a Typhoon FLA 9500 Biomolecular Imager (GE Healthcare), and graphical illustrations were created using Inkscape (version 1.4.2.).

Topo4-mediated DNA decatenation

Reaction mix (2 µL 5X Topo, 1 µL kDNA (100 ng/µL, TopoGEN), 0,5 µL ParC, 0,5 µL ParE, 1 µL ATP (25 mM), 4 µL MQ, 1 µL test sample per point) were incubated at 37 °C for 60 minutes. Control points was added: a point without an enzyme and a point with MQ instead of test sample. Reaction was then treated with 0.45% (vol/vol) SDS and 0.45 mg/mL of proteinase K at 37 °C for 30 min. After treatment, 1:4 (v/v) 5× stop solution was added. Obtained samples were analyzed by 1% agarose gel. Gels were scanned using a Typhoon FLA 9500 Biomolecular Imager (GE Healthcare), and graphical illustrations were created using Inkscape (version 1.4.2.).

Gyrase-mediated DNA supercoiling

Relaxed plasmid was obtained by Topo1-mediated relaxation: relaxed mix (1 µL 3X Gyrase buffer, 1 µL supercoiled pHot (200 ng/µL), 0,5 µL TopA, 0,5 µL MQ per point) were incubated at 37 °C for 30 minutes, and then Topo1 was deactivated by high temperature (95 °C, 5 minutes). Then reaction mix (3 µL relaxed mix, 2,3 µL 3X Gyrase buffer, 0,1 µL GyrA, 0,9 µL GyrB, 1 µL ATP (25 mM), 1,7 µL MQ, 1 µL test sample per point) were incubated at 37 °C for 60 minutes. Control points was added: a point without an enzyme and a point with MQ instead of test sample. Reaction was then treated with 0.45% (vol/vol) SDS and 0.45 mg/mL of proteinase K at 37 °C for 30 min. After treatment, 1:4 (v/v) 5× stop solution was added. Obtained samples were analyzed by 1% agarose gel. Gels were scanned using a Typhoon FLA 9500 Biomolecular Imager (GE Healthcare), and graphical illustrations were created using Inkscape (version 1.4.2.).

References

1. Volkova Y.A.; Averina E.B.; Vasilenko D.A.; Sedenkova K.N.; Grishin Y.K.; Bruheim P.; Kuznetsova T.S.; Zefirov N.S. *J. Org. Chem.*, **2019**, *84*, 3192-3200.

2. Vasilenko D. A. Dronov S. E., Parfiryeu D. U., Sadovnikov K. S., Sedenkova K. N., Grishin Y. K., Rybakov V. B., Kuznetsova T. S., Averina E. B. *Org. Biomol. Chem*., **2021**, *19*, 6447-6454.

3. Osterman I.A.; Komarova E.S.; Shiryaev D.I.; Korniltsev I.A.; Khven I.M.; Lukyanov D.A.; Tashlitsky V.N.; Serebryakova M.V.; Efremenkova O.V.; Ivanenkov Y.A.; Bogdanov A.A.; Sergiev P.V.; Dontsova O.A. *Antimicrob. Agents Ch.*, **2016**, *60*, 7481-7489.

4. Osterman I.A.; Wieland M.; Maviza T.P.; Lashkevich K.A.; Lukianov D.A.; Komarova E.S.; Zakalyukina Y.V.; Buschauer R.; Shiriaev D.I.; Leyn S.A.; Zlamal J.E.; Biryukov M.V.; Skvortsov D.A.; Tashlitsky V.N.; Polshakov V.I.; Cheng J.; Polikanov Y.S.; Bogdanov A.A.; Osterman A.L.; Dmitriev S.E.; Beckmann R.; Dontsova O.A.; Wilson D.N.; Sergiev P.V. *Nat. Chem. Biol.*, **2020**, *16*, 1071-1077.

5. Kitagawa M., Ara T., Arifuzzaman M., Ioka-Nakamichi T., Inamoto E., Toyonaga H., Mori H. *DNA Res.,* **2005**, 12, 291–299.

Methyl 5-(phenylthio)isoxazole-3-carboxylate **2a** (^{1}H NMR)

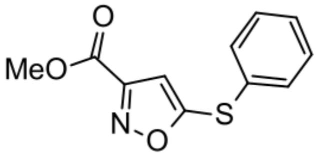

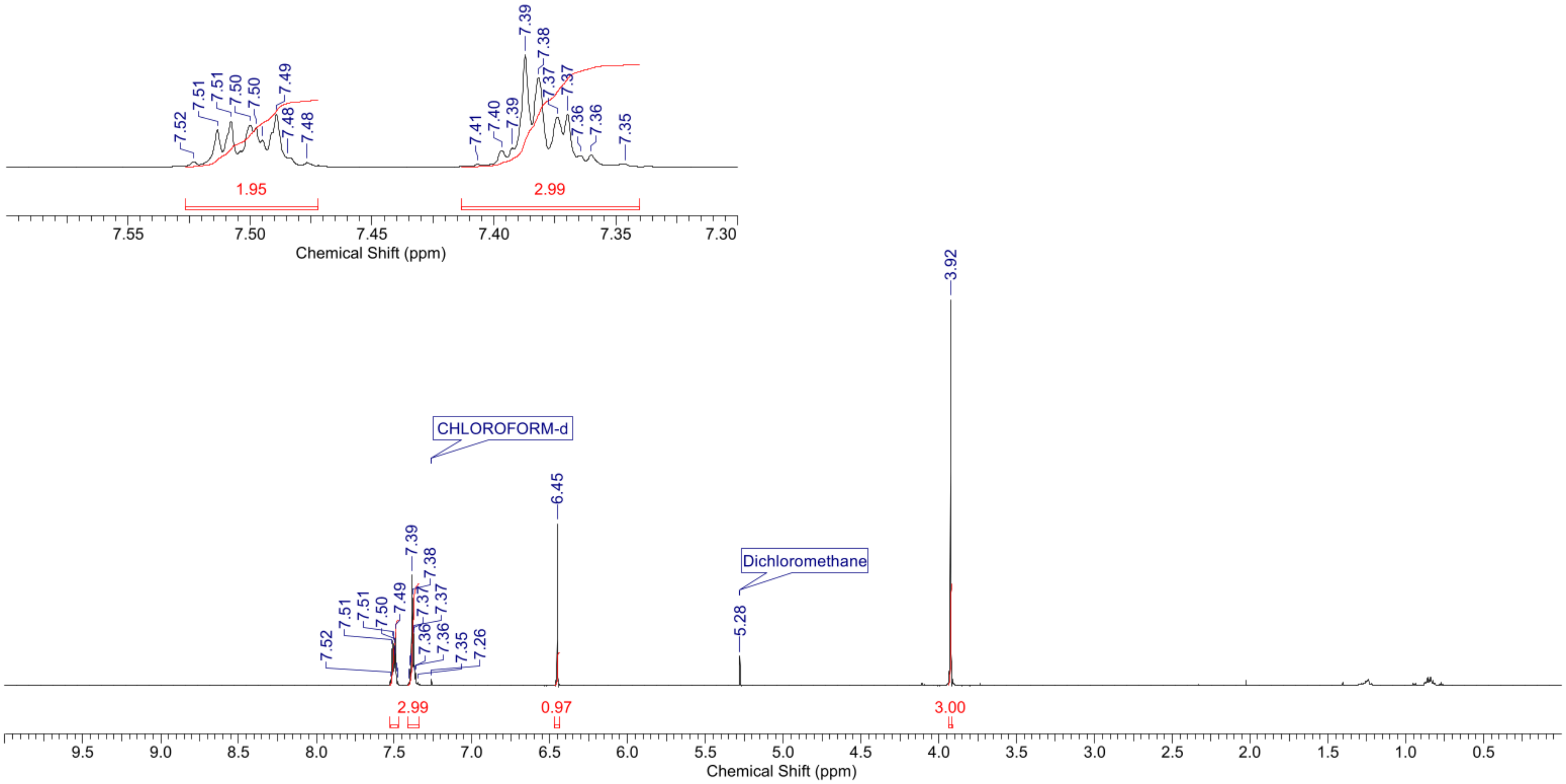

Methyl 5-(phenylthio)isoxazole-3-carboxylate **2a** (^{13}C NMR)

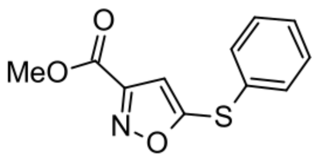

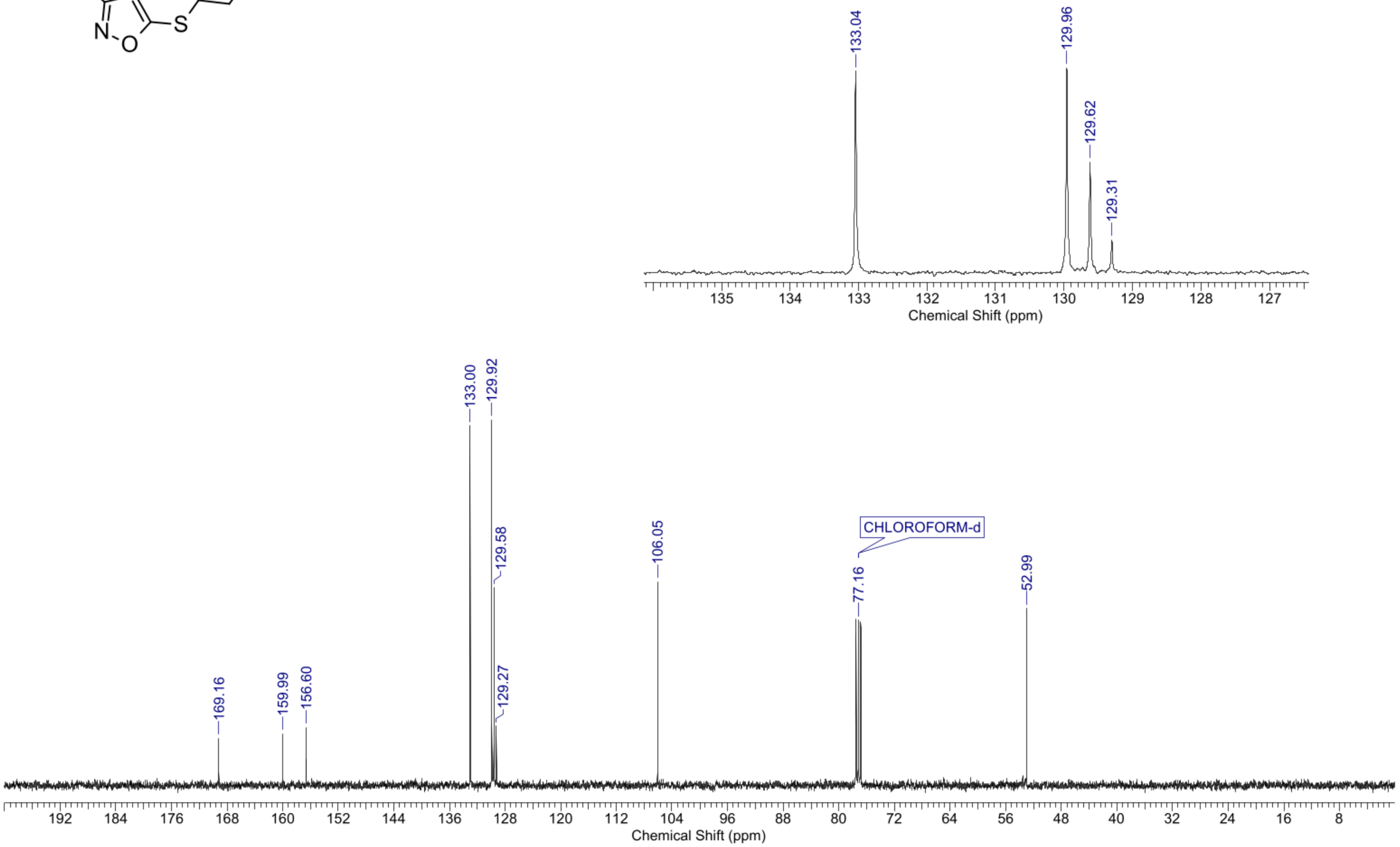

Tert-butyl 5-(phenylthio)isoxazole-3-carboxylate **2b** (^{1}H NMR)

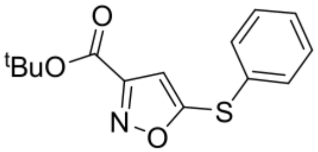

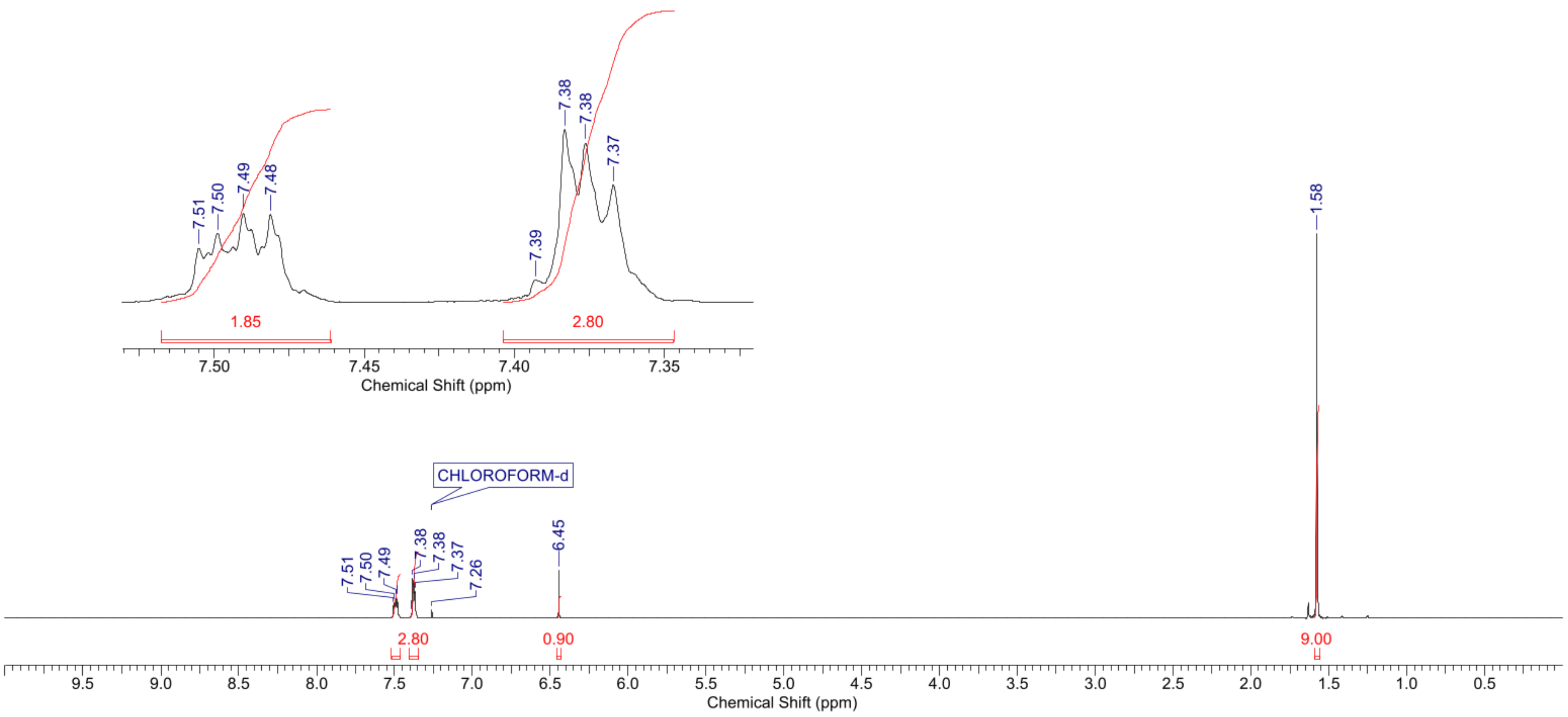

Tert-butyl 5-(phenylthio)isoxazole-3-carboxylate **2b** (^{13}C NMR)

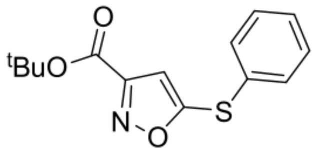

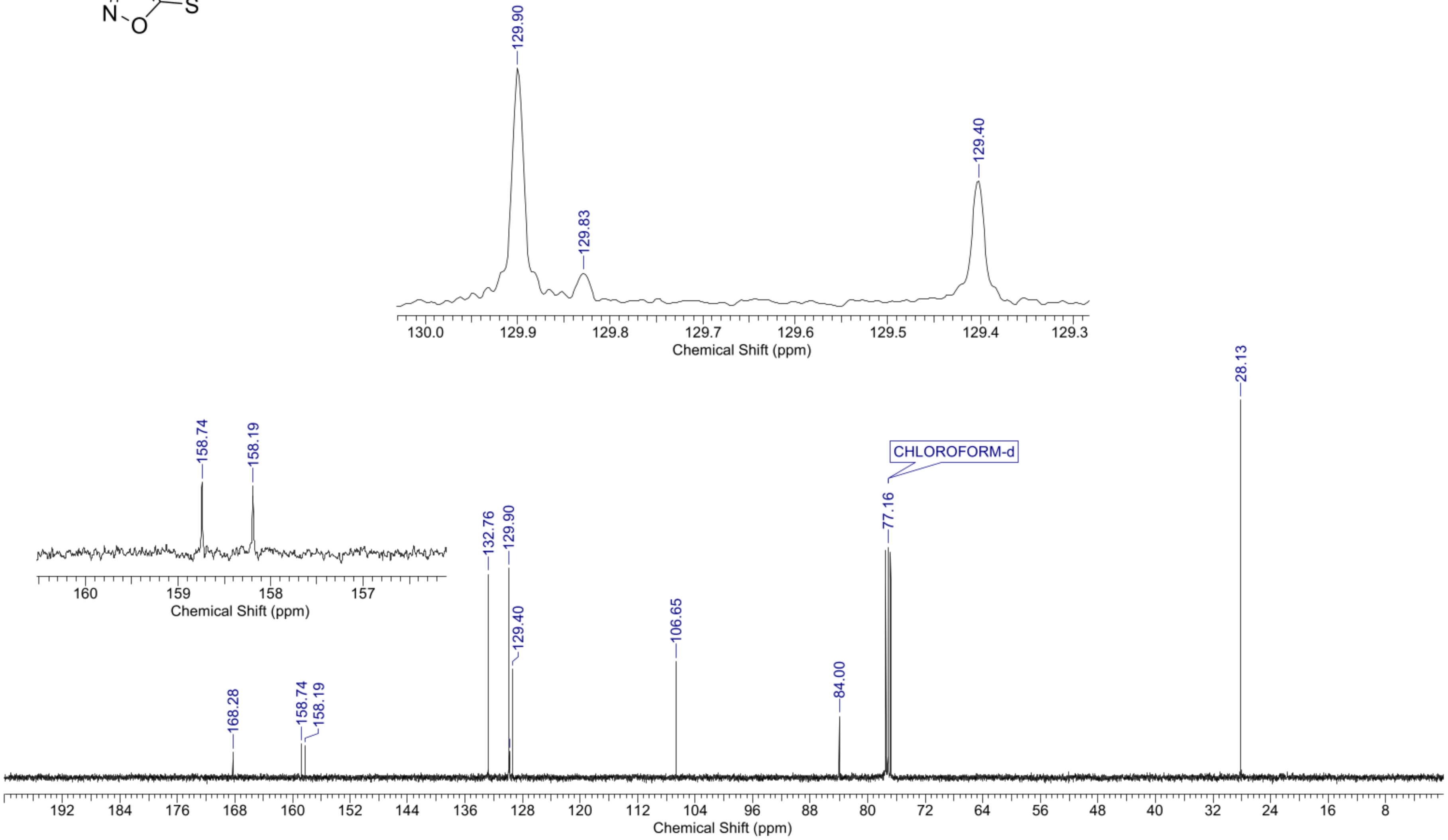

(Adamantan-1-yl)methyl 5-(phenylthio)isoxazole-3-carboxylate **2c** (^{1}H NMR)

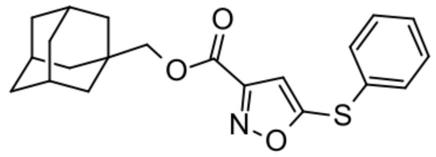

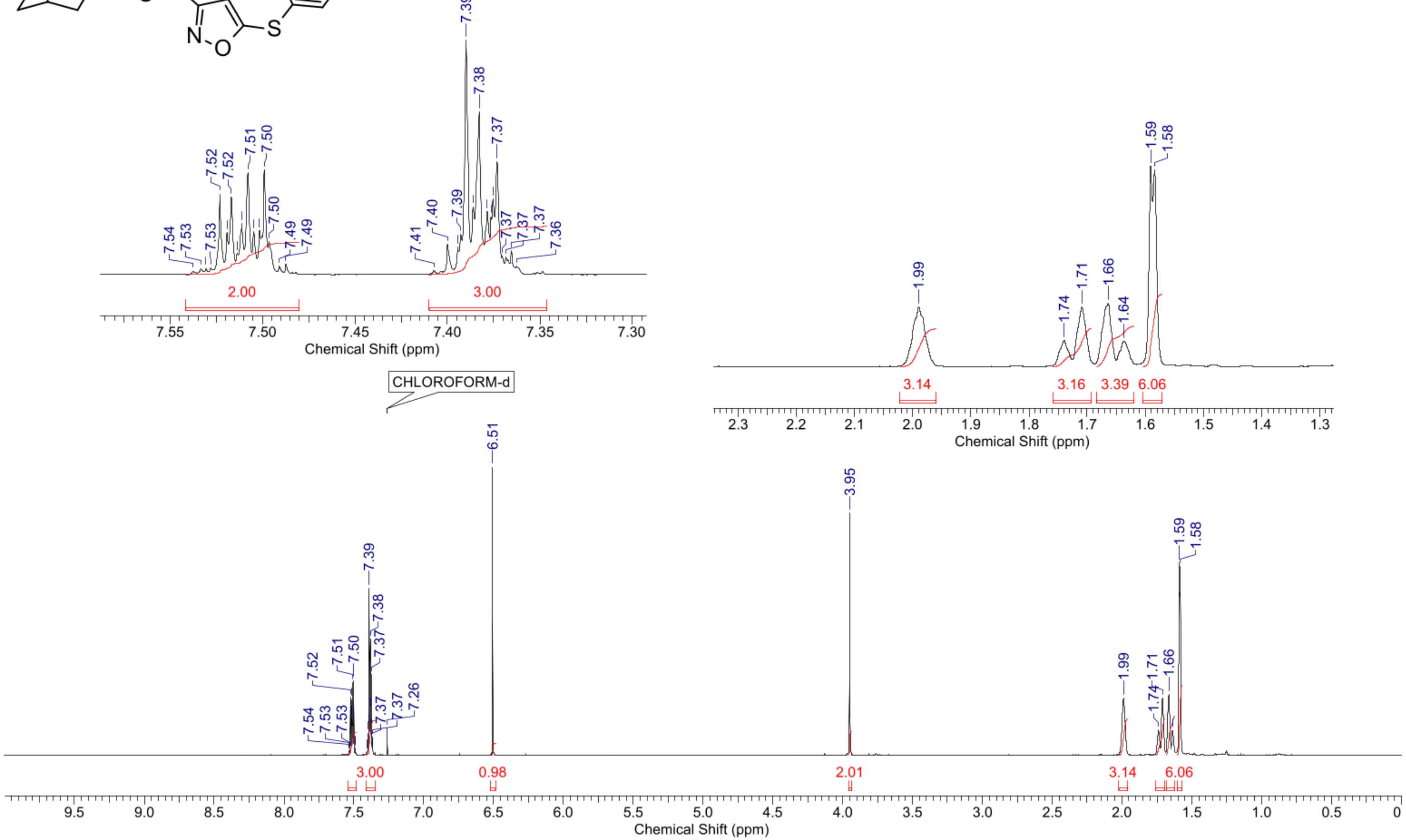

(Adamantan-1-yl)methyl 5-(phenylthio)isoxazole-3-carboxylate **2c** (^{13}C NMR)

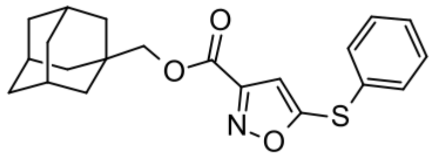

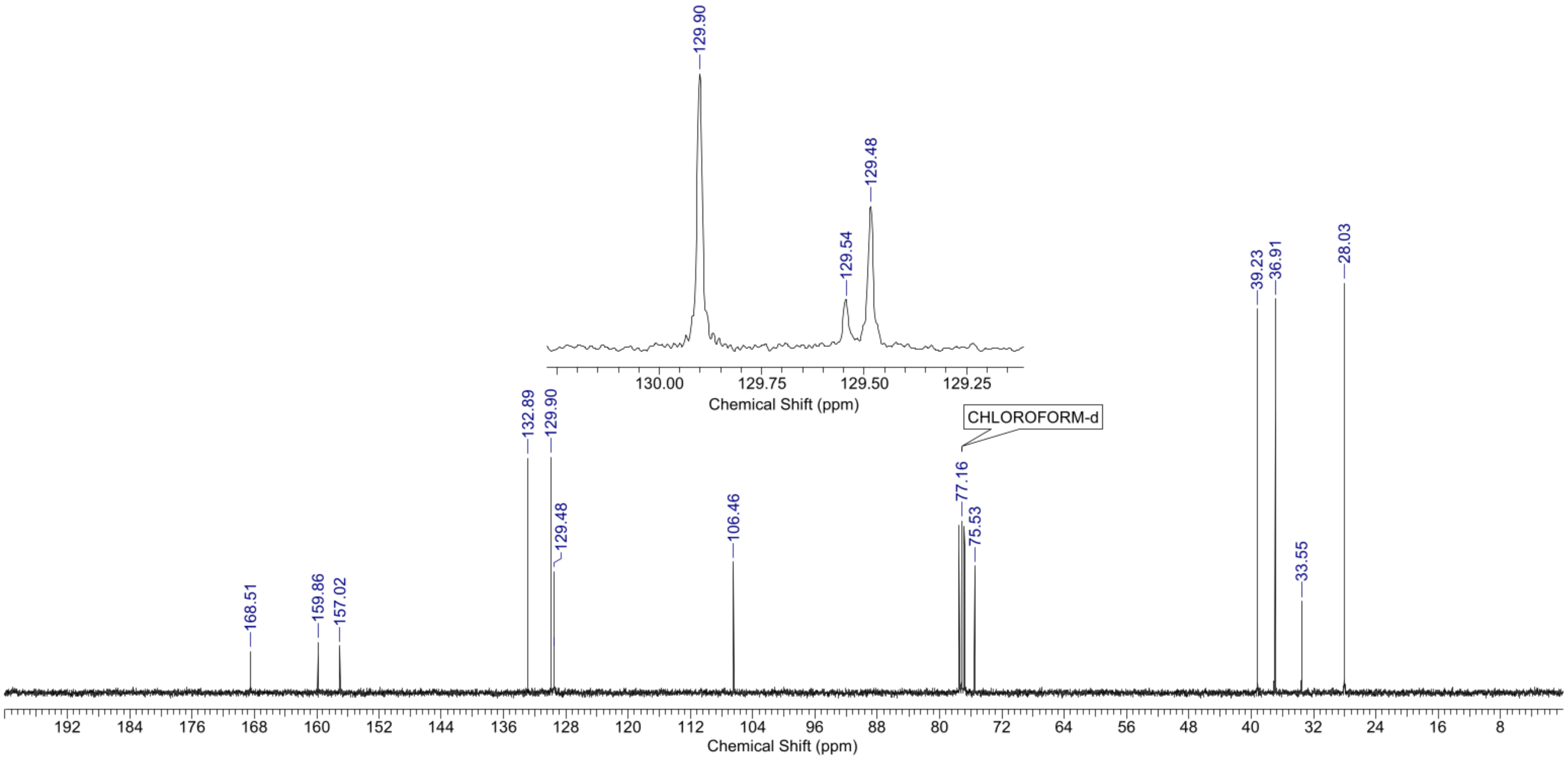

(Adamantan-1-yl)methyl 5-(phenylthio)isoxazole-3-carboxylate **2c** (APT)

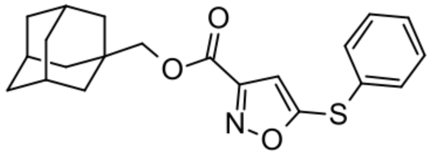

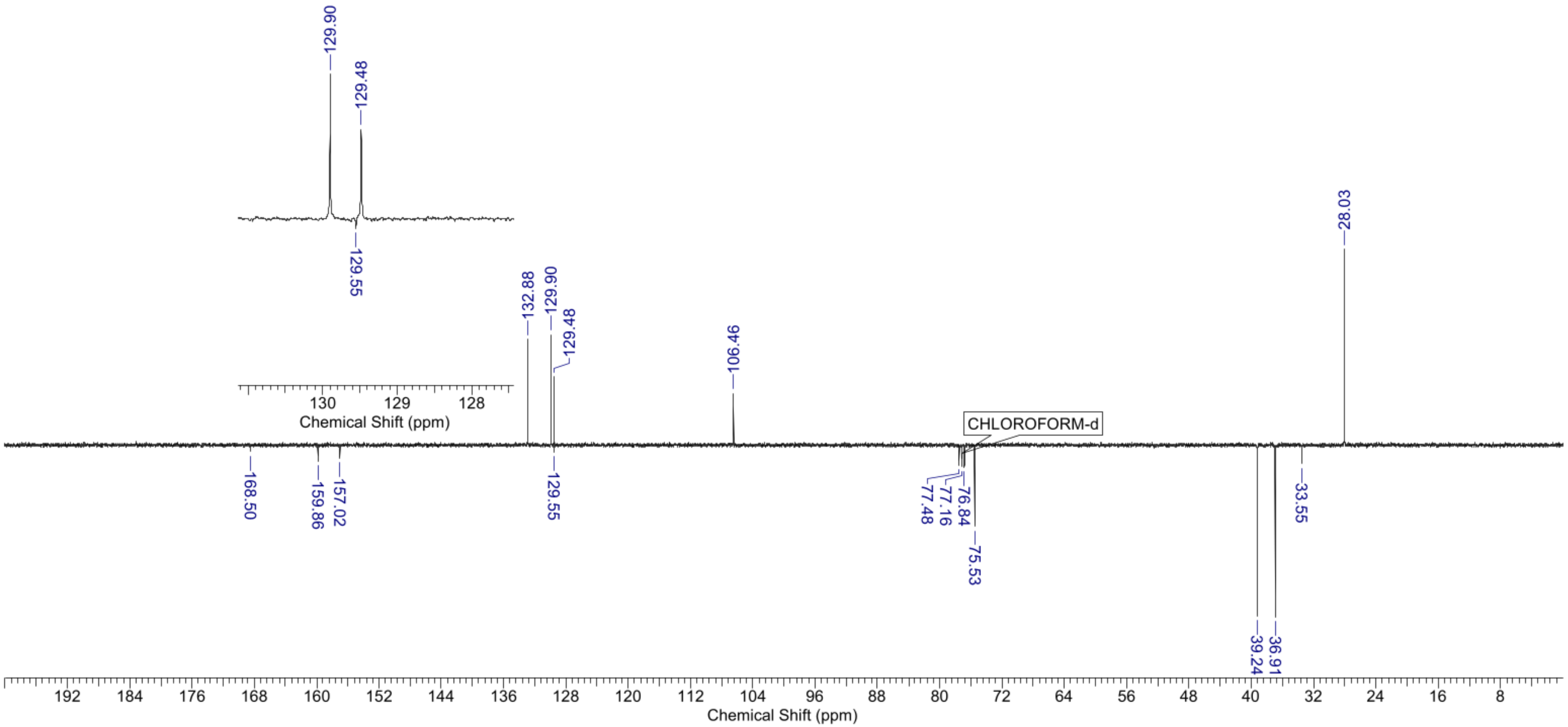

1-(5-(Phenylthio)isoxazol-3-yl)ethan-1-one **2d** (^{1}H NMR)

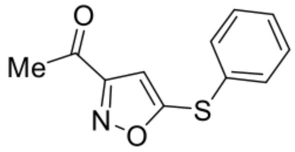

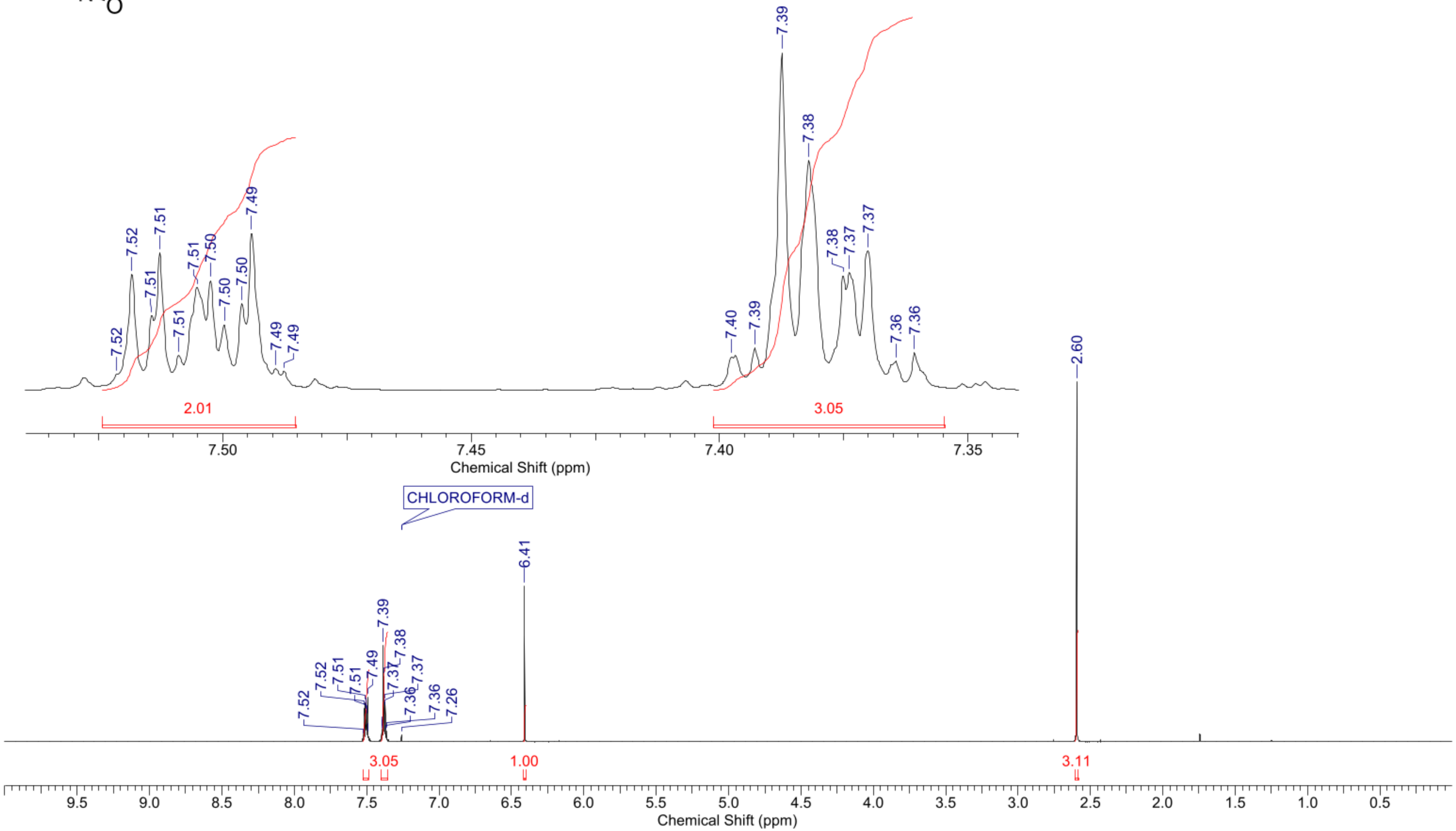

1-(5-(Phenylthio)isoxazol-3-yl)ethan-1-one **2d** (^{13}C NMR)

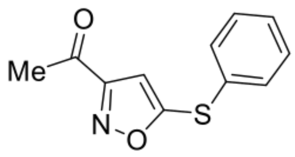

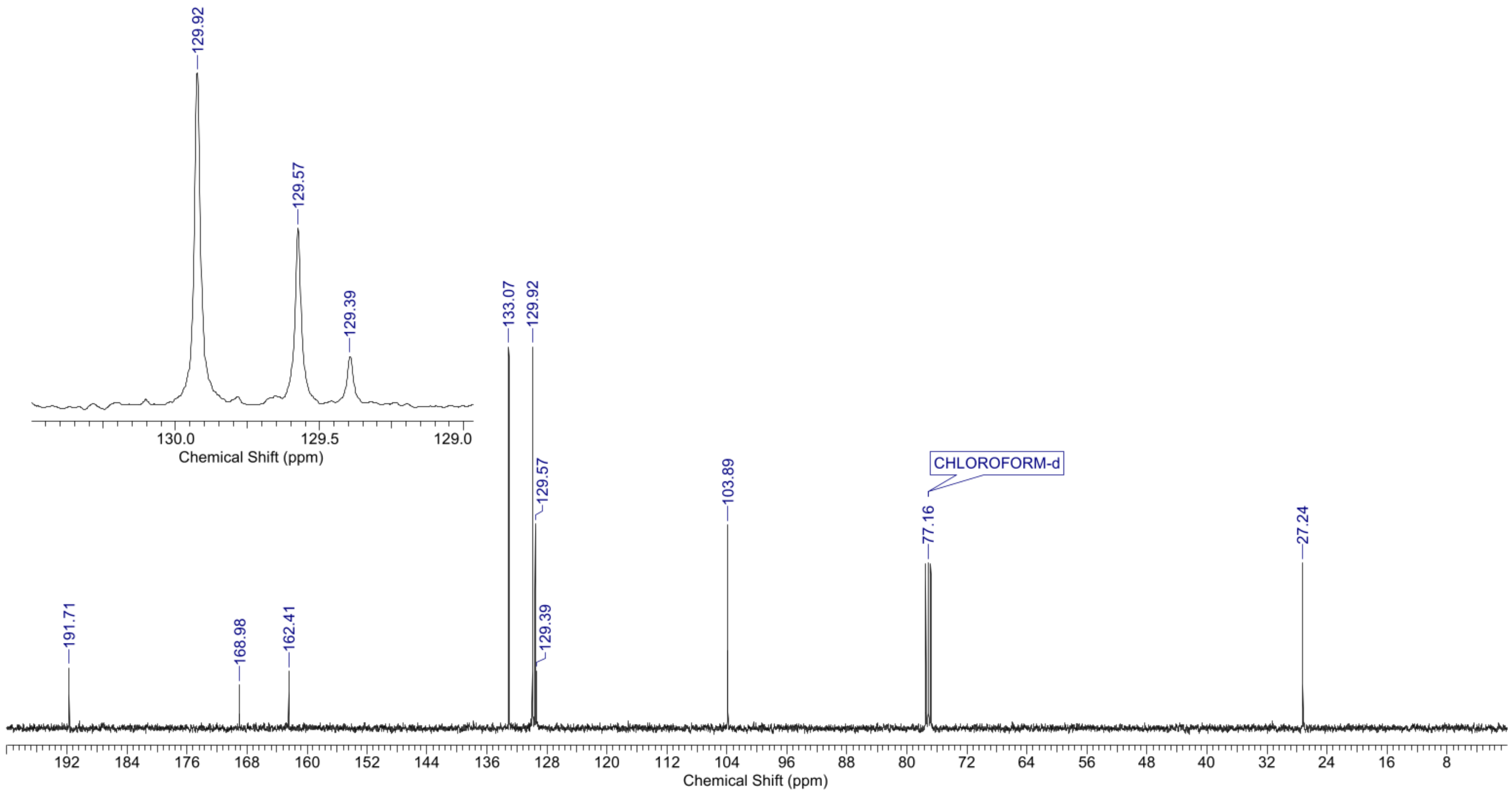

Phenyl(5-(phenylthio)isoxazol-3-yl)methanone **2e** (^{1}H NMR)

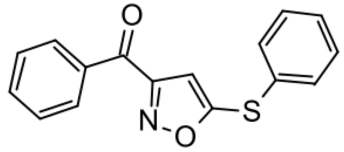

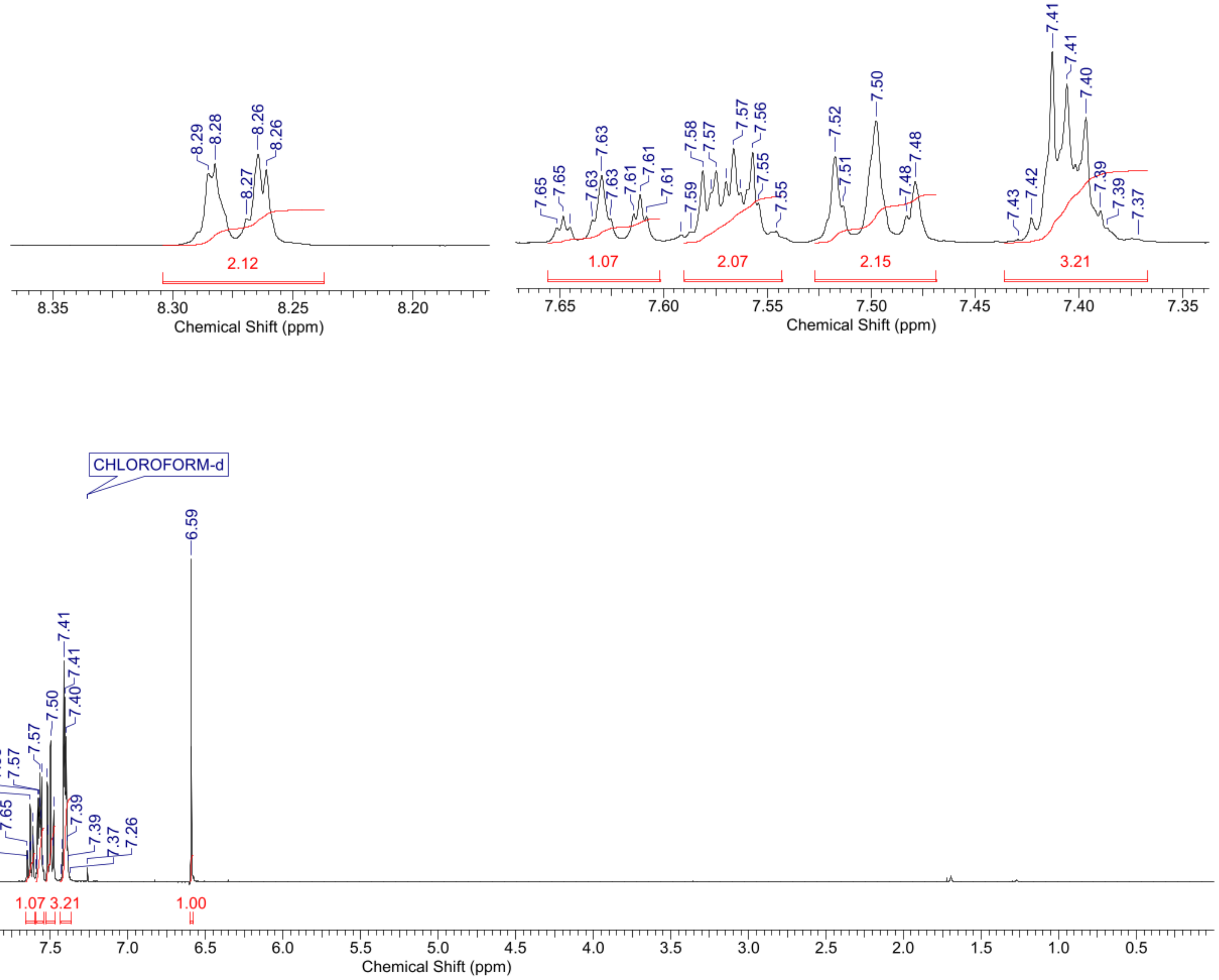

Phenyl(5-(phenylthio)isoxazol-3-yl)methanone **2e** (^{13}C NMR)

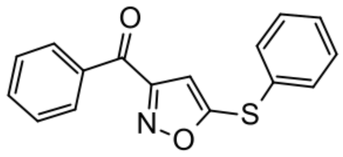

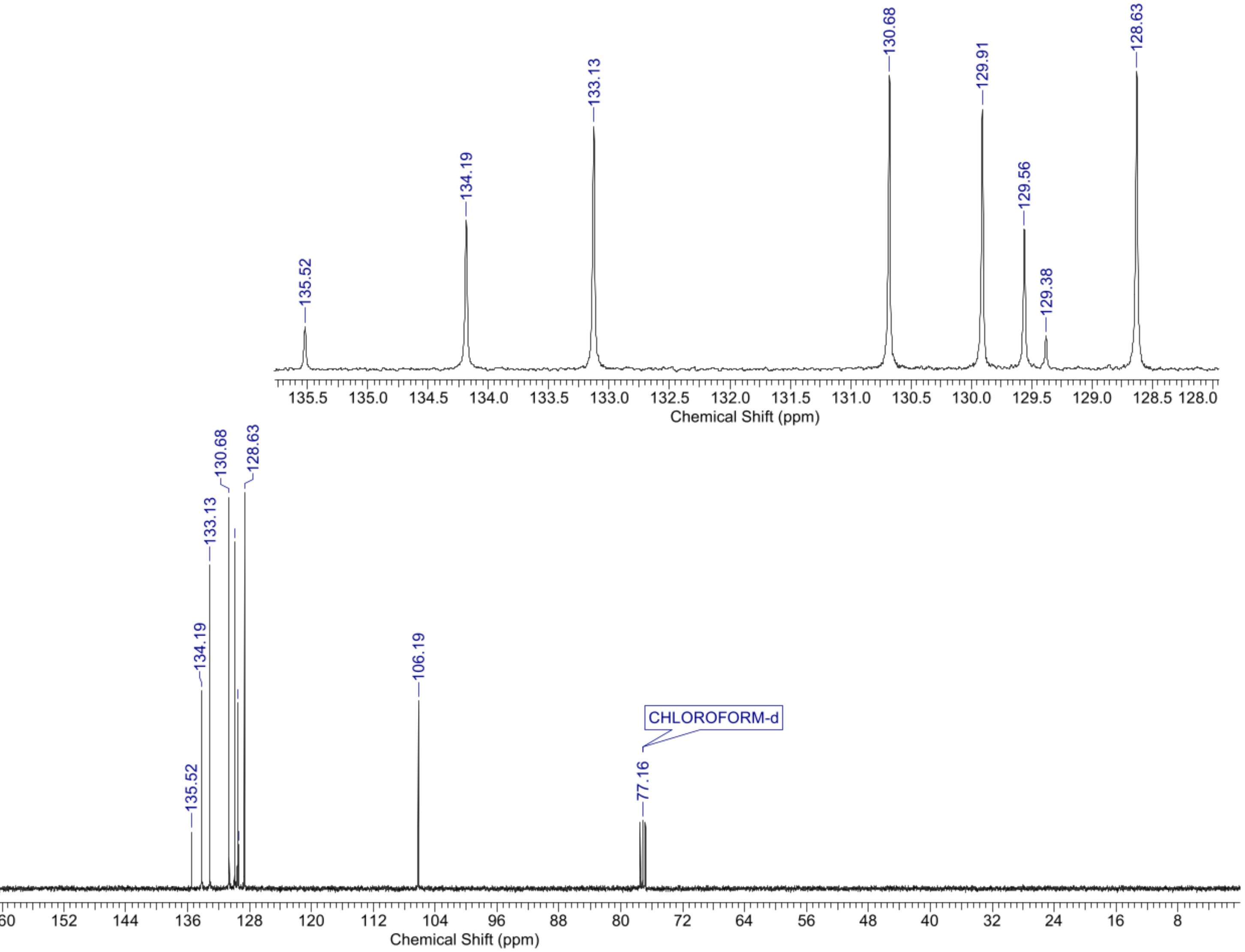

Diethyl (5-(phenylthio)isoxazole-3-carbonyl)phosphonate **2f** (^{1}H NMR)

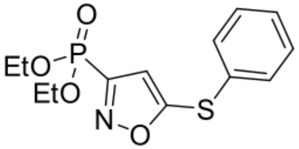

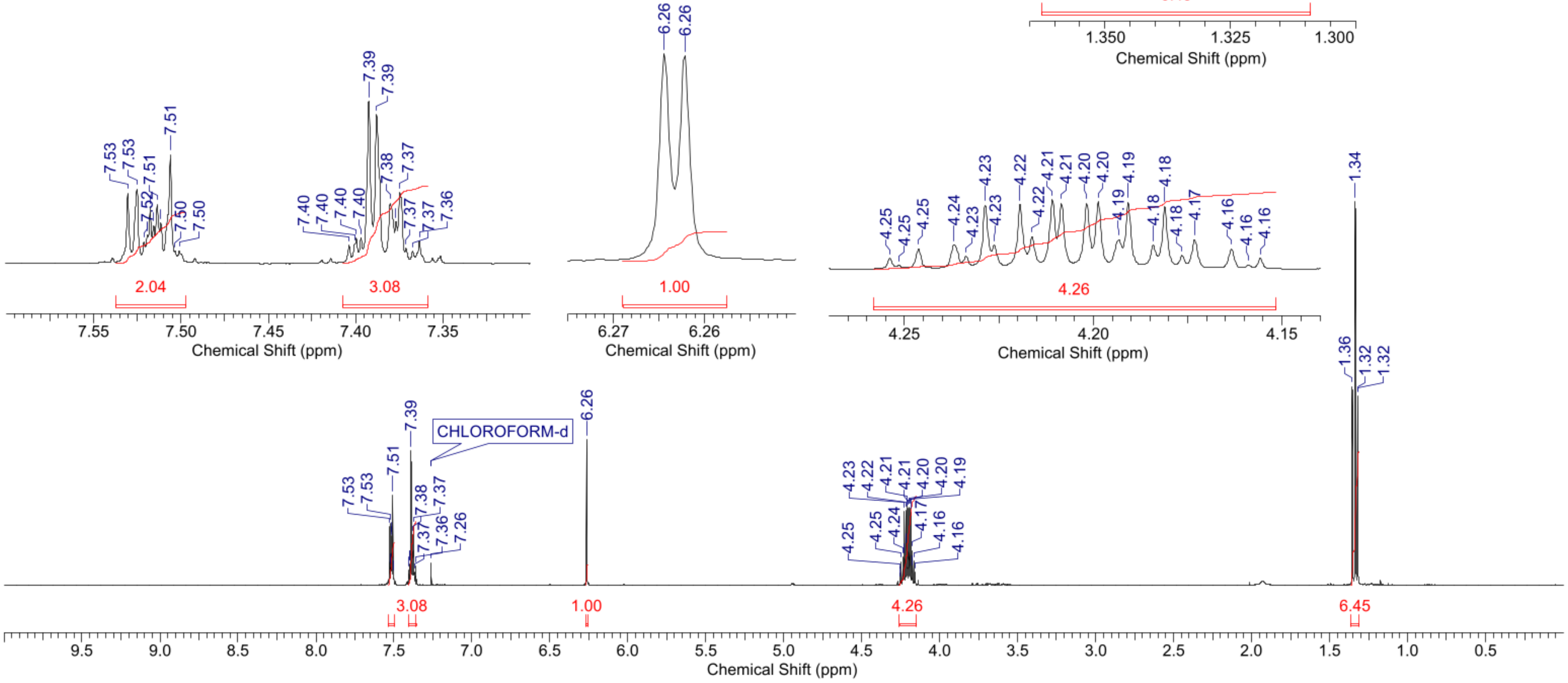

Diethyl (5-(phenylthio)isoxazole-3-carbonyl)phosphonate **2f** (^{13}C NMR)

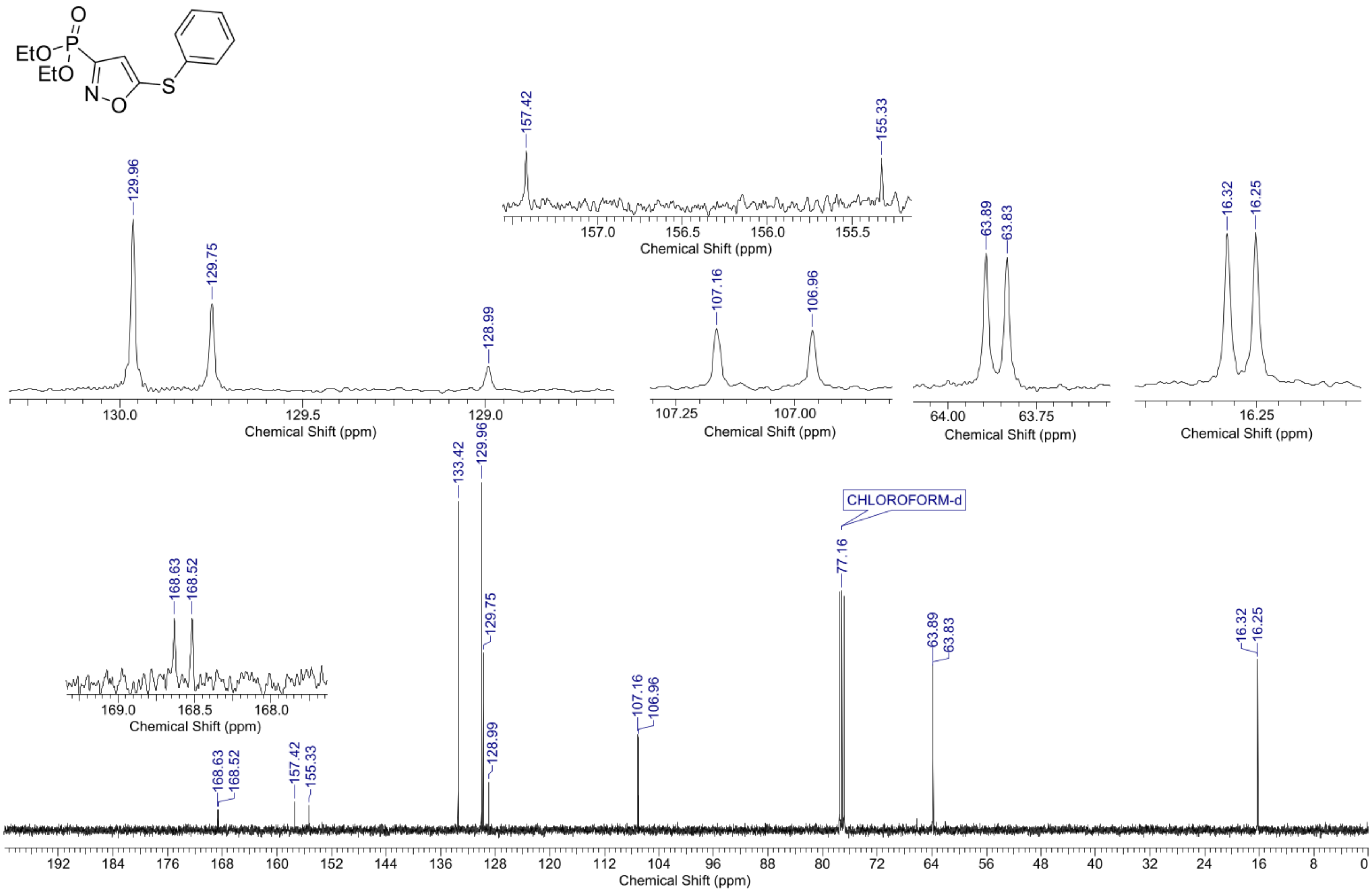

Diethyl (5-(phenylthio)isoxazole-3-carbonyl)phosphonate **2f** (^{31}P NMR)

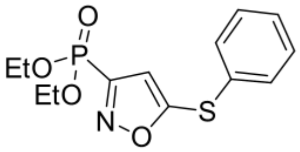

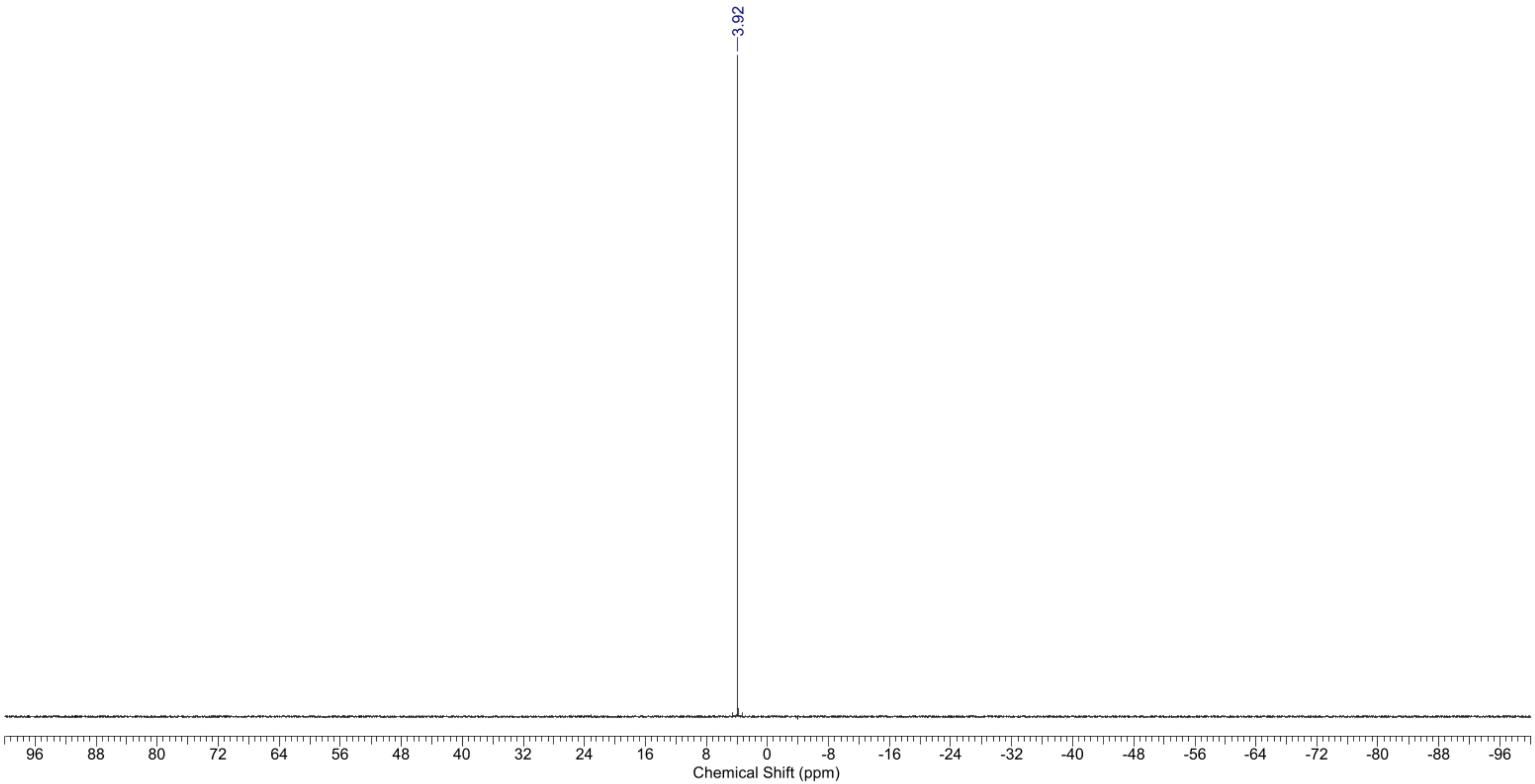

3-Nitro-5-(phenylthio)isoxazole **2g** (^{1}H NMR)

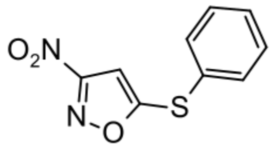

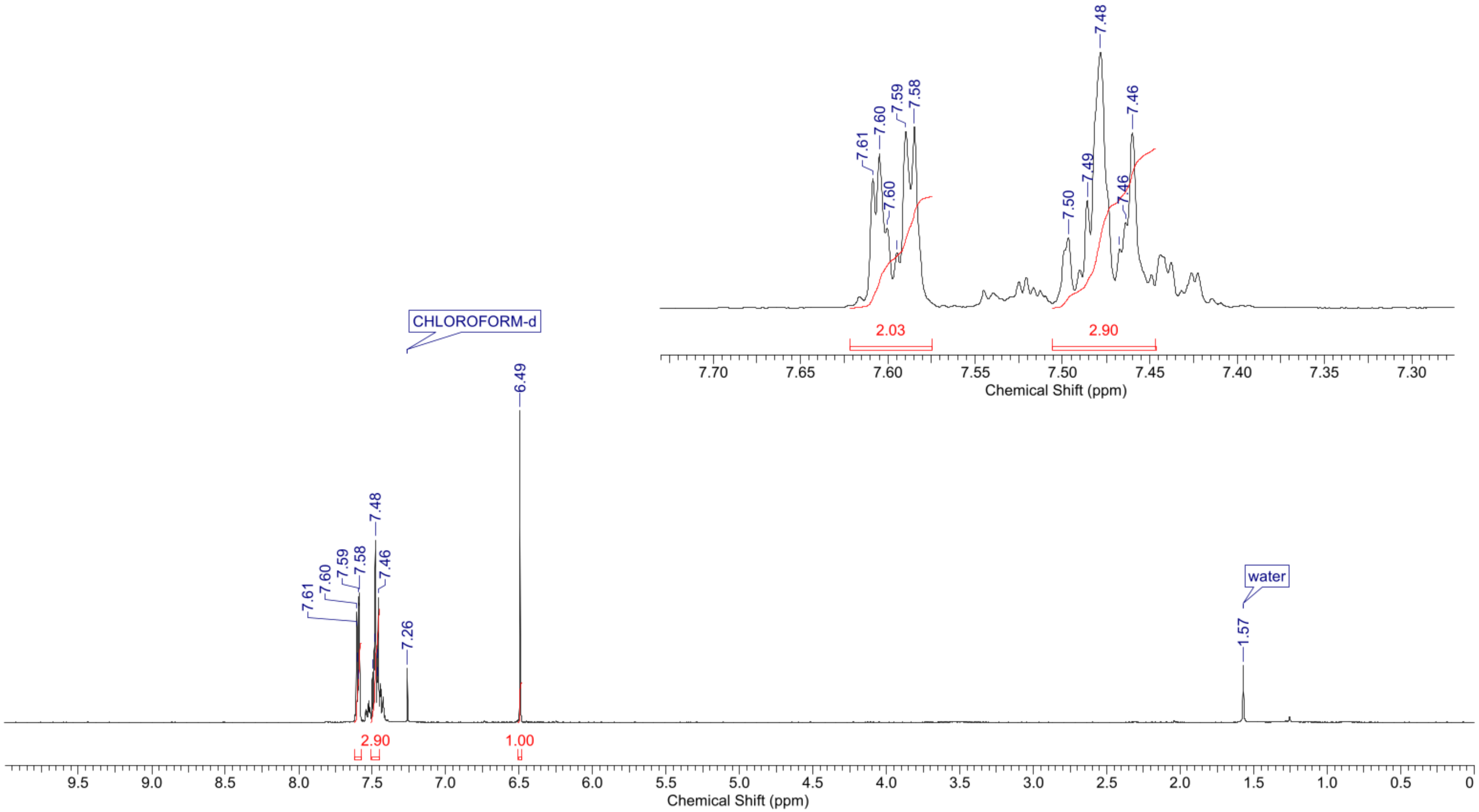

3-Nitro-5-(phenylthio)isoxazole **2g** (^{13}C NMR)

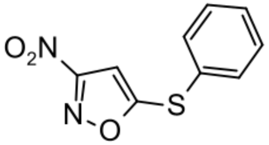

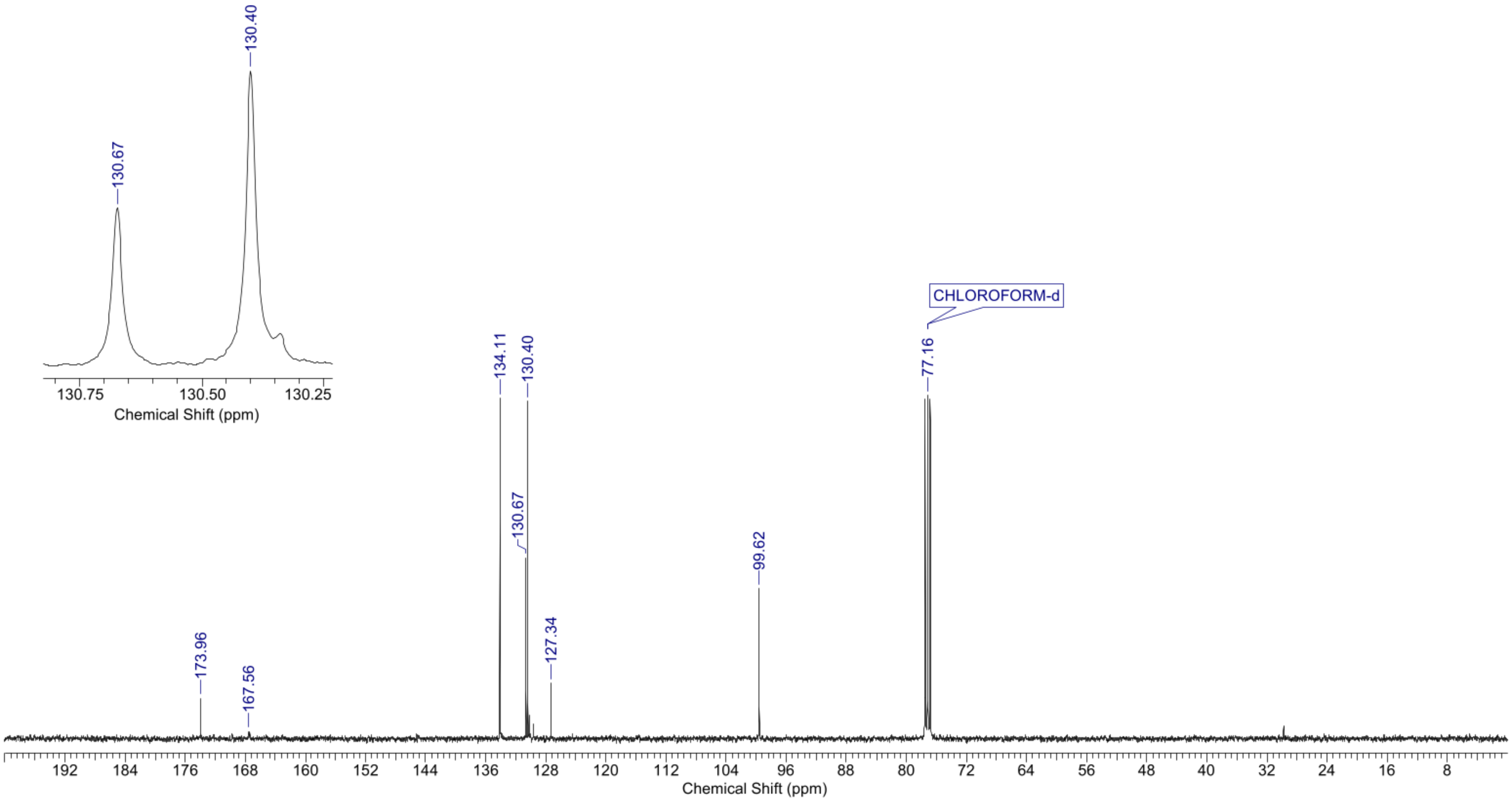

5-((4-Chlorophenyl)thio)-3-nitroisoxazole **2h** (^{1}H NMR)

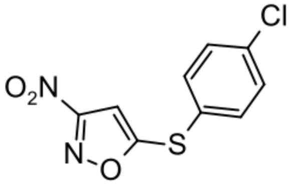

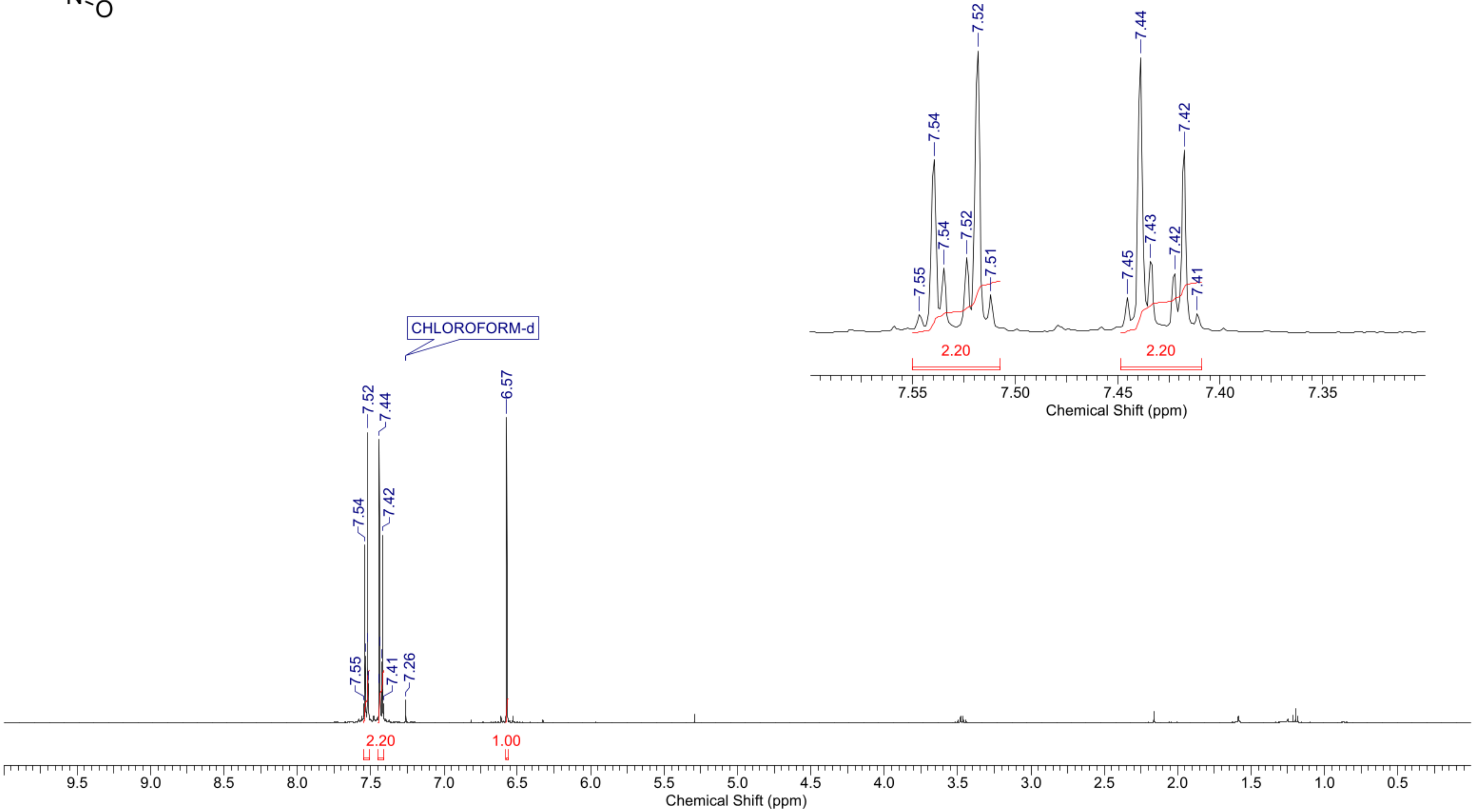

5-((4-Chlorophenyl)thio)-3-nitroisoxazole **2h** (^{13}C NMR)

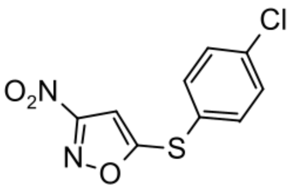

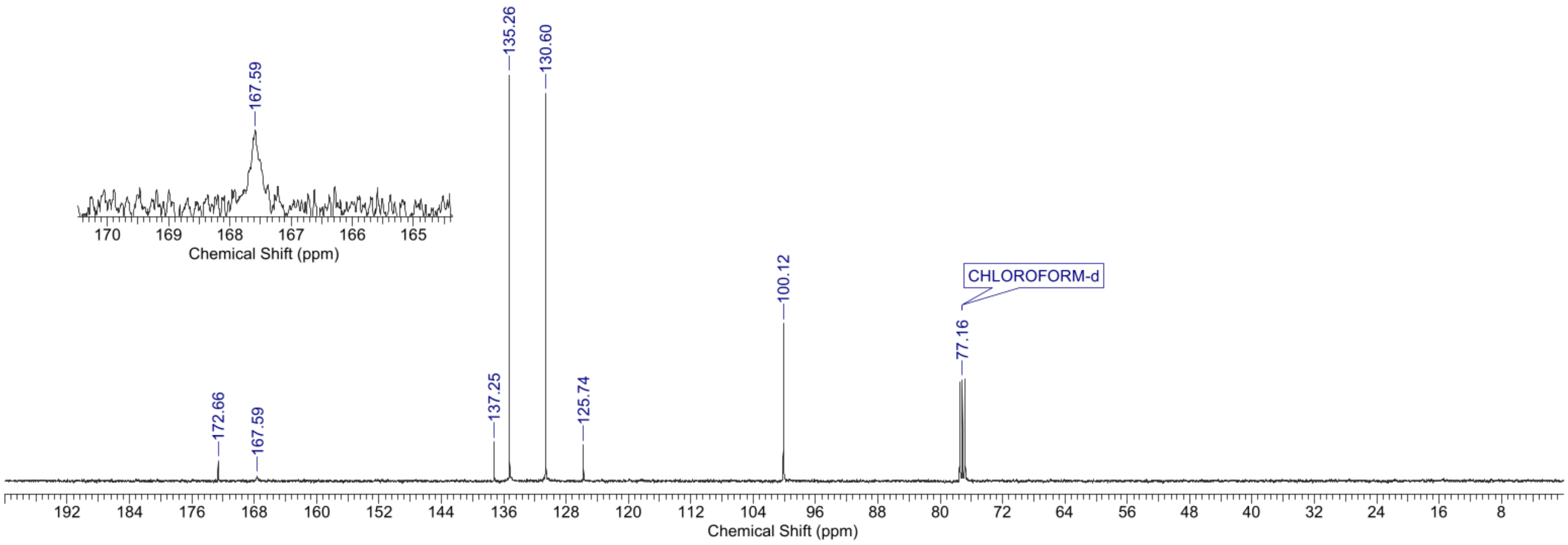

5-((4-Chlorophenyl)thio)-3-nitroisoxazole **2h** (HMBC)

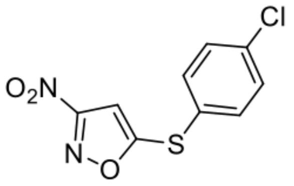

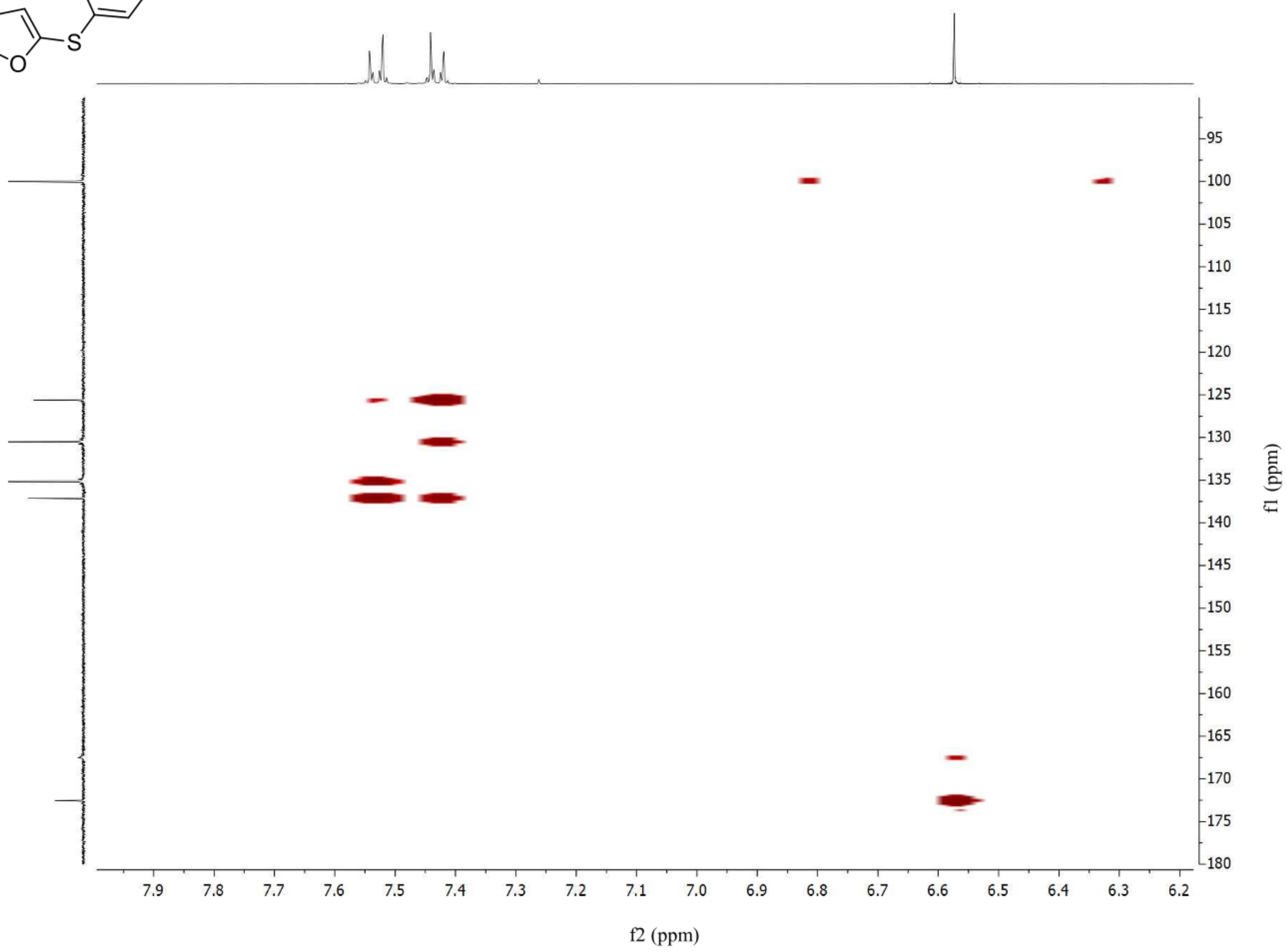

5-((4-Fluorophenyl)thio)-3-nitroisoxazole **2i** (^{1}H NMR)

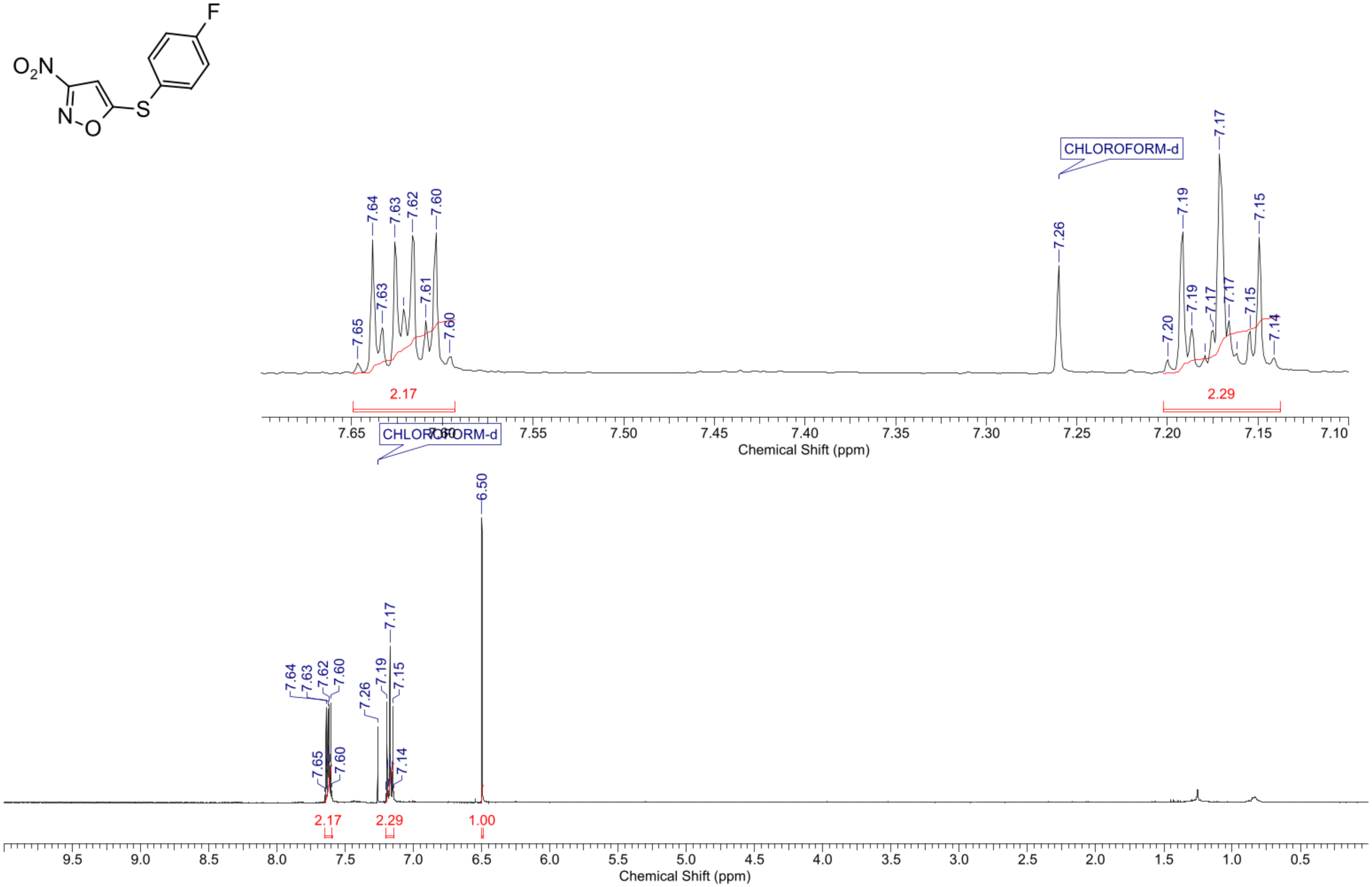

5-((4-Fluorophenyl)thio)-3-nitroisoxazole **2i** (^{13}C NMR)

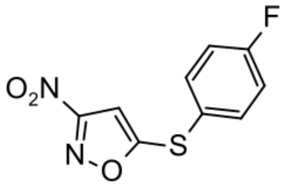

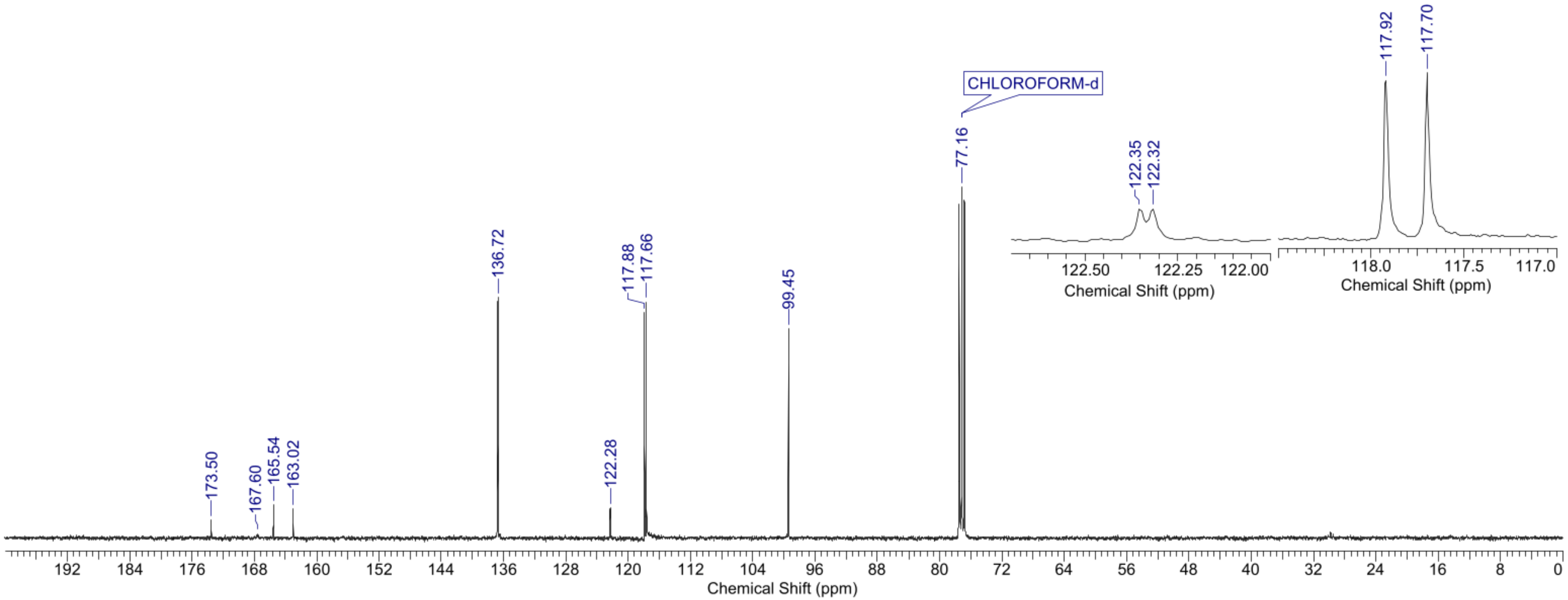

5-((4-Fluorophenyl)thio)-3-nitroisoxazole **2i** (^{19}F NMR)

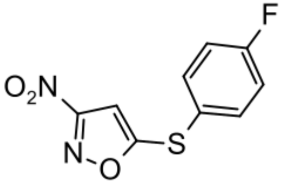

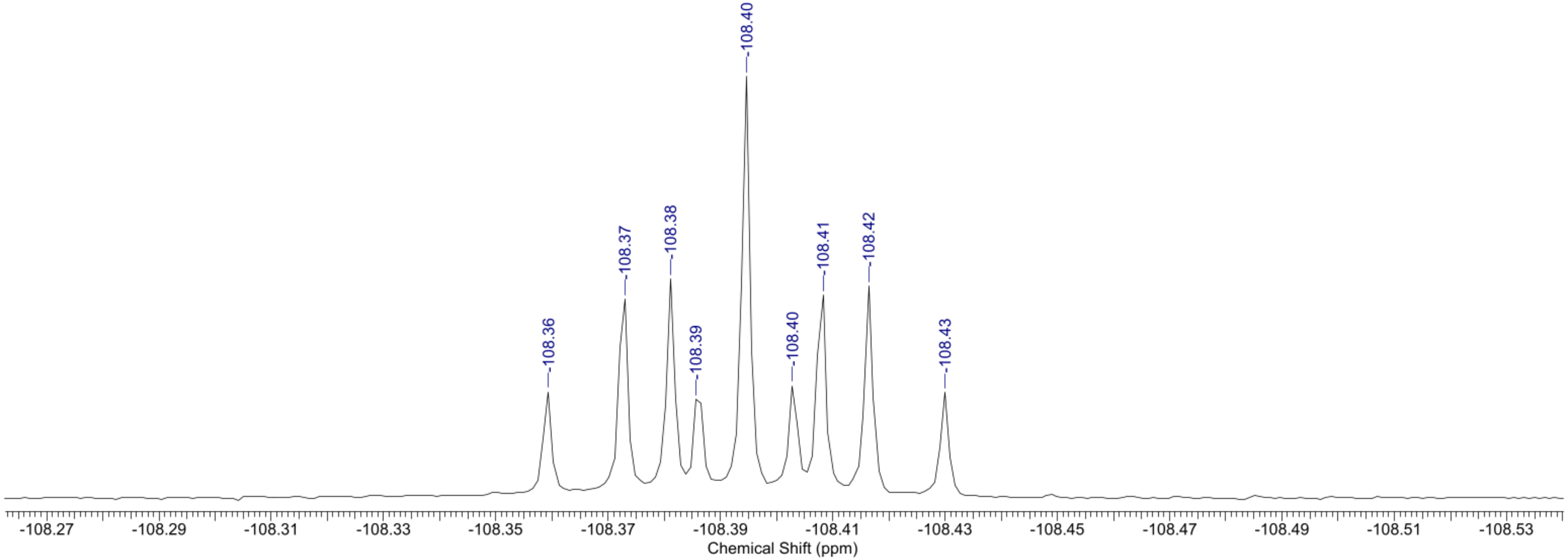

3-Nitro-5-(p-tolylthio)isoxazole **2j** (^{1}H NMR)

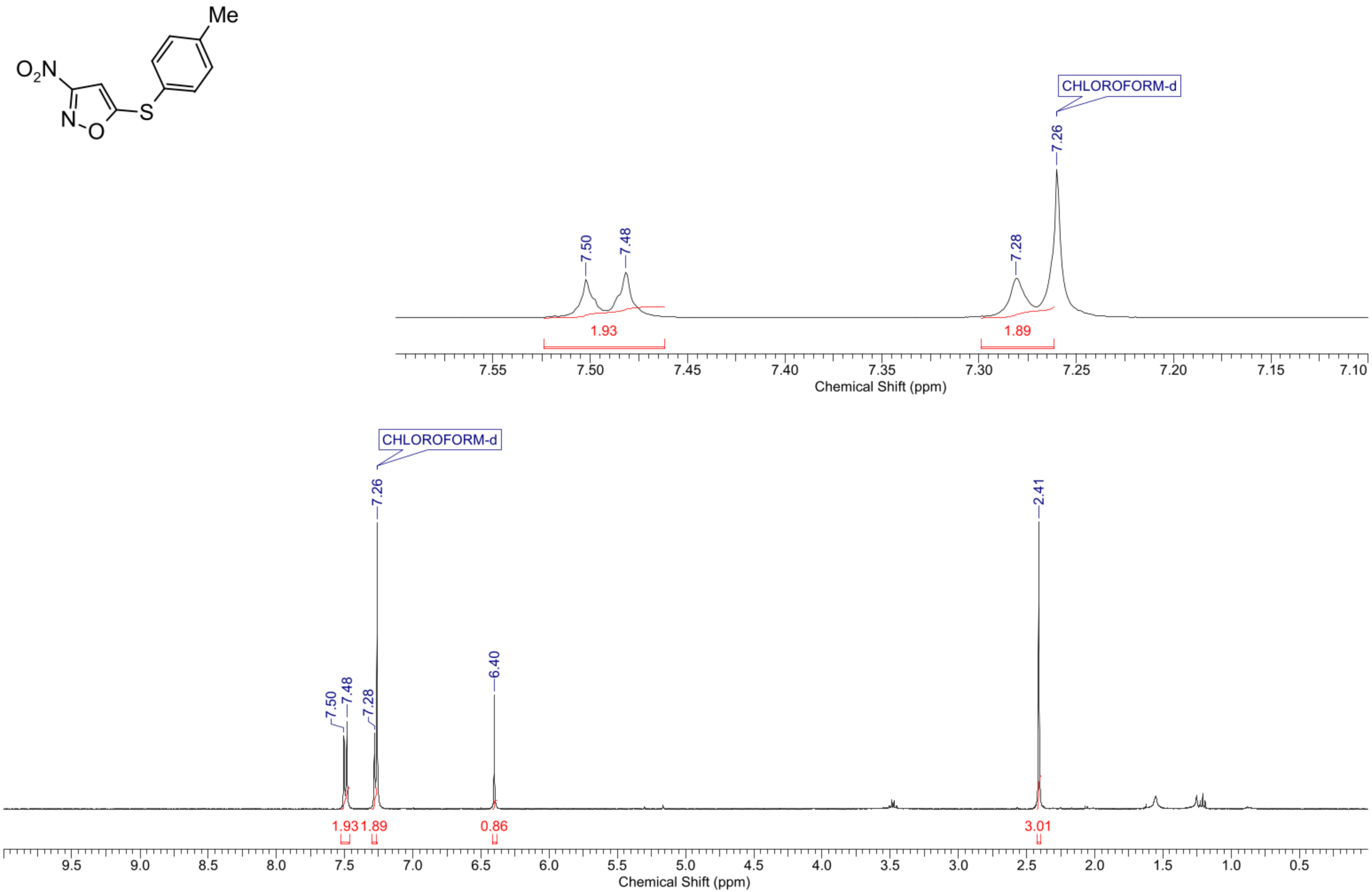

3-Nitro-5-(p-tolylthio)isoxazole **2j** (^{13}C NMR)

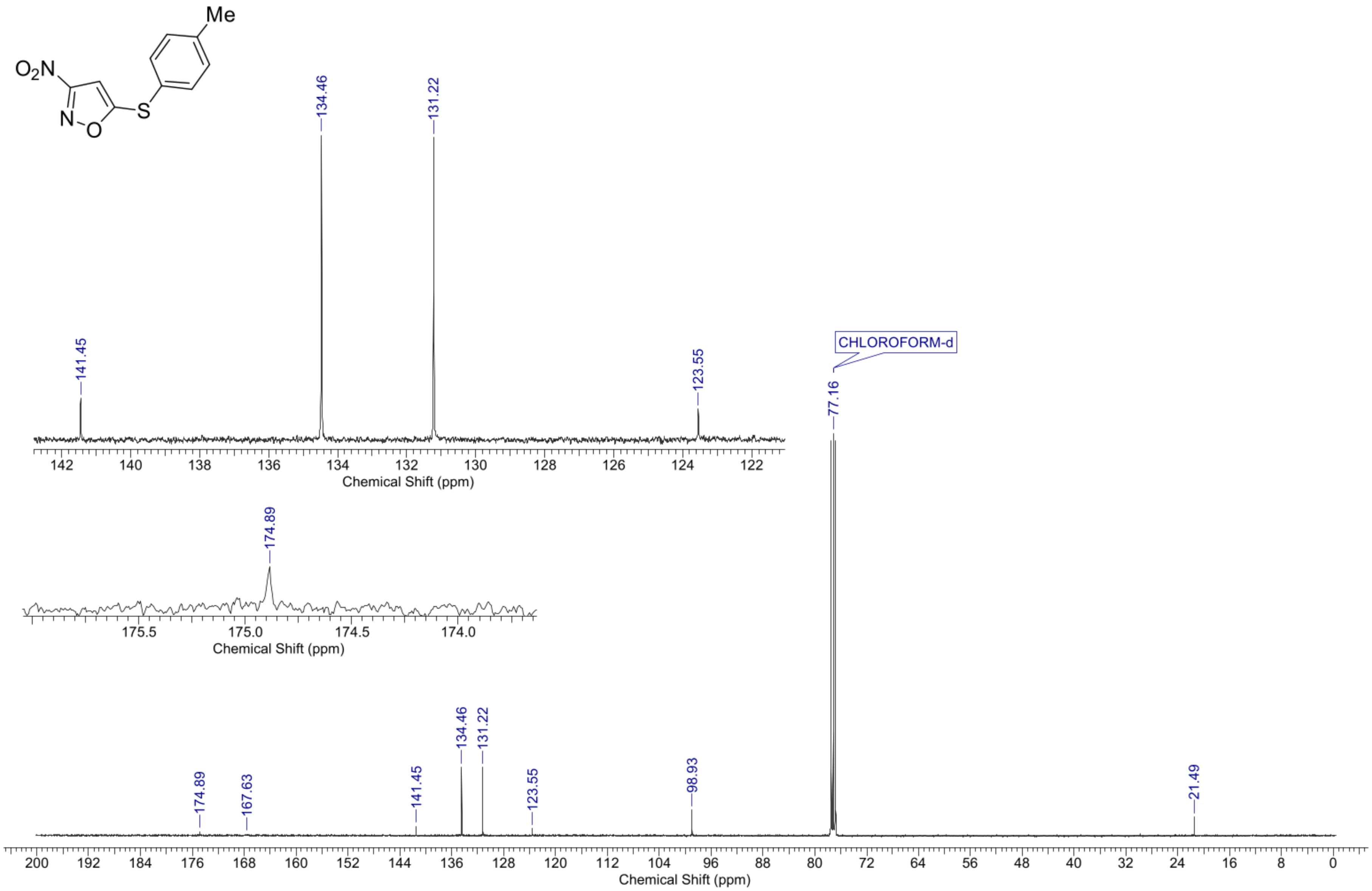

5-((4-Methoxyphenyl)thio)-3-nitroisoxazole **2k** (^{1}H NMR)

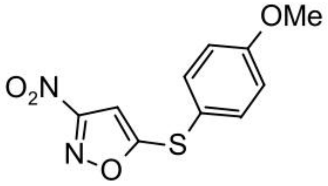

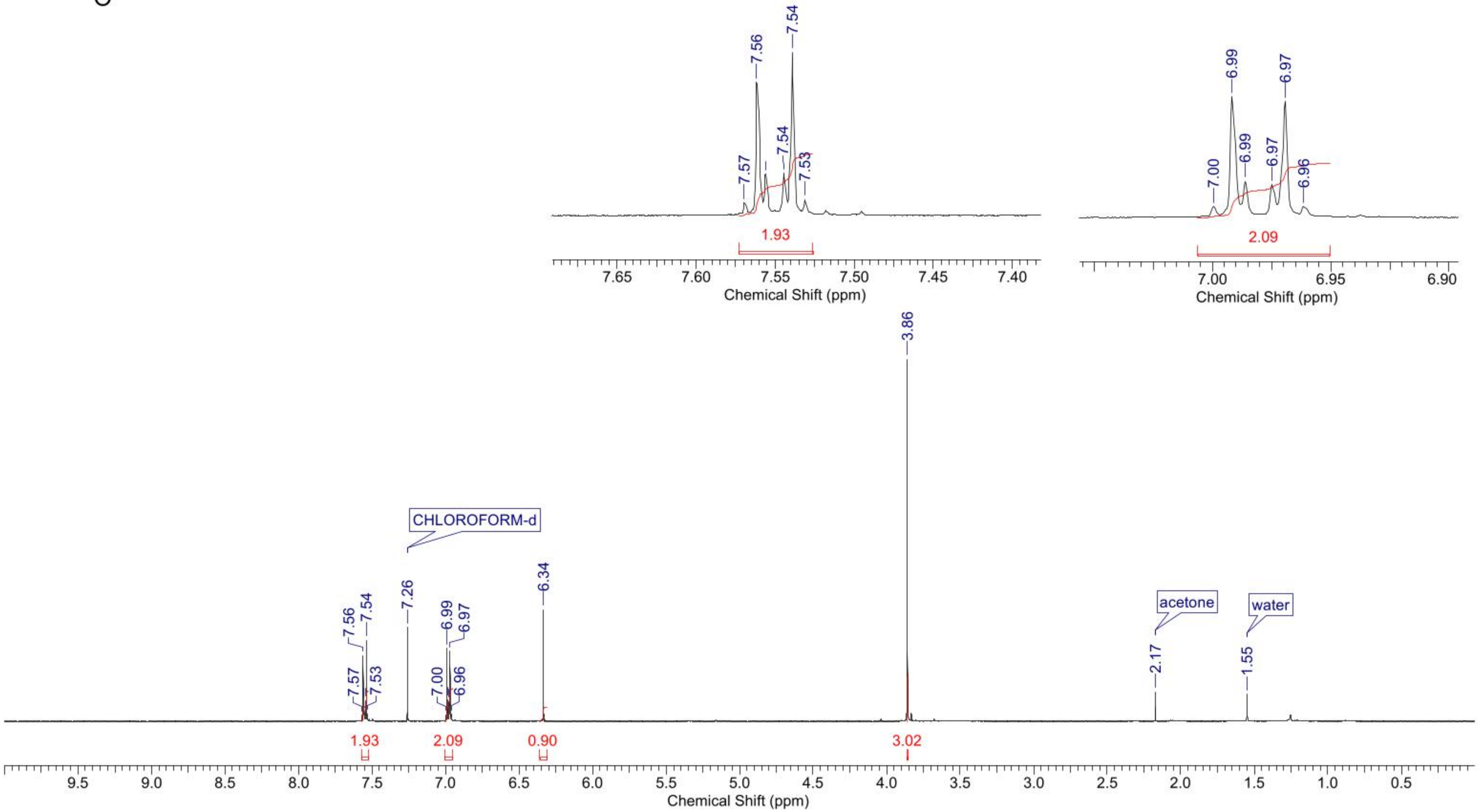

5-((4-Methoxyphenyl)thio)-3-nitroisoxazole **2k** (^{13}C NMR)

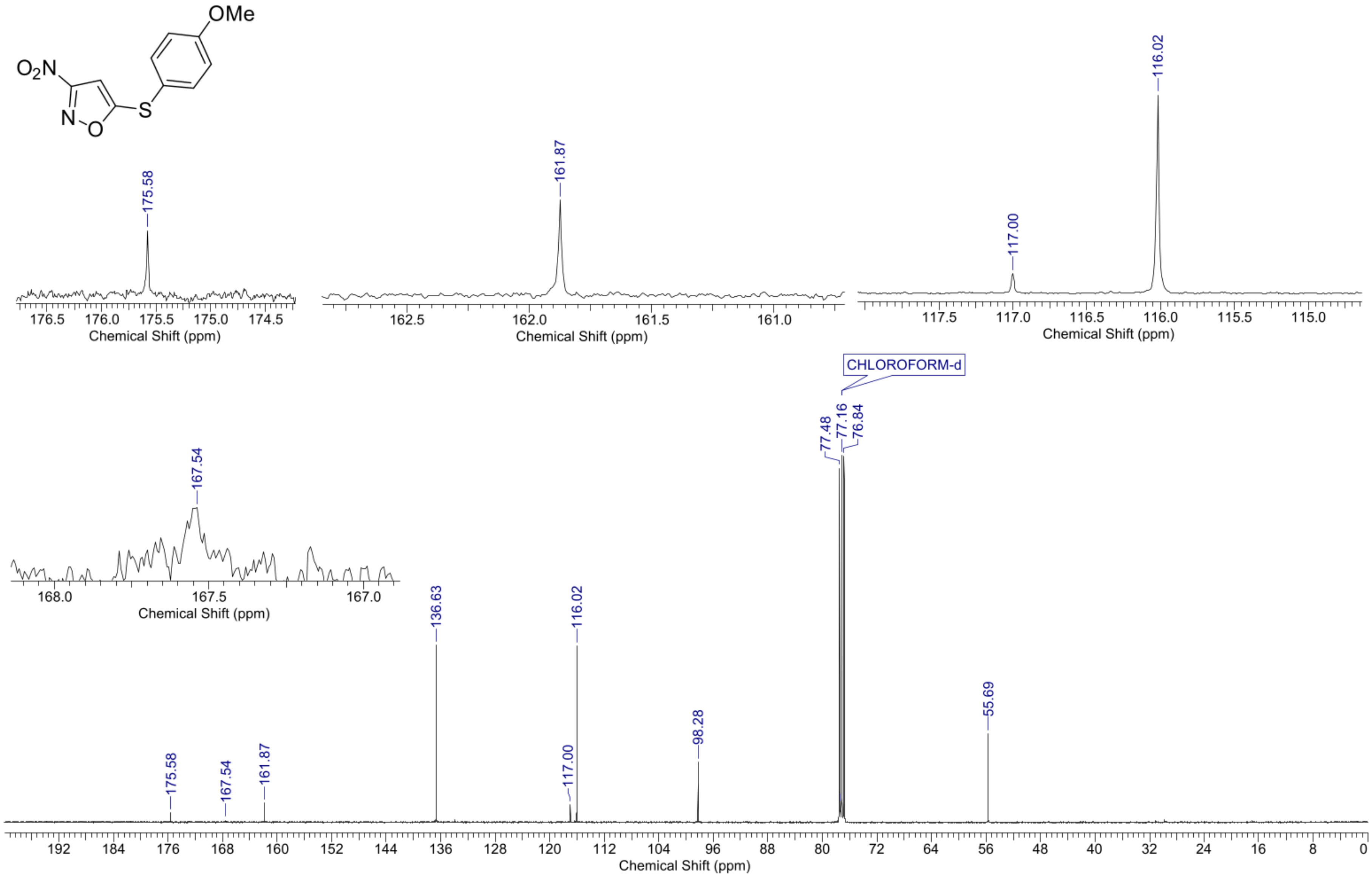

5-((4-Methoxyphenyl)thio)-3-nitroisoxazole **2k** (HMBC)

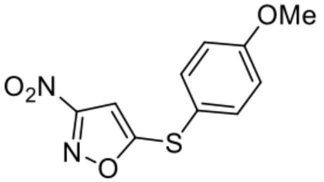

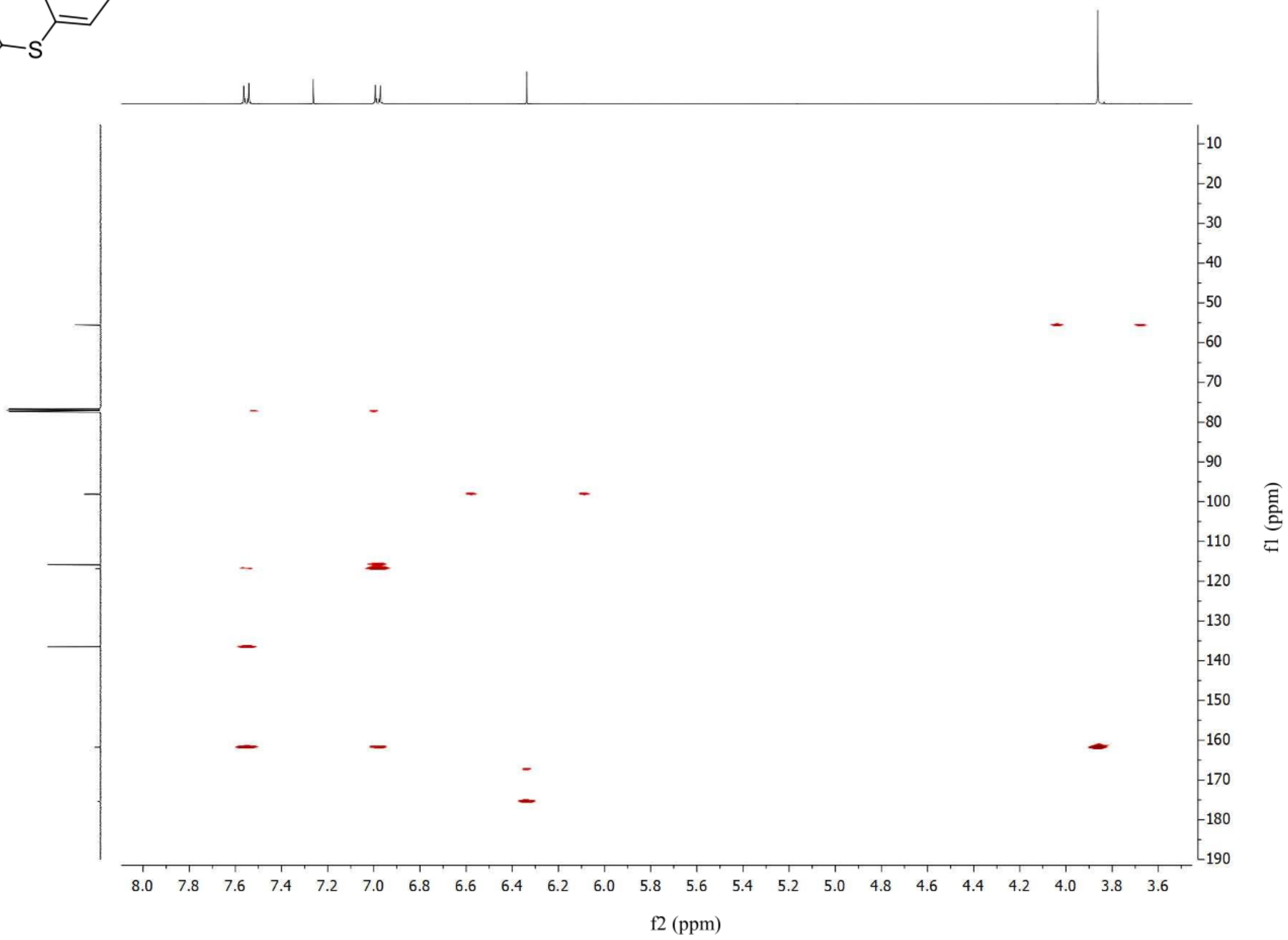

Methyl 5-((3,4-dimethylphenyl)thio)isoxazole-3-carboxylate **2l** (^{1}H NMR)

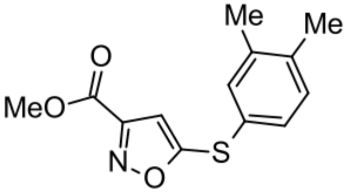

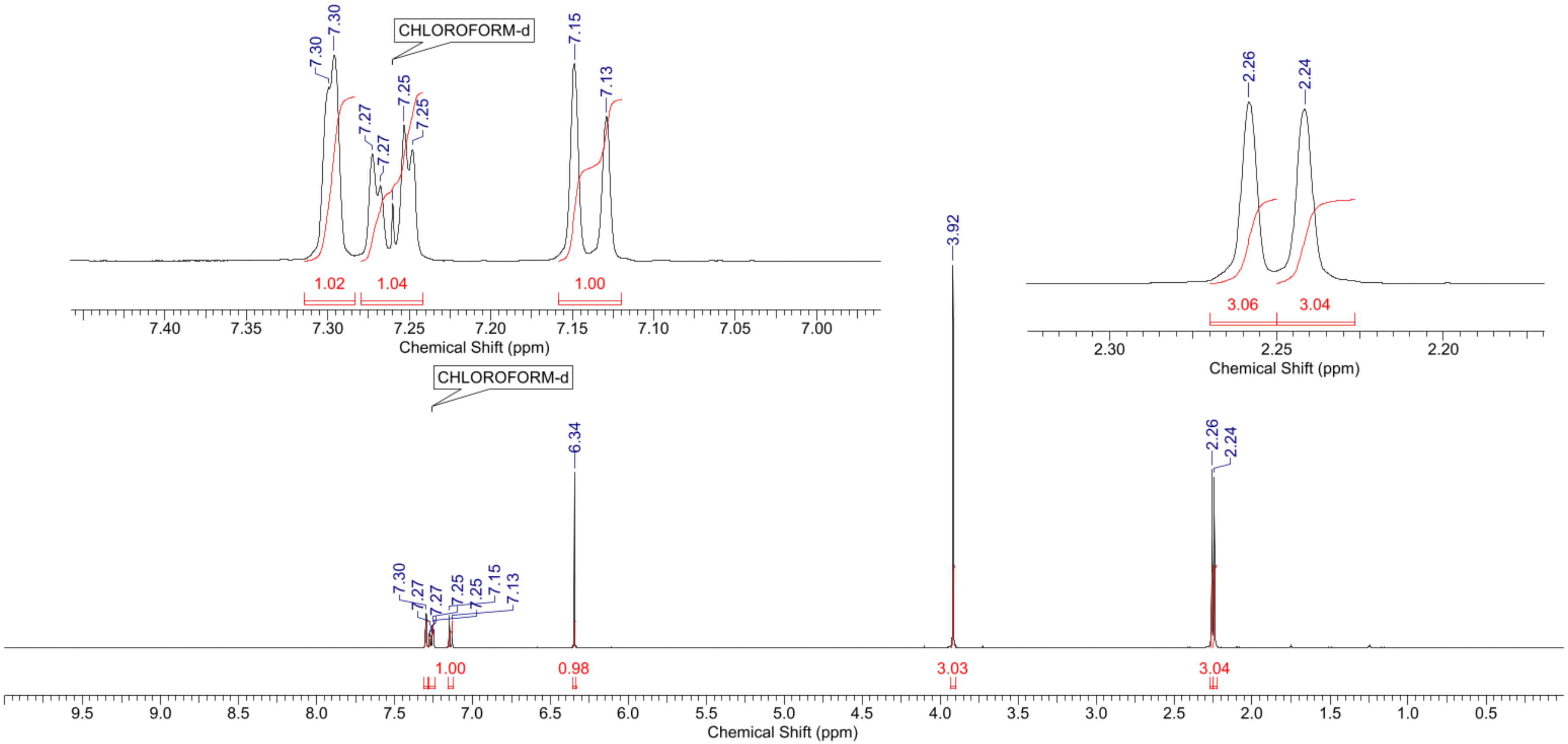

Methyl 5-((3,4-dimethylphenyl)thio)isoxazole-3-carboxylate **2l** (^{13}C NMR)

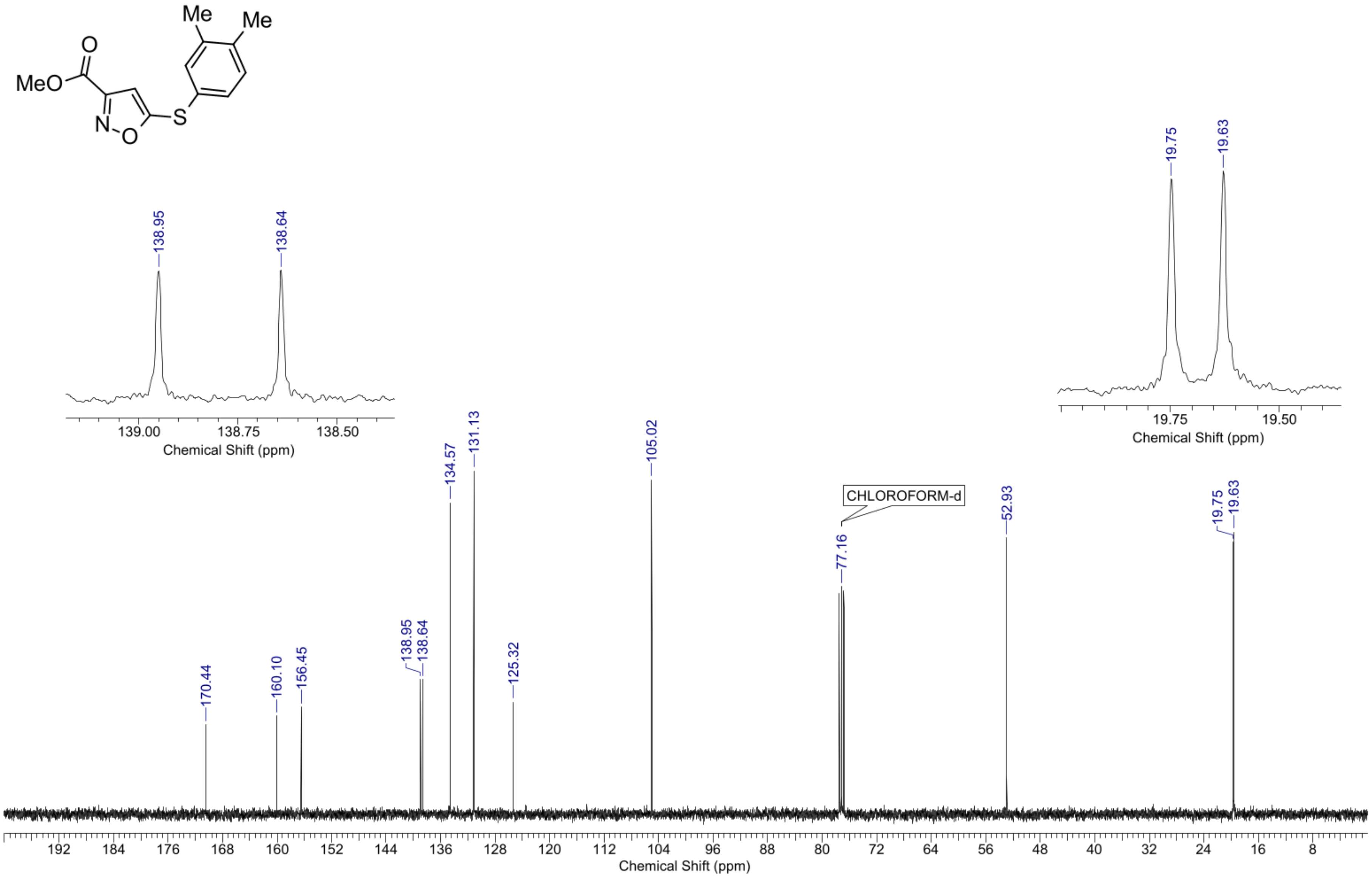

Methyl 5-((4-chlorophenyl)thio)isoxazole-3-carboxylate **2m** (^{1}H NMR)

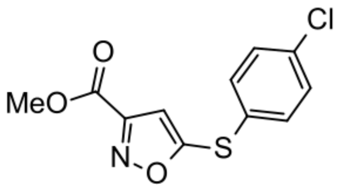

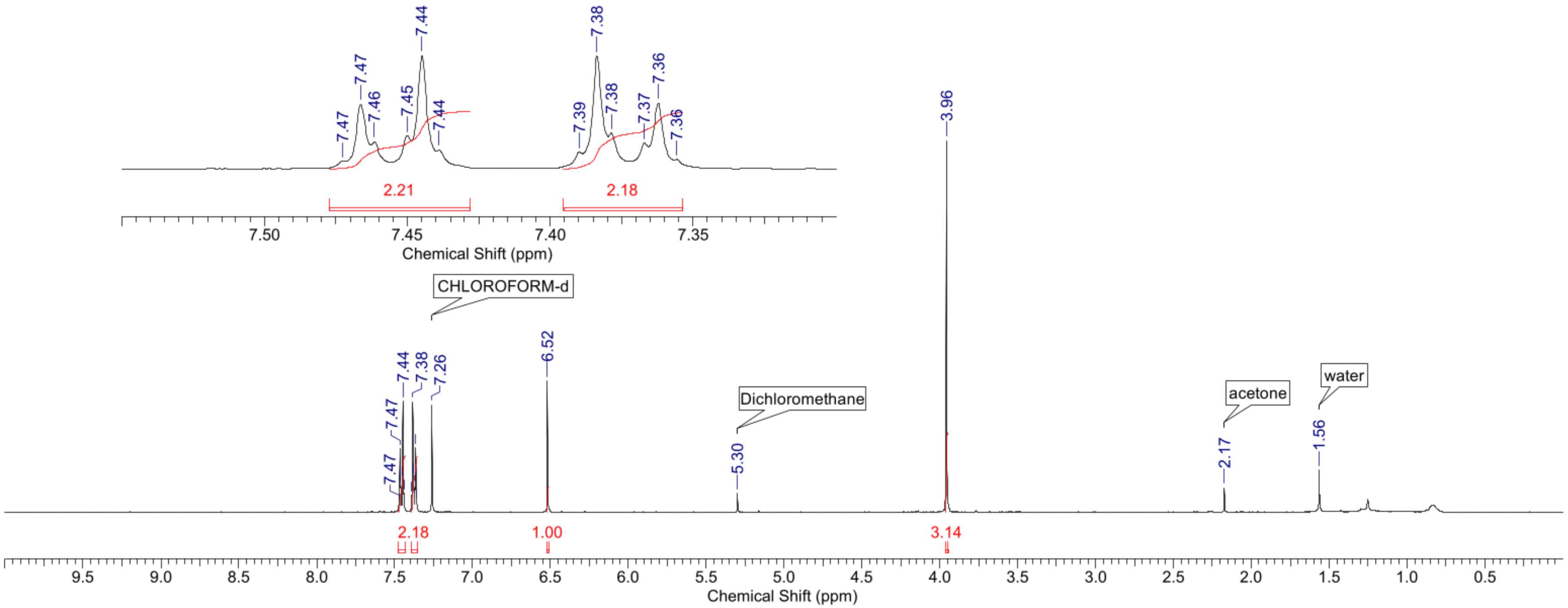

Methyl 5-((4-chlorophenyl)thio)isoxazole-3-carboxylate **2m** (^{13}C NMR)

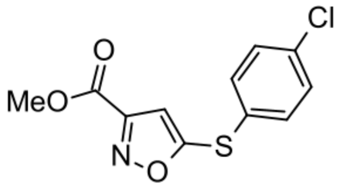

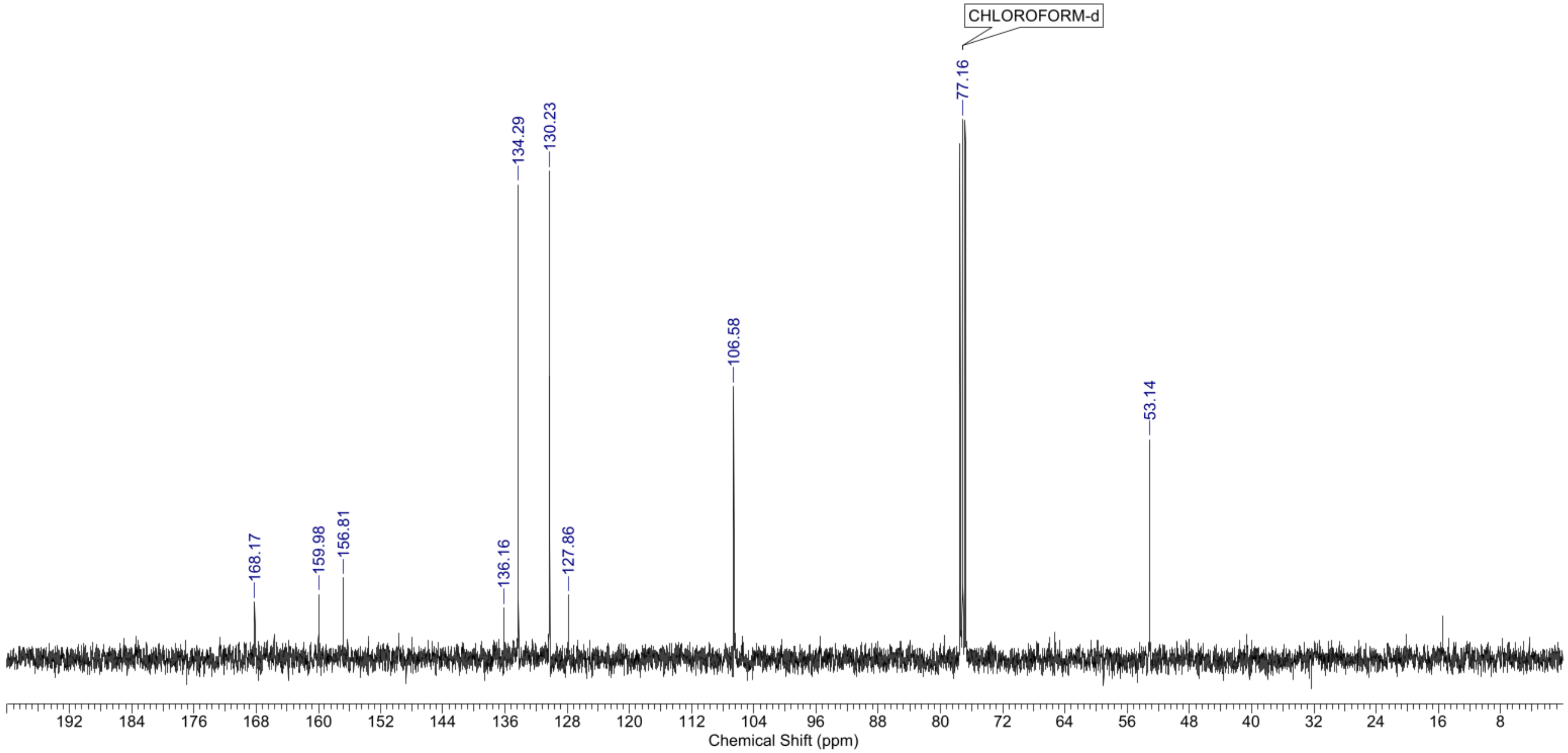

Methyl 5-((4-chlorophenyl)thio)isoxazole-3-carboxylate **2m** (HSQC)

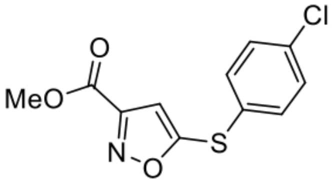

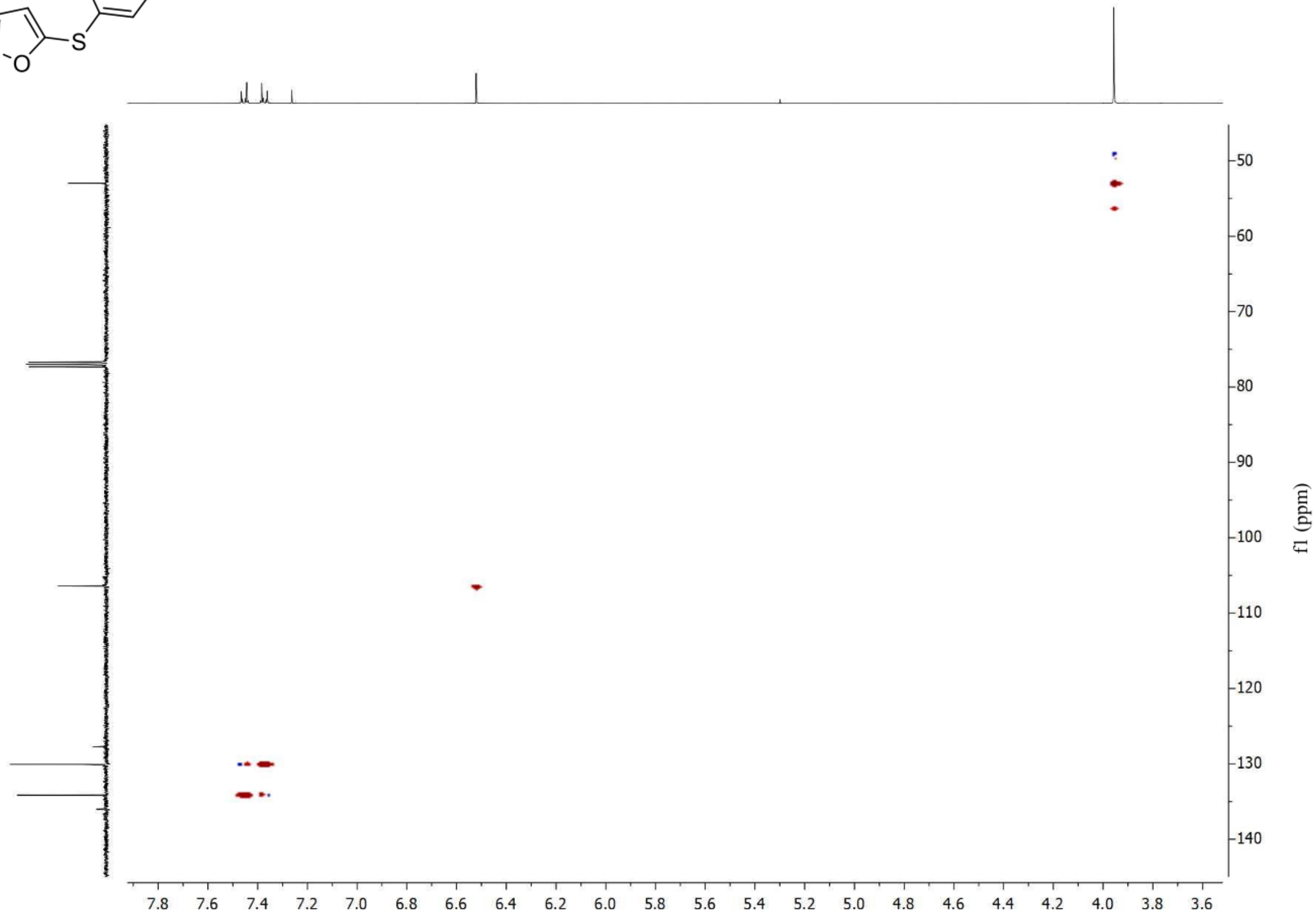

Methyl 5-((4-chlorophenyl)thio)isoxazole-3-carboxylate **2m** (HMBC)

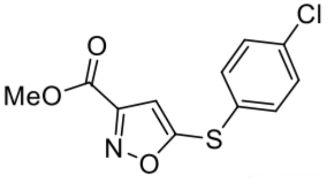

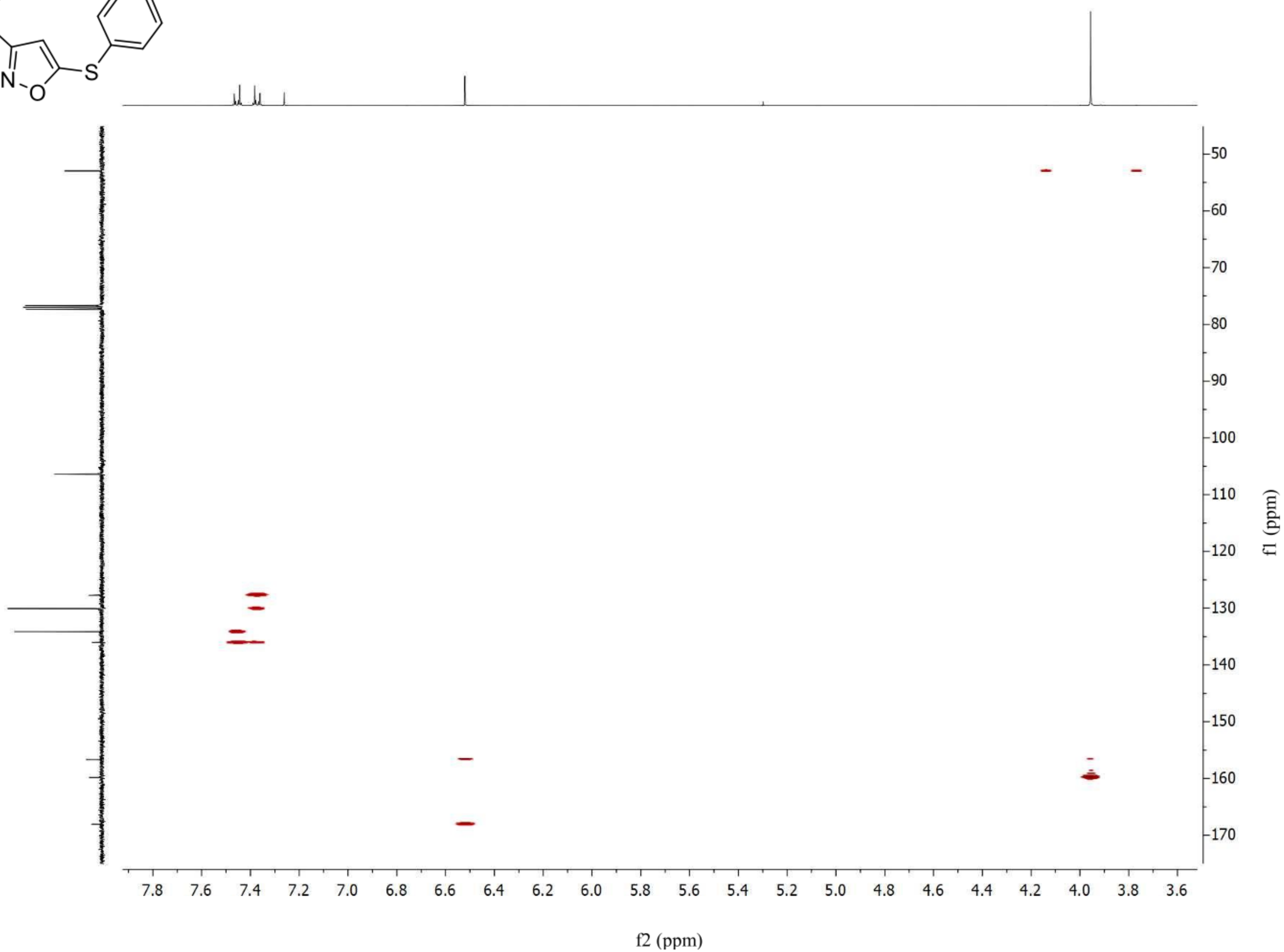

Methyl 5-((4-fluorophenyl)thio)isoxazole-3-carboxylate **2n** (^{1}H NMR)

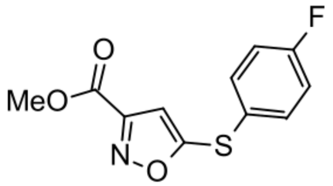

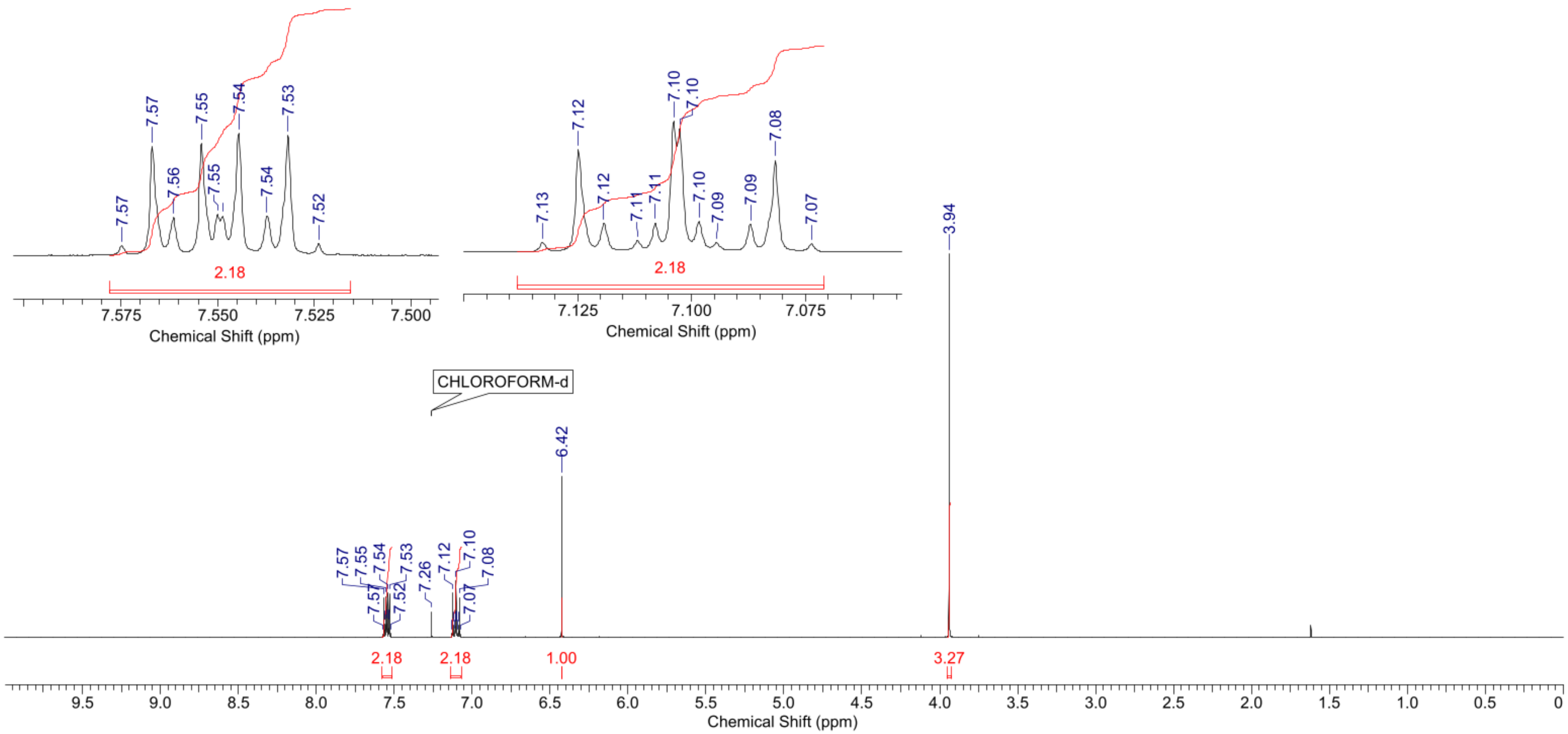

Methyl 5-((4-fluorophenyl)thio)isoxazole-3-carboxylate **2n** (^{13}C NMR)

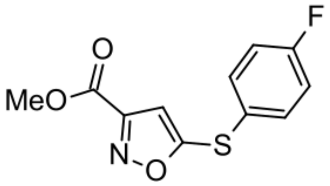

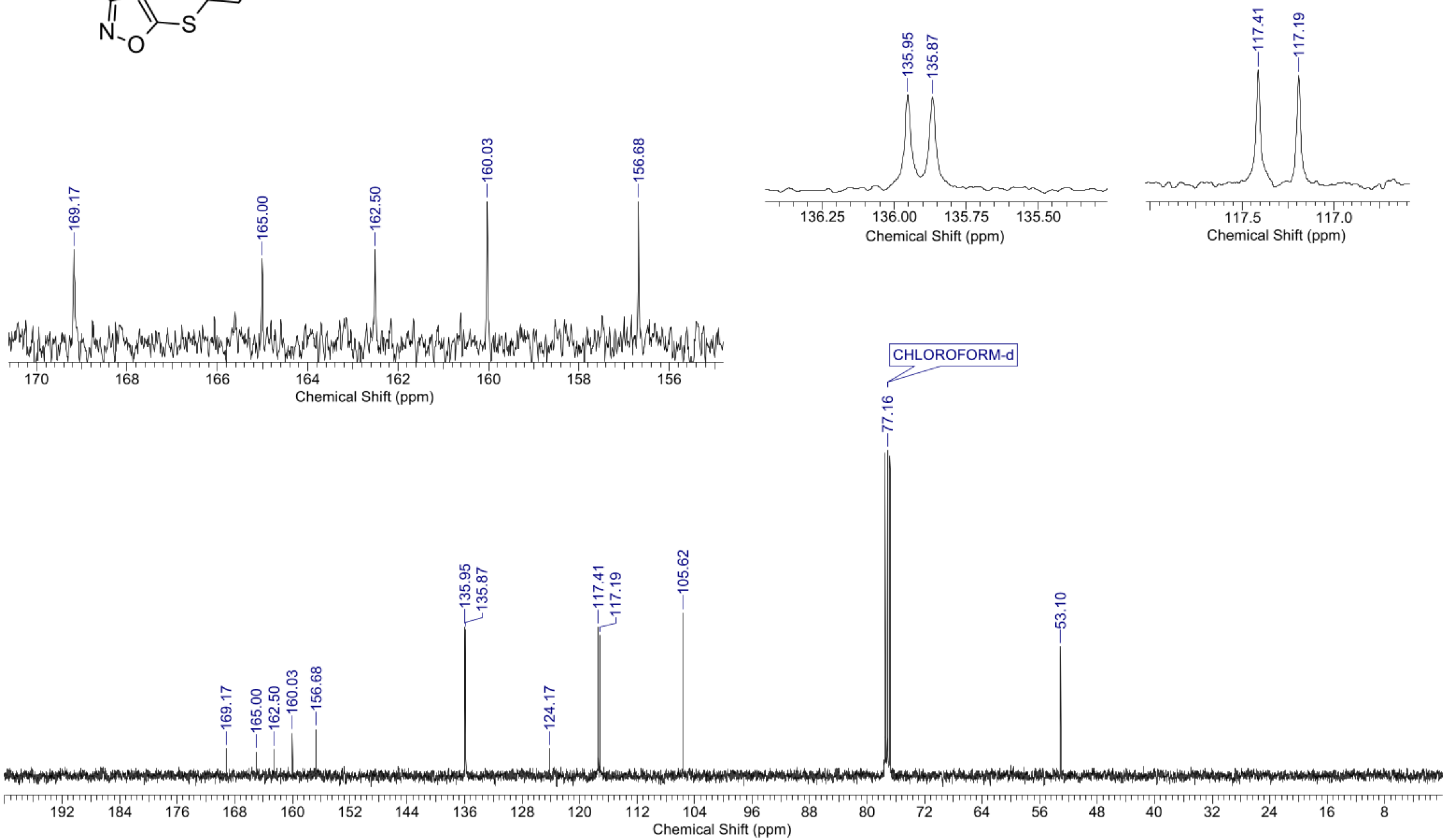

Methyl 5-((4-fluorophenyl)thio)isoxazole-3-carboxylate **2n** (^{19}F NMR)

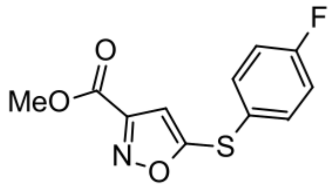

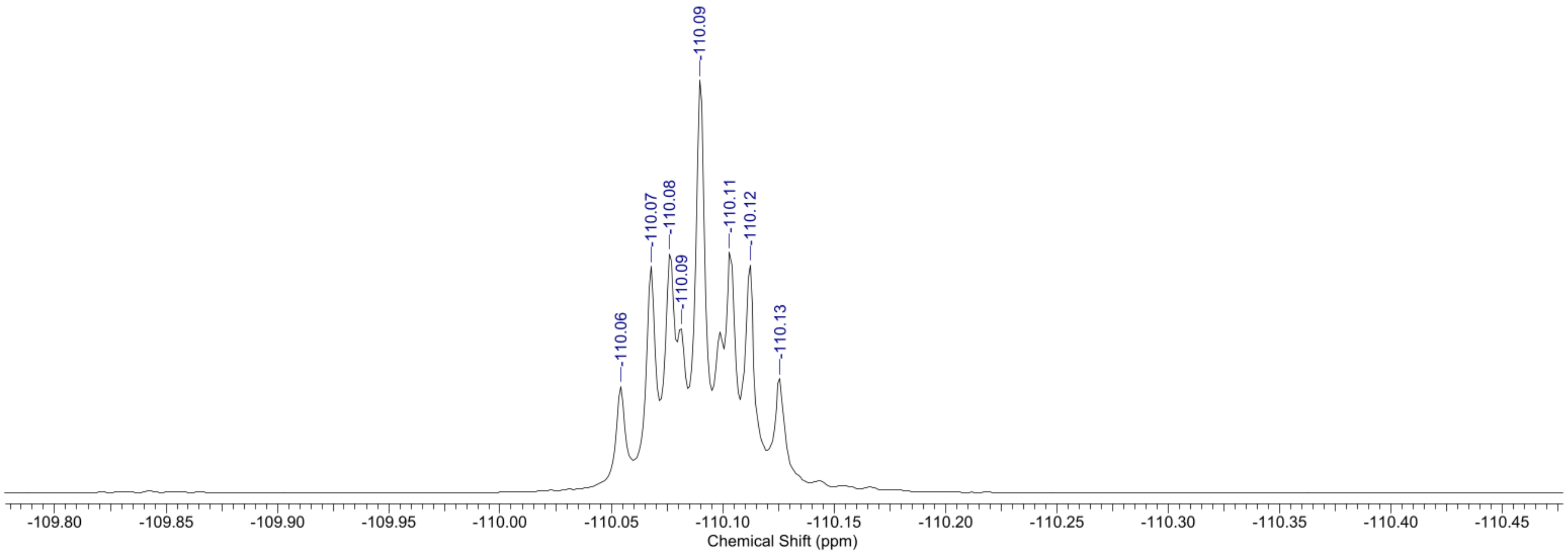

Methyl 5-(p-tolylthio)isoxazole-3-carboxylate **2o** (^{1}H NMR)

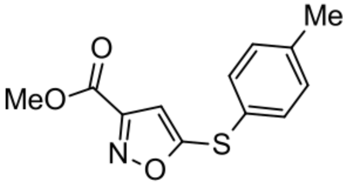

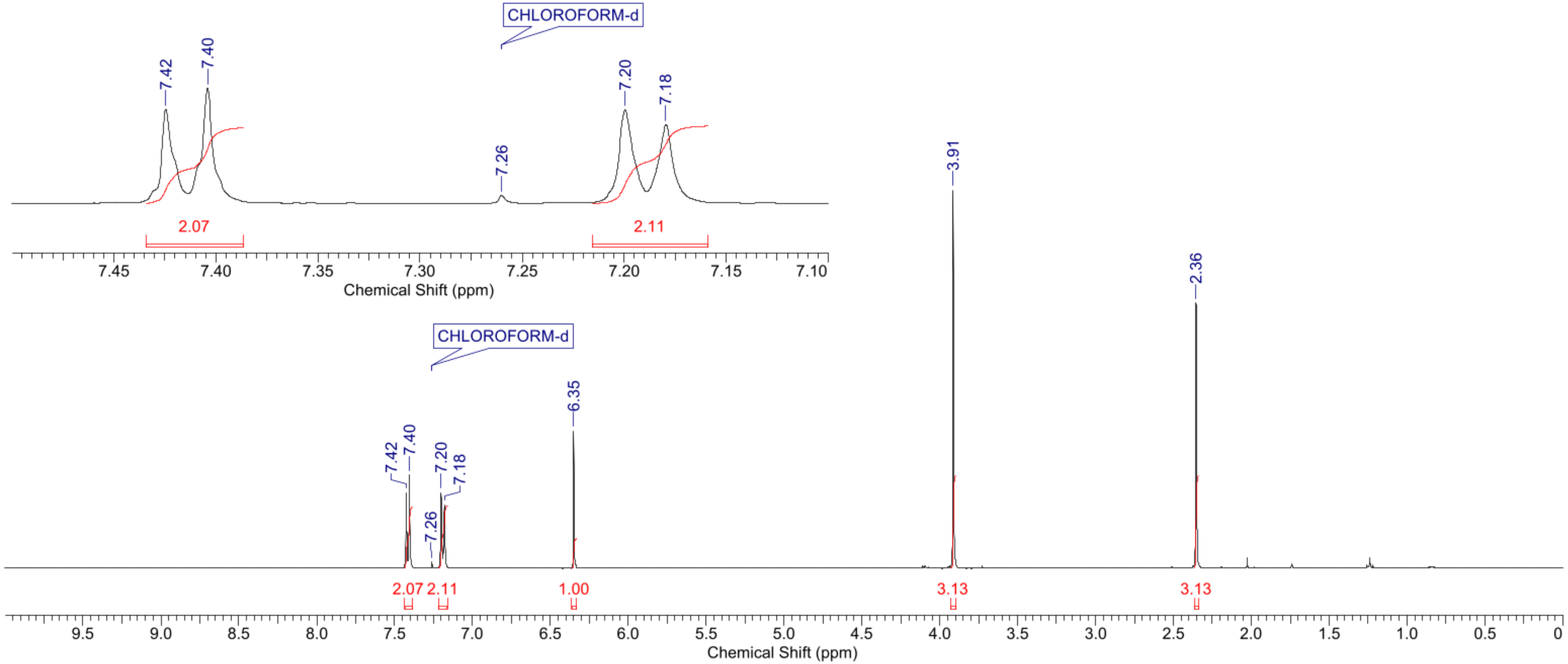

Methyl 5-(p-tolylthio)isoxazole-3-carboxylate **2o** (^{13}C NMR)

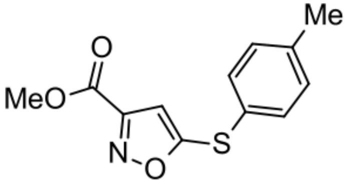

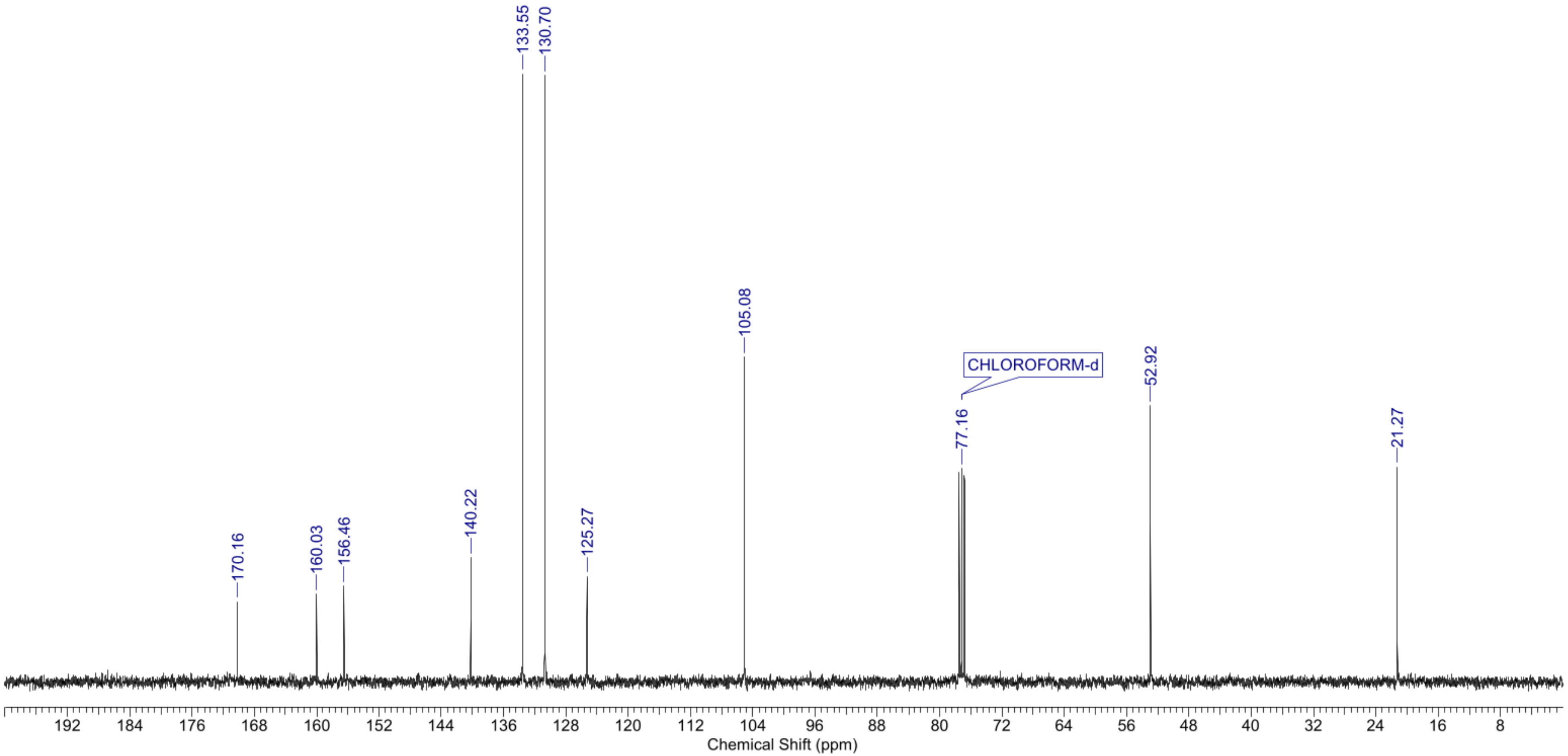

Methyl 5-((4-methoxyphenyl)thio)isoxazole-3-carboxylate **2p** (^{1}H NMR)

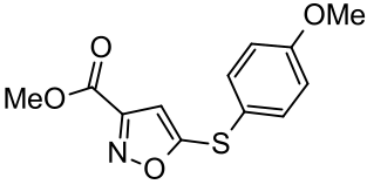

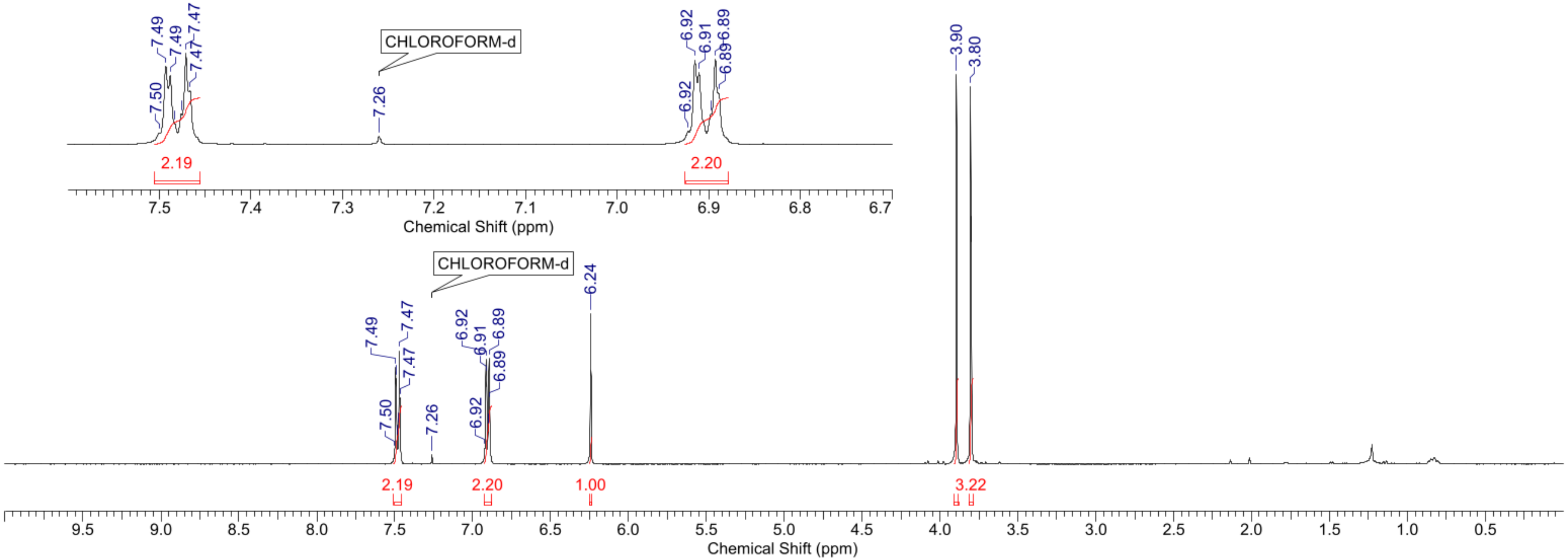

Methyl 5-((4-methoxyphenyl)thio)isoxazole-3-carboxylate **2p** (^{13}C NMR)

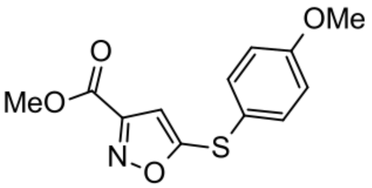

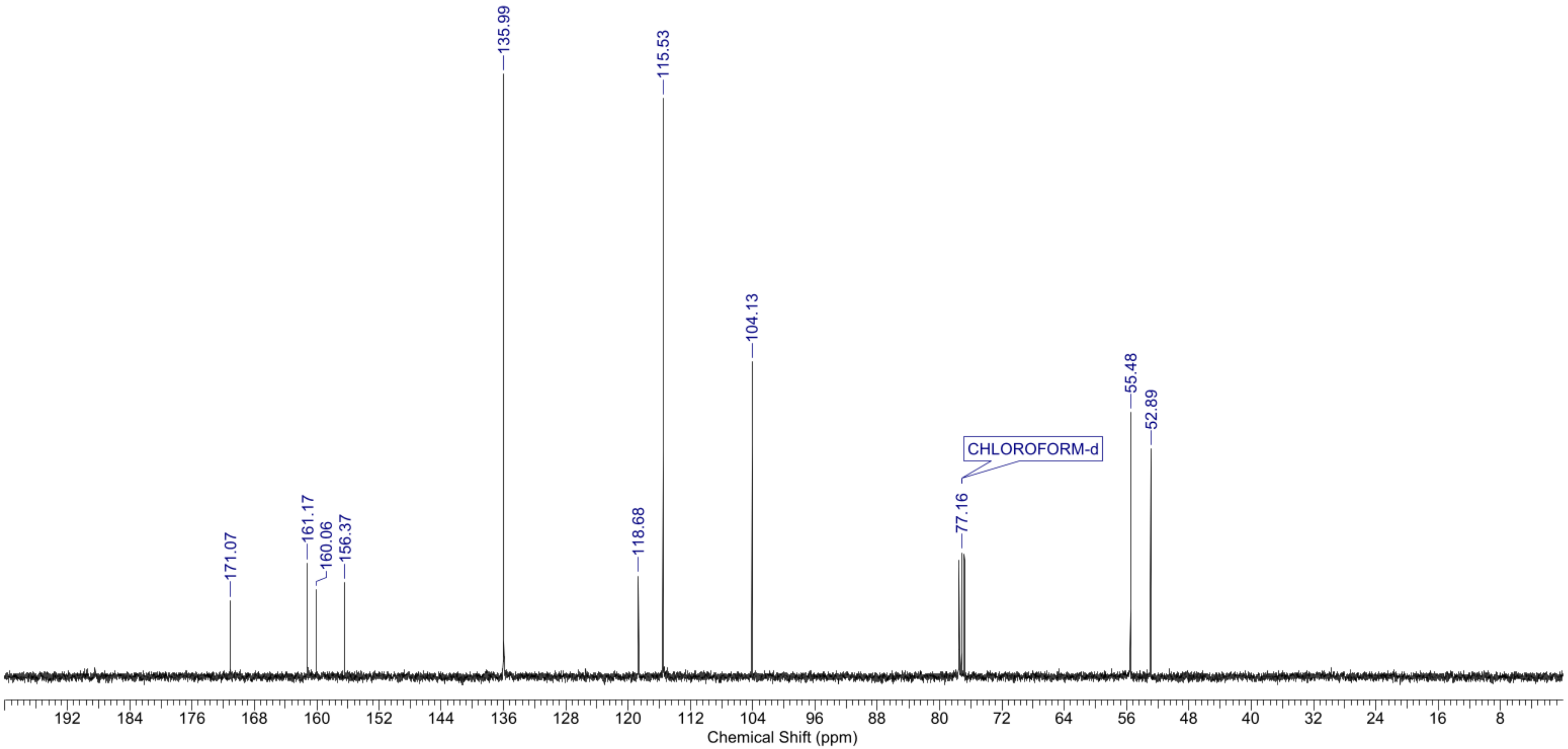

Methyl 5-(phenylsulfonyl)isoxazole-3-carboxylate **3a** (^{1}H NMR)

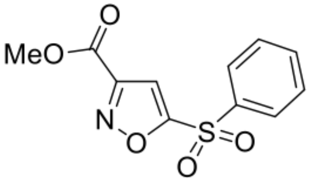

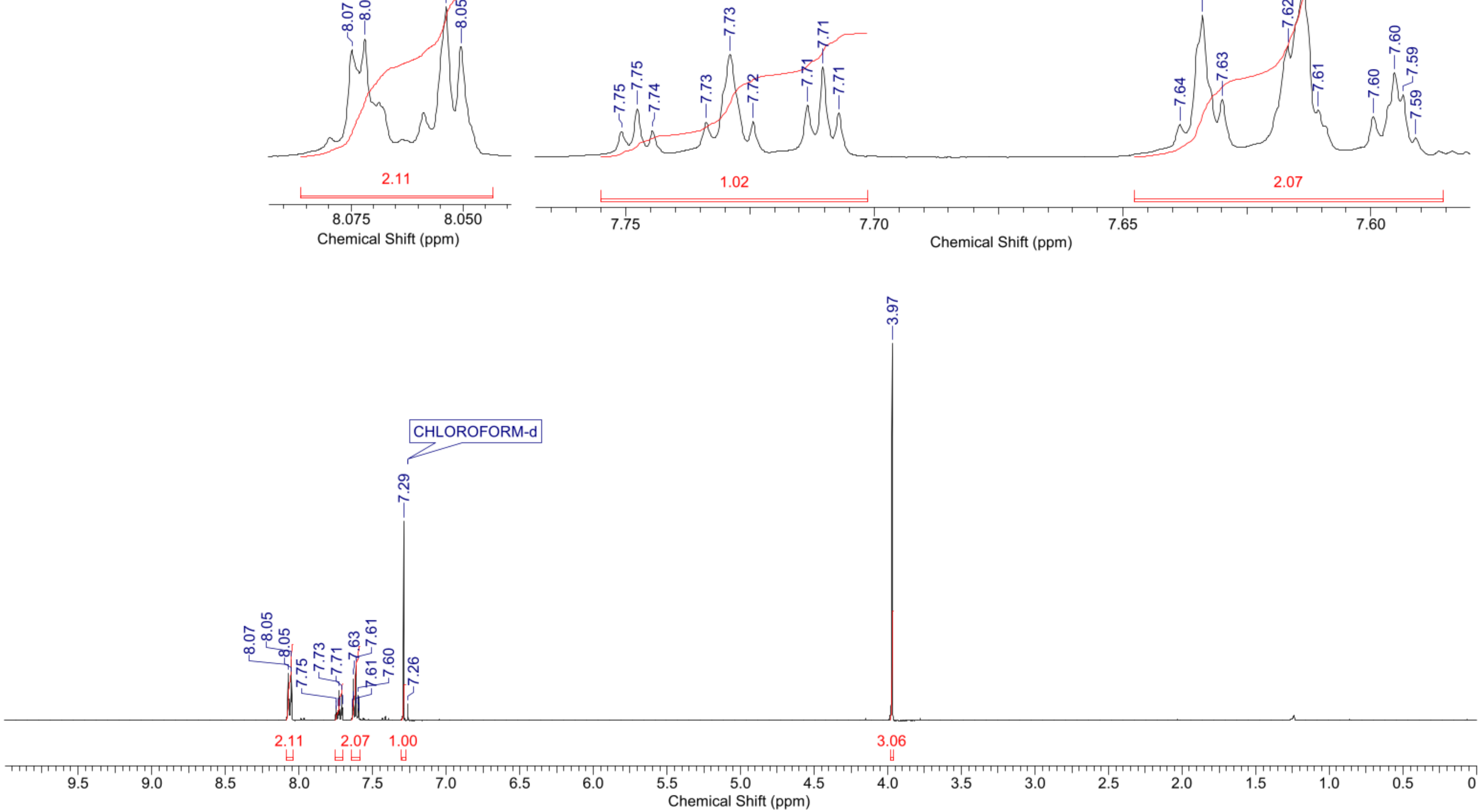

Methyl 5-(phenylsulfonyl)isoxazole-3-carboxylate **3a** (^{13}C NMR)

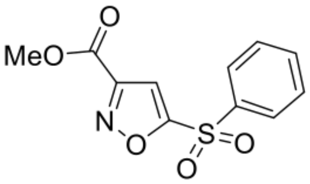

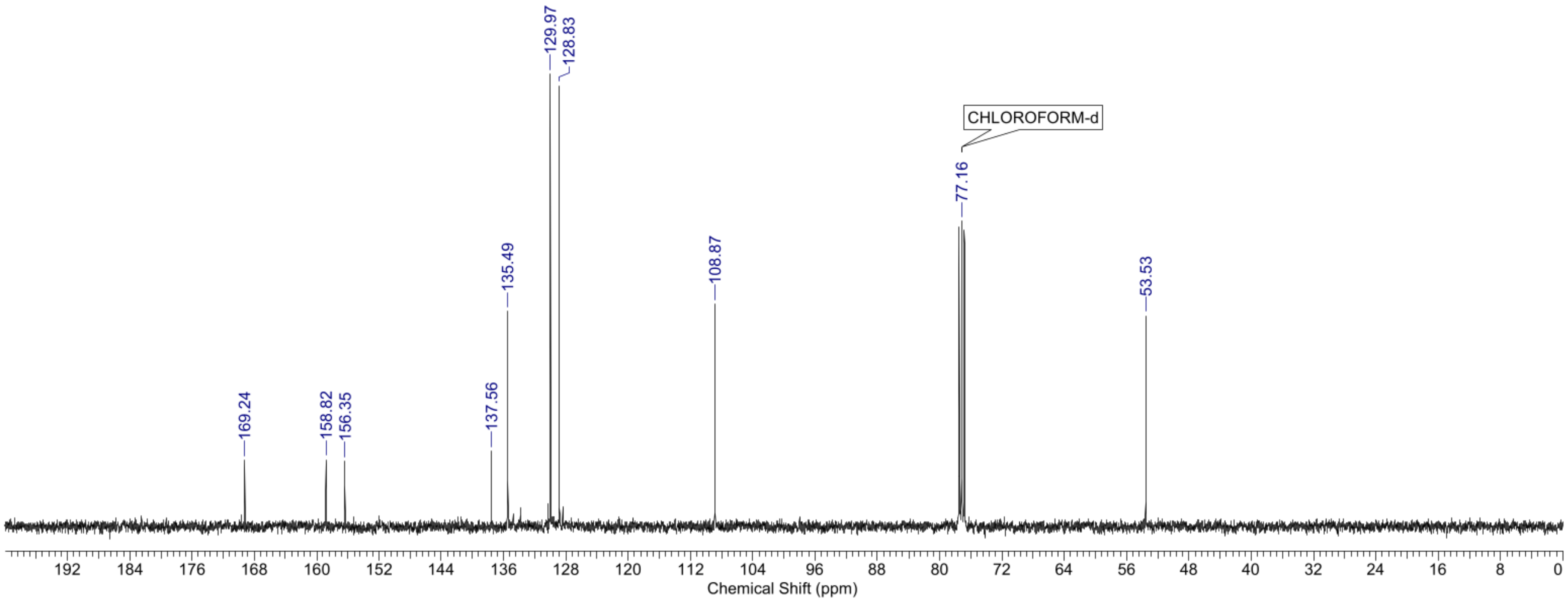

Tert-butyl 5-(phenylsulfonyl)isoxazole-3-carboxylate **3b** (^{1}H NMR)

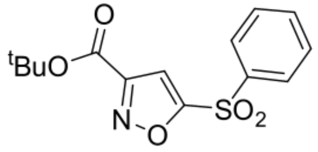

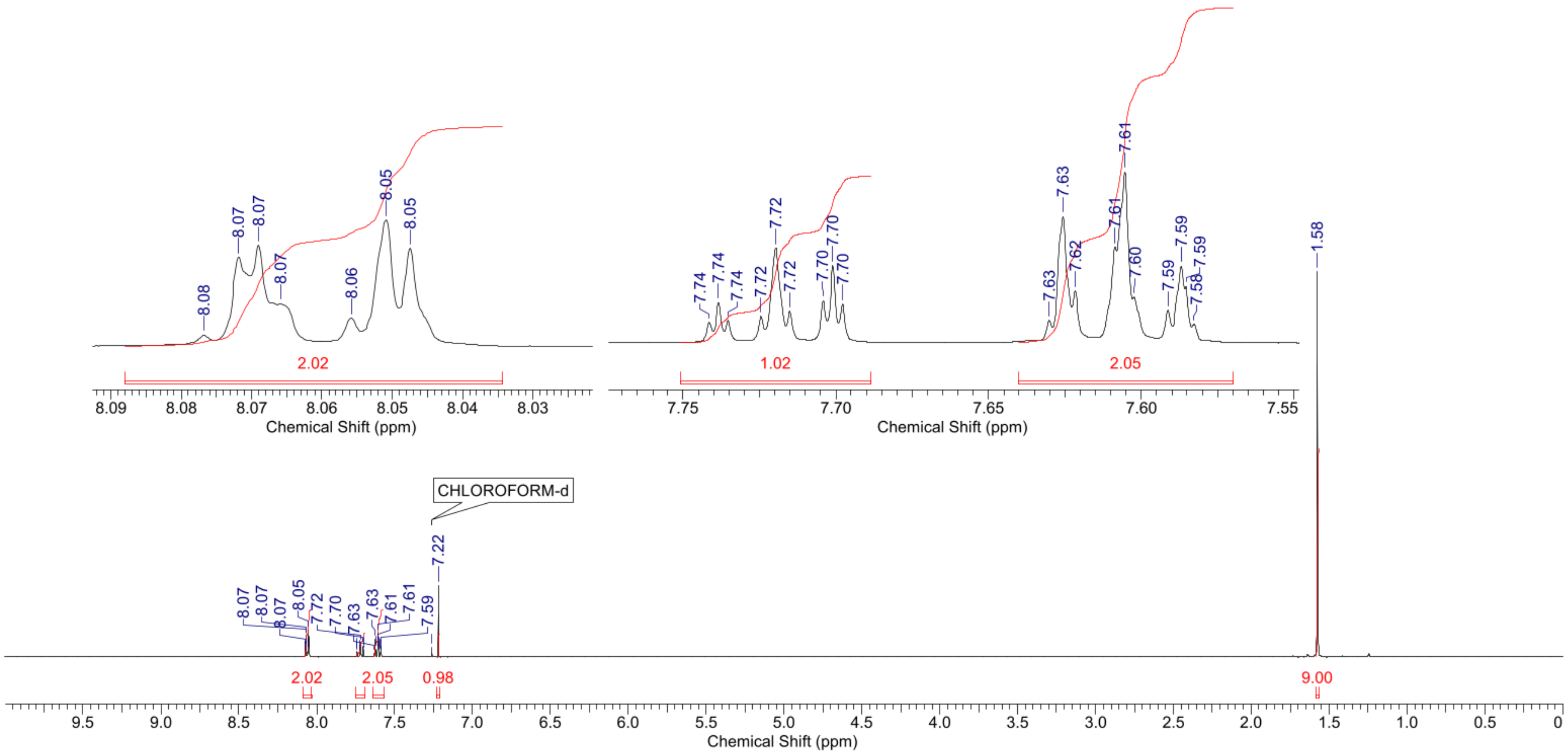

Tert-butyl 5-(phenylsulfonyl)isoxazole-3-carboxylate **3b** (^{13}C NMR)

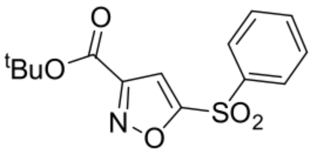

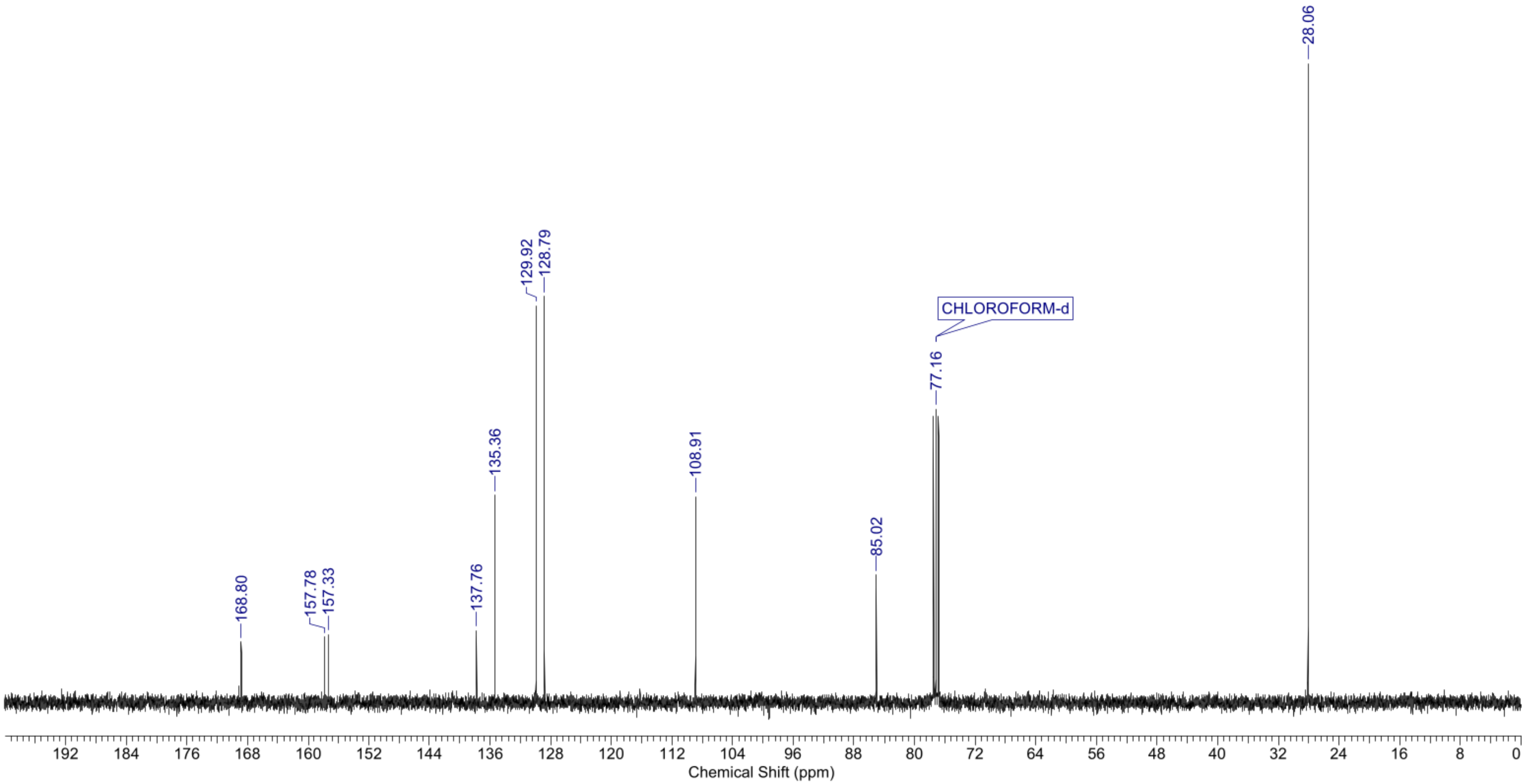

(Adamantan-1-yl)methyl 5-(phenylsulfonyl)isoxazole-3-carboxylate **3c** (^{1}H NMR)

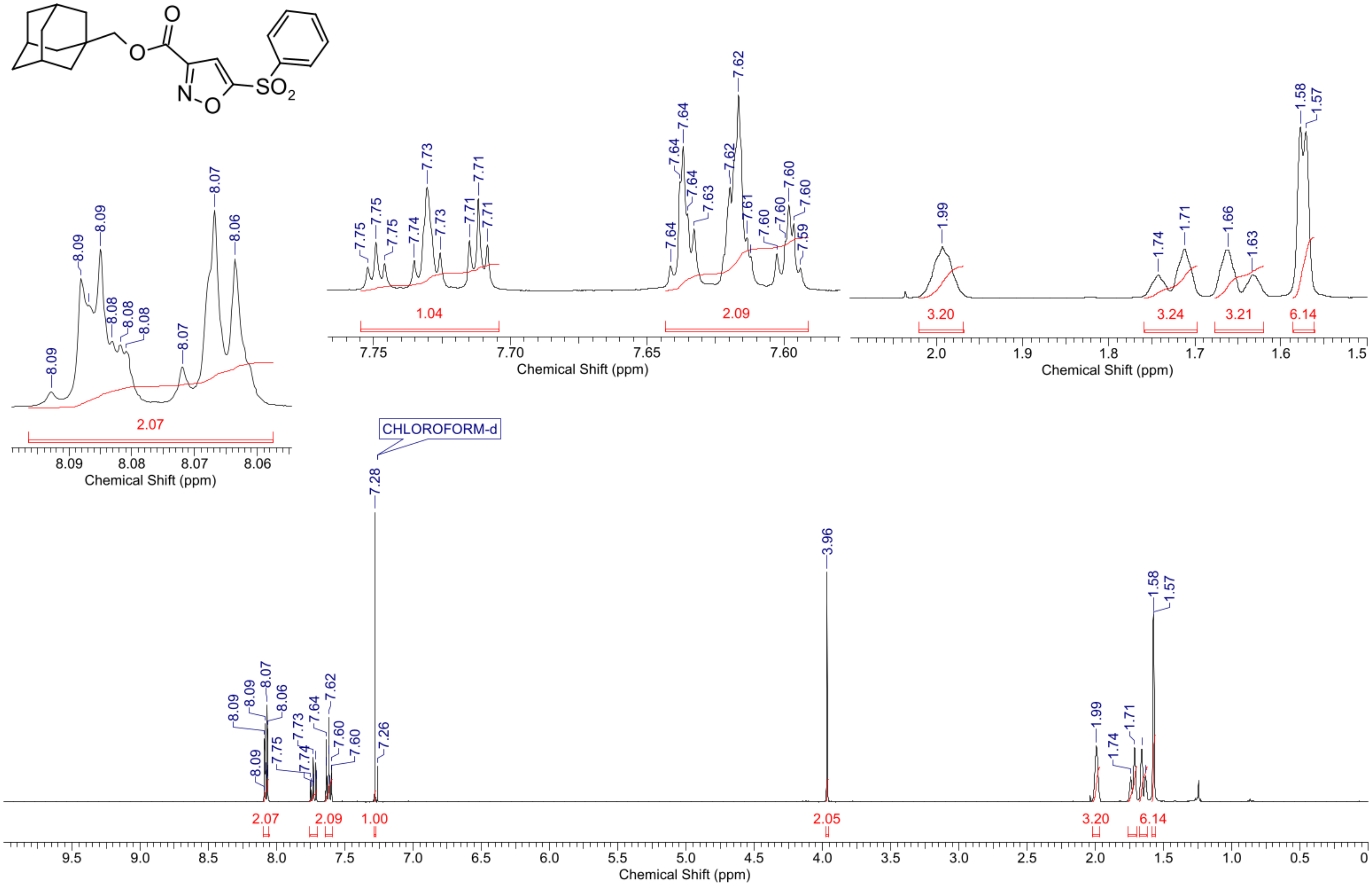

(Adamantan-1-yl)methyl 5-(phenylsulfonyl)isoxazole-3-carboxylate **3c** (^{13}C NMR)

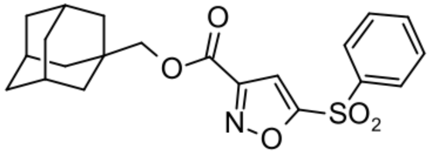

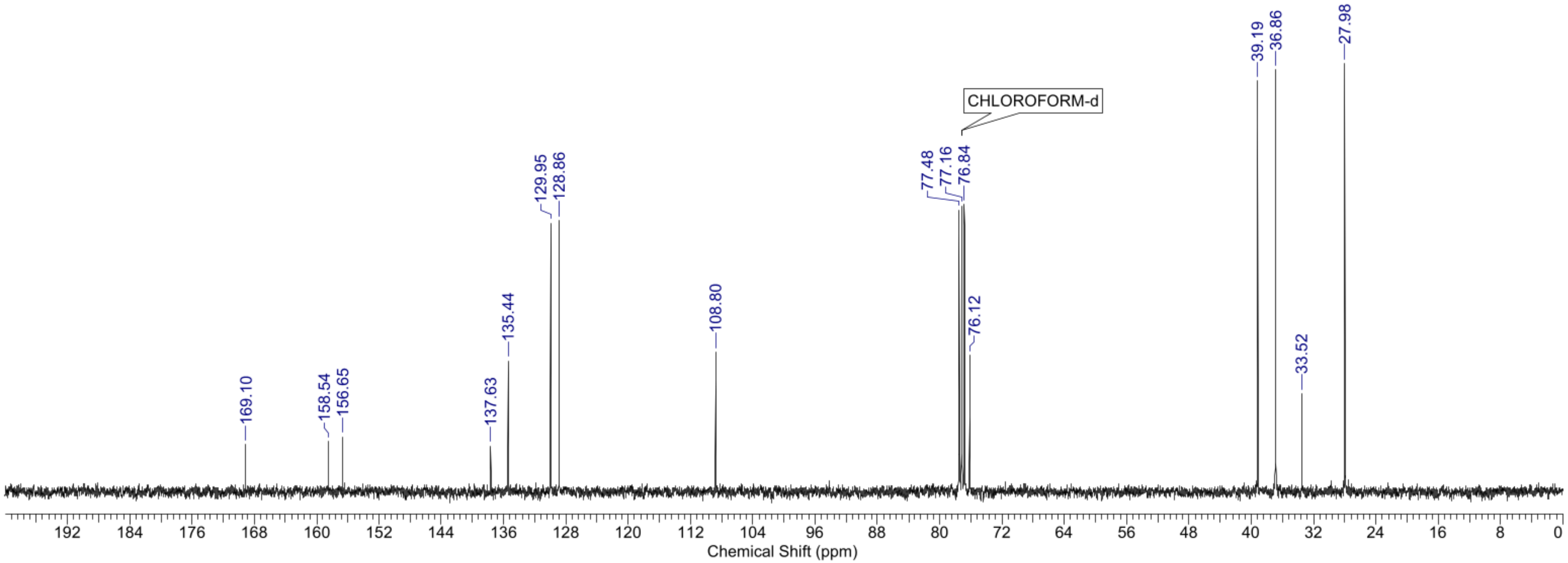

(Adamantan-1-yl)methyl 5-(phenylsulfonyl)isoxazole-3-carboxylate **3c** (APT)

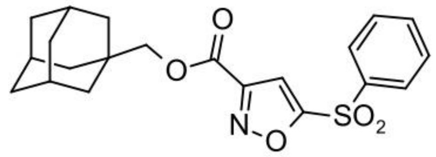

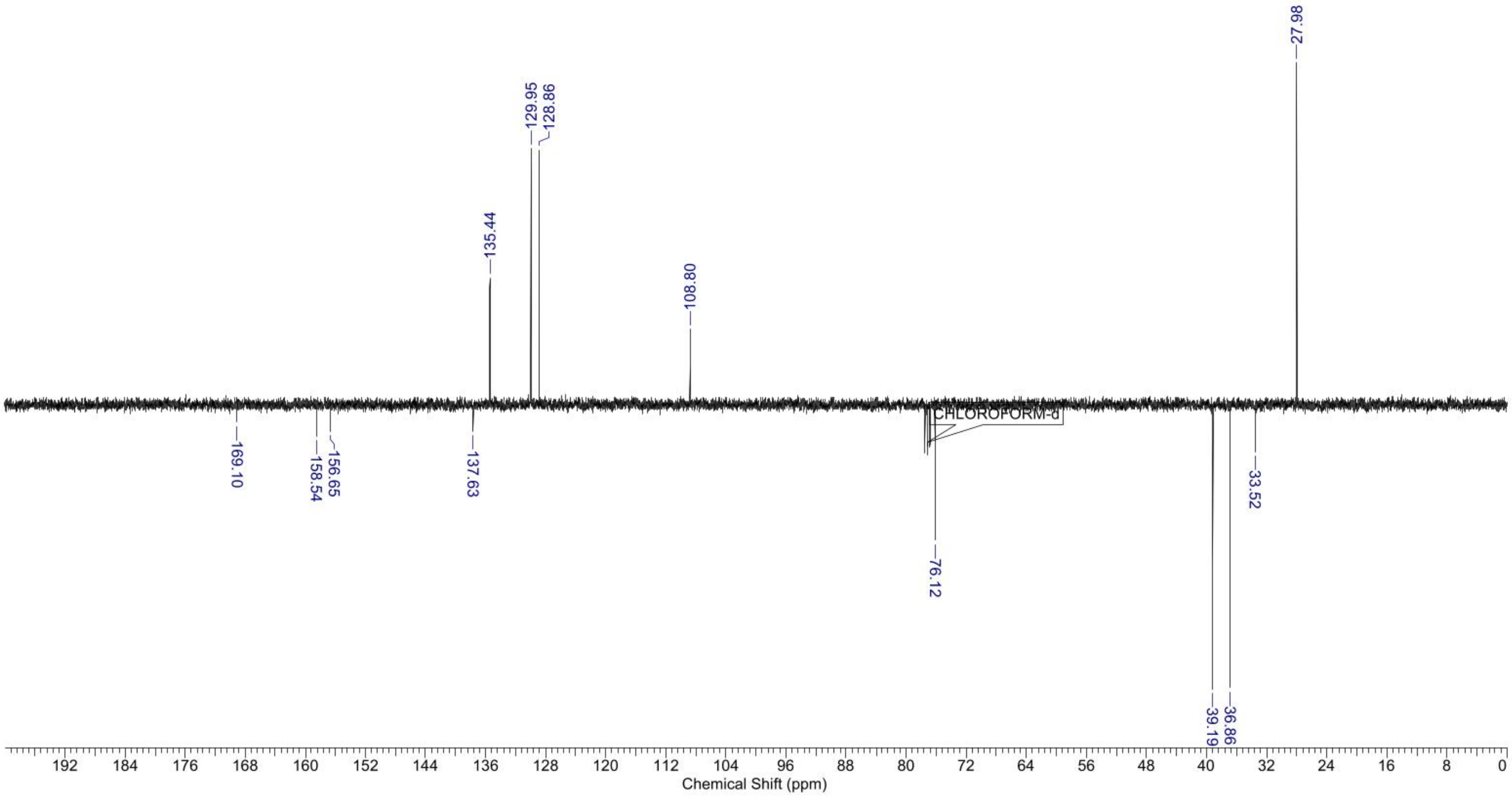

1-(5-(Phenylsulfonyl)isoxazol-3-yl)ethan-1-one **3d** (^{1}H NMR)

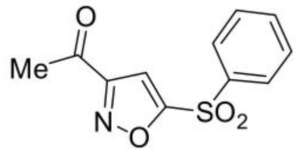

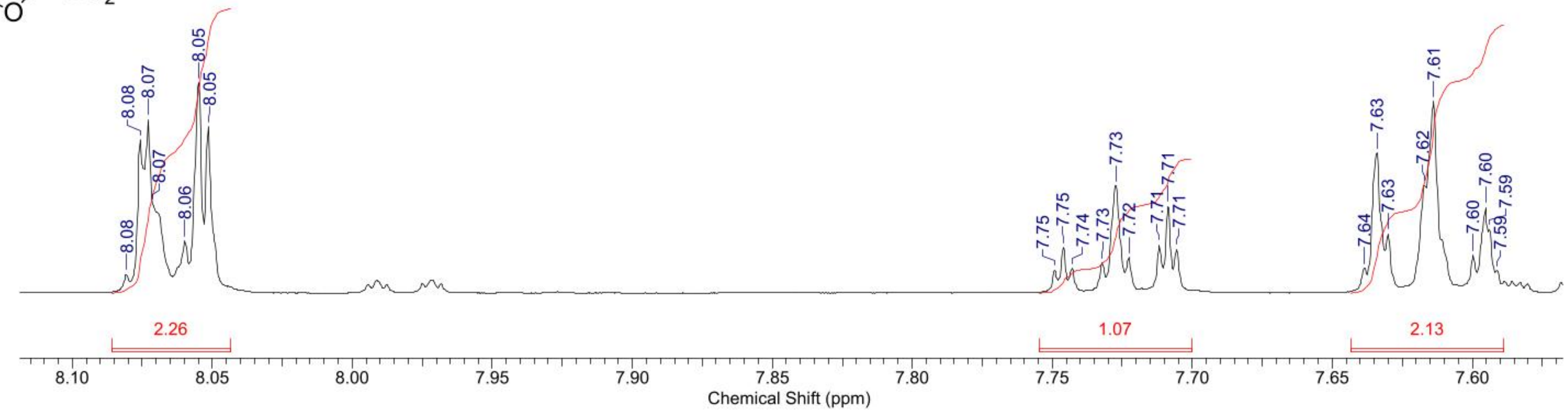

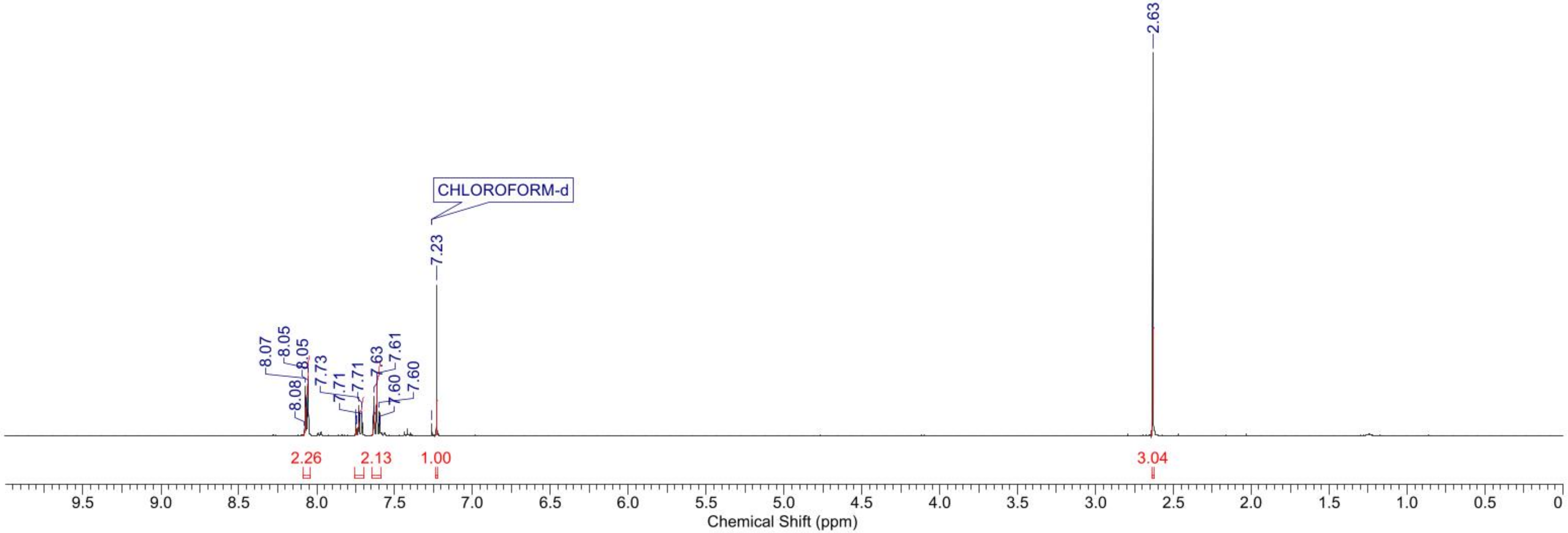

1-(5-(Phenylsulfonyl)isoxazol-3-yl)ethan-1-one **3d** (^{13}C NMR)

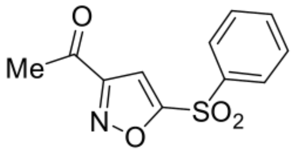

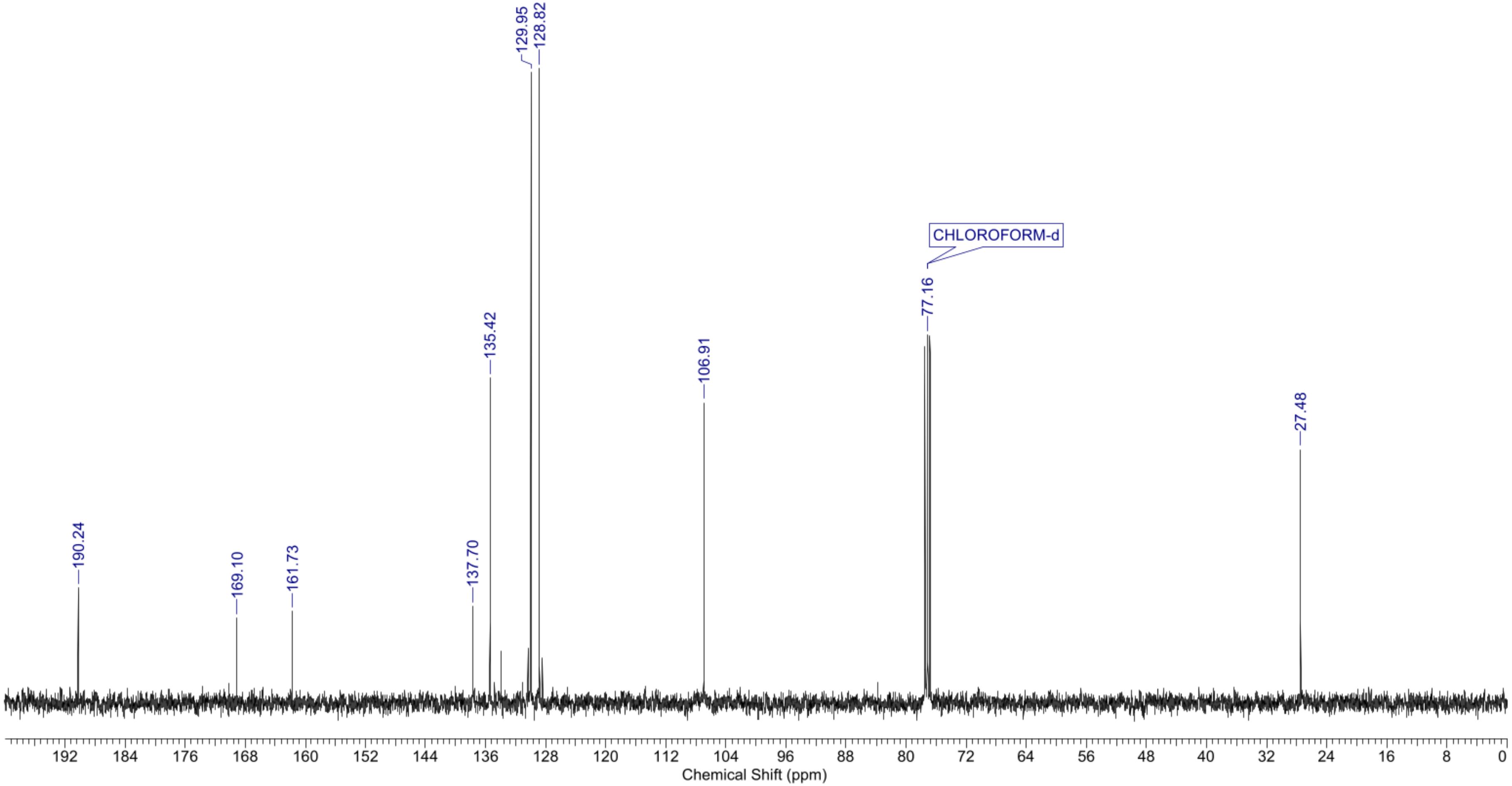

Phenyl(5-(phenylsulfonyl)isoxazol-3-yl)methanone **3e** (^{1}H NMR)

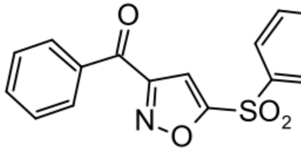

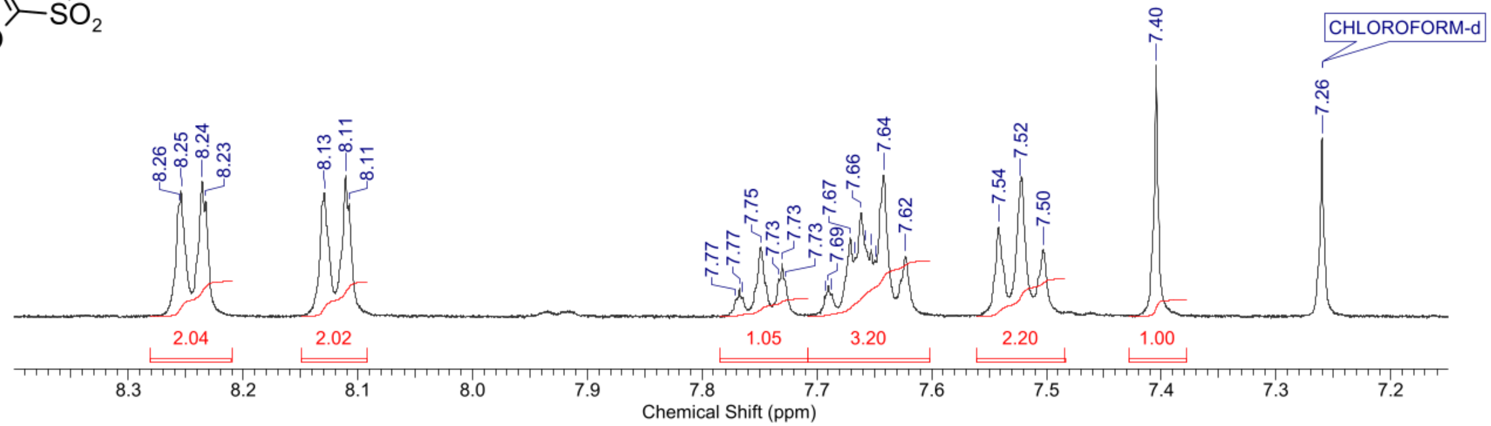

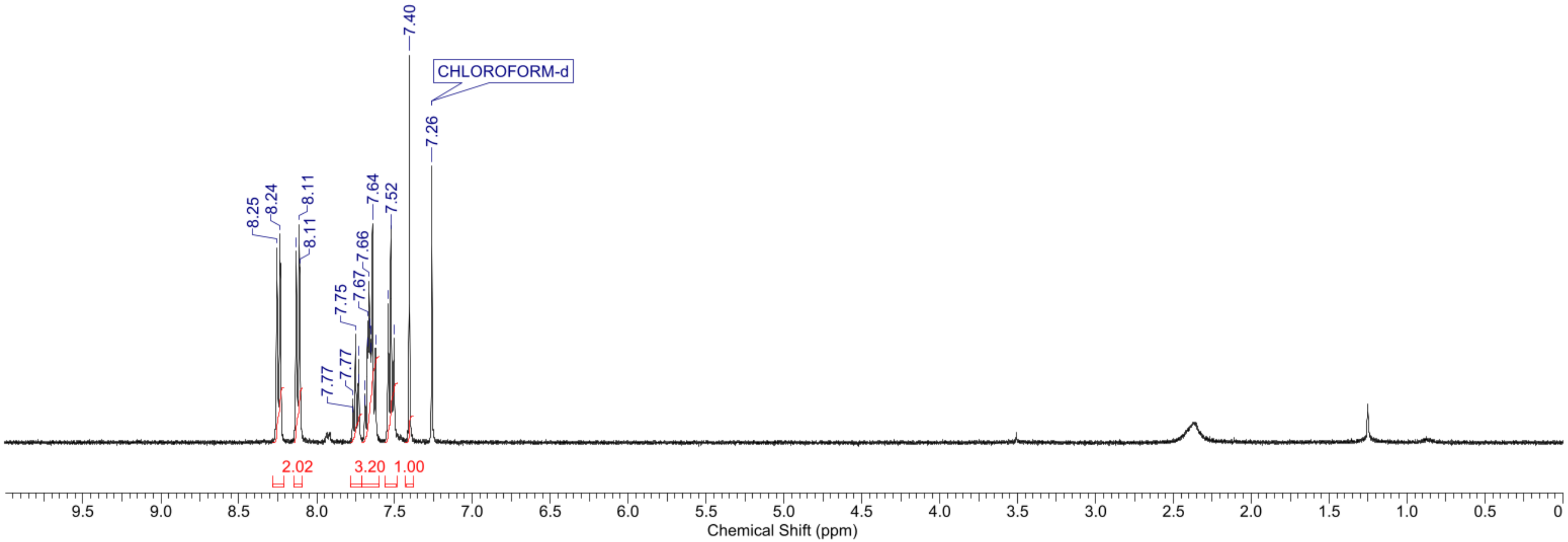

Phenyl(5-(phenylsulfonyl)isoxazol-3-yl)methanone **3e** (^{13}C NMR)

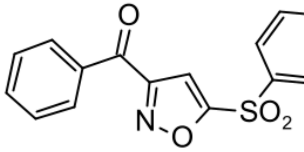

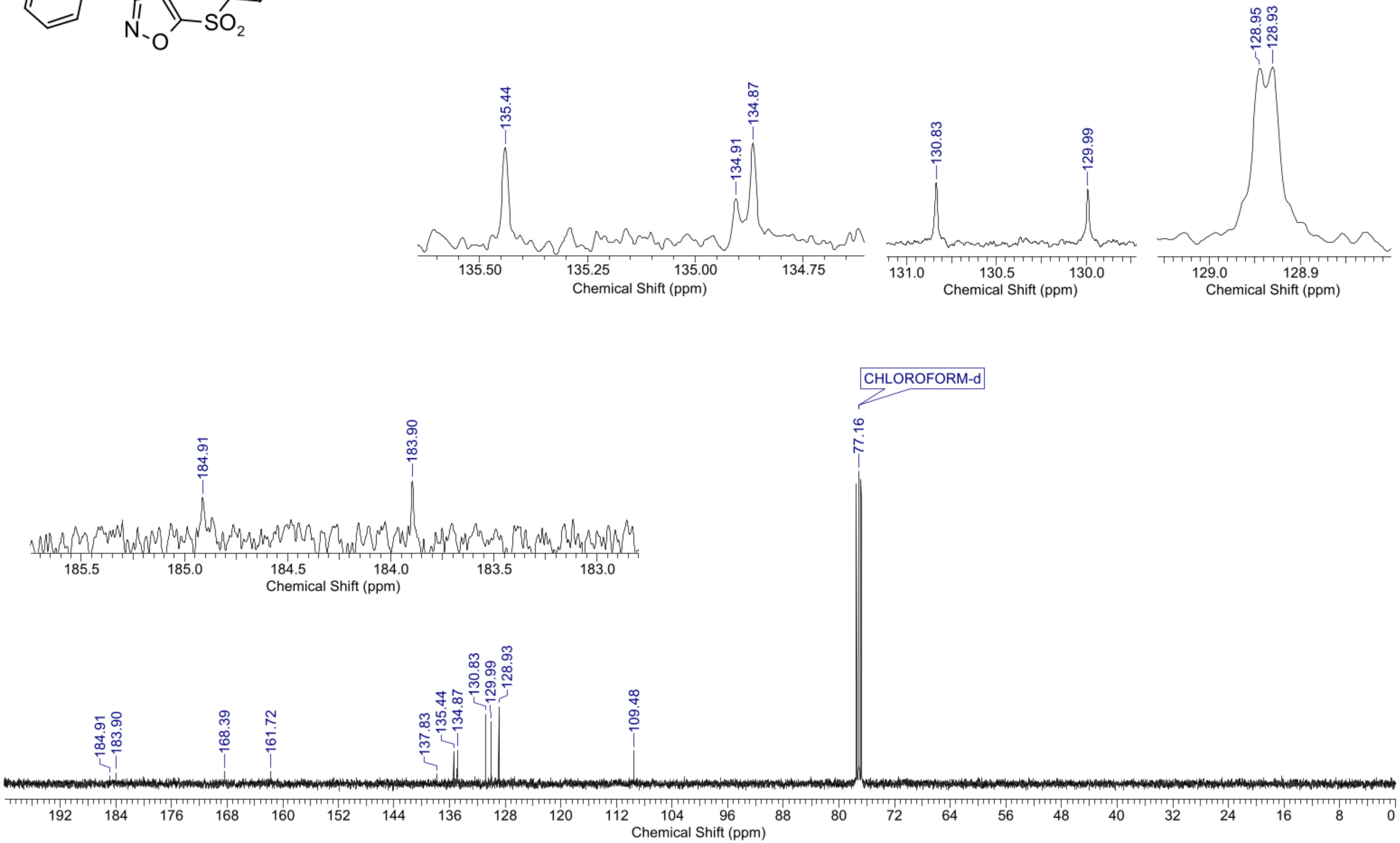

Diethyl (5-(phenylsulfonyl)isoxazol-3-yl)phosphonate **3f** (^{1}H NMR)

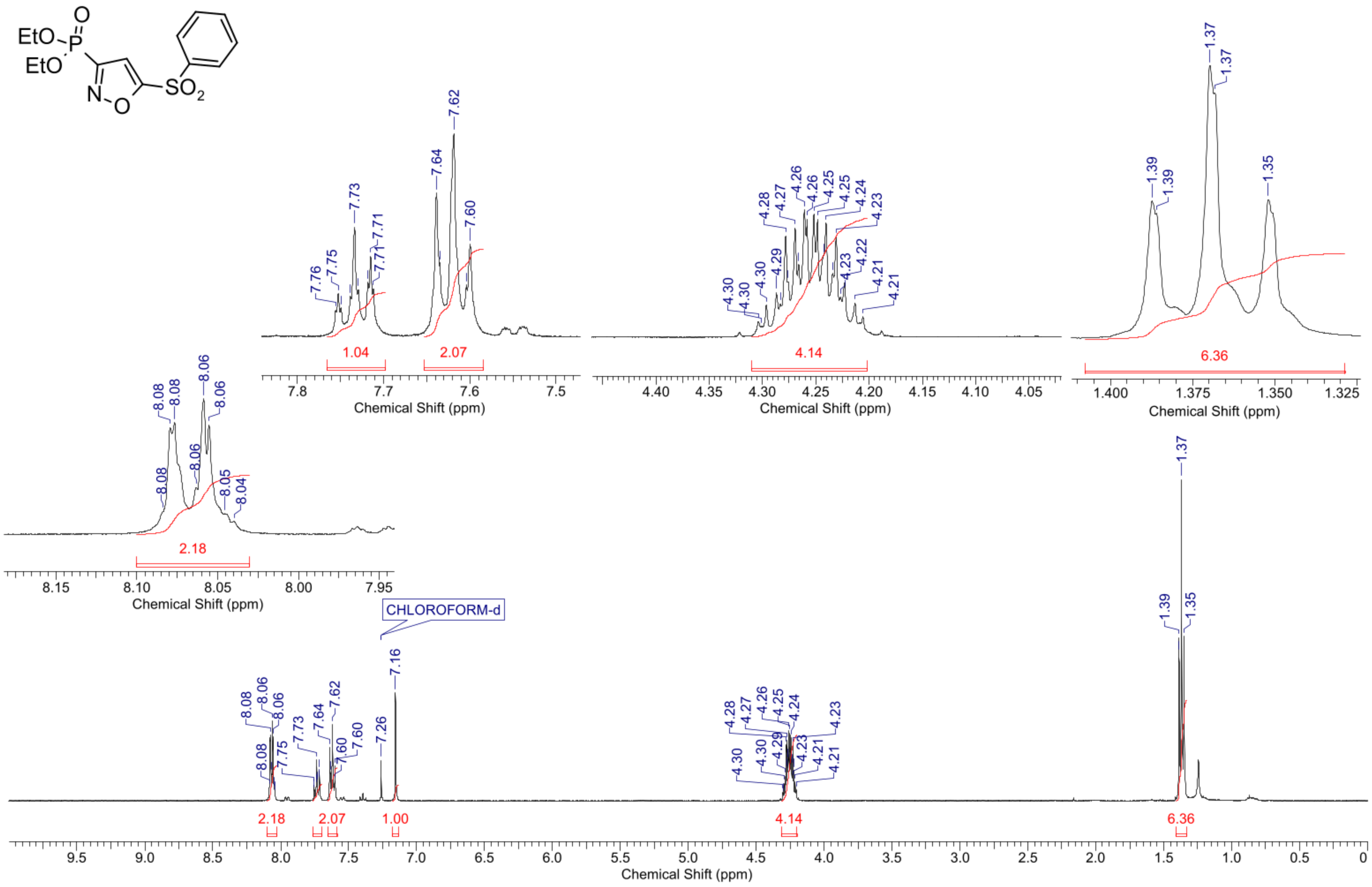

Diethyl (5-(phenylsulfonyl)isoxazol-3-yl)phosphonate **3f** (^{13}C NMR)

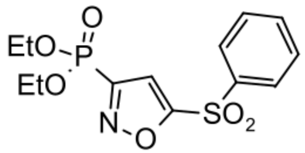

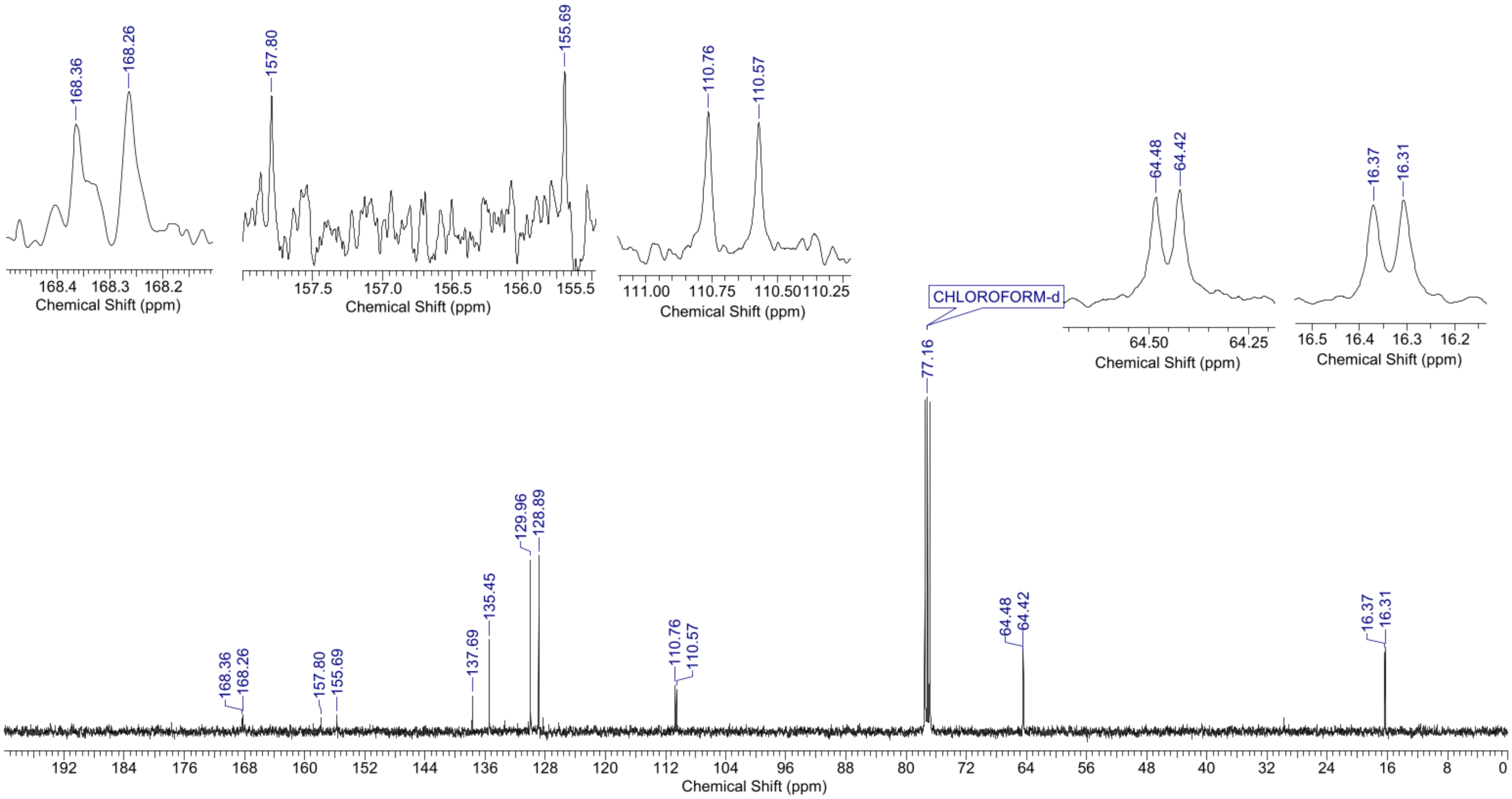

Diethyl (5-(phenylsulfonyl)isoxazol-3-yl)phosphonate **3f** (^{31}P NMR)

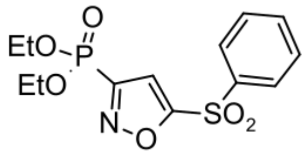

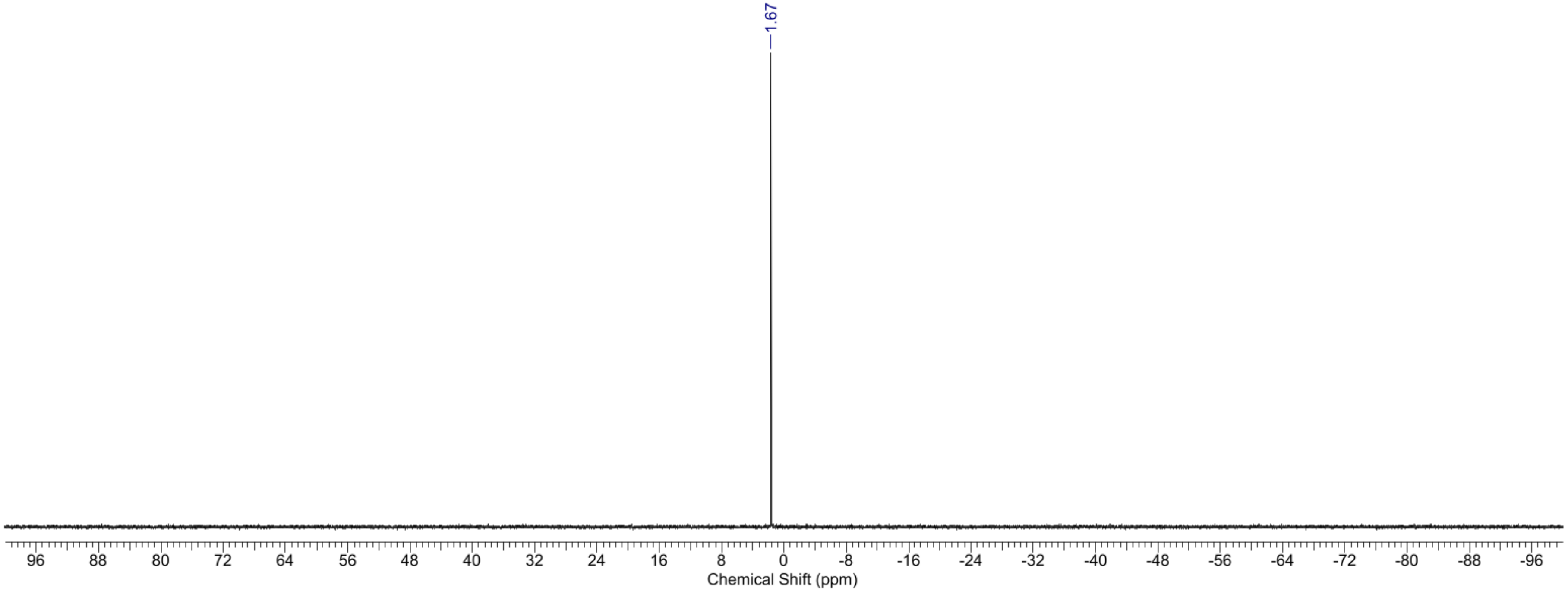

3-Nitro-5-(phenylsulfonyl)isoxazole **3g** (^{1}H NMR)

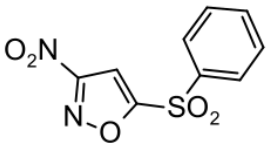

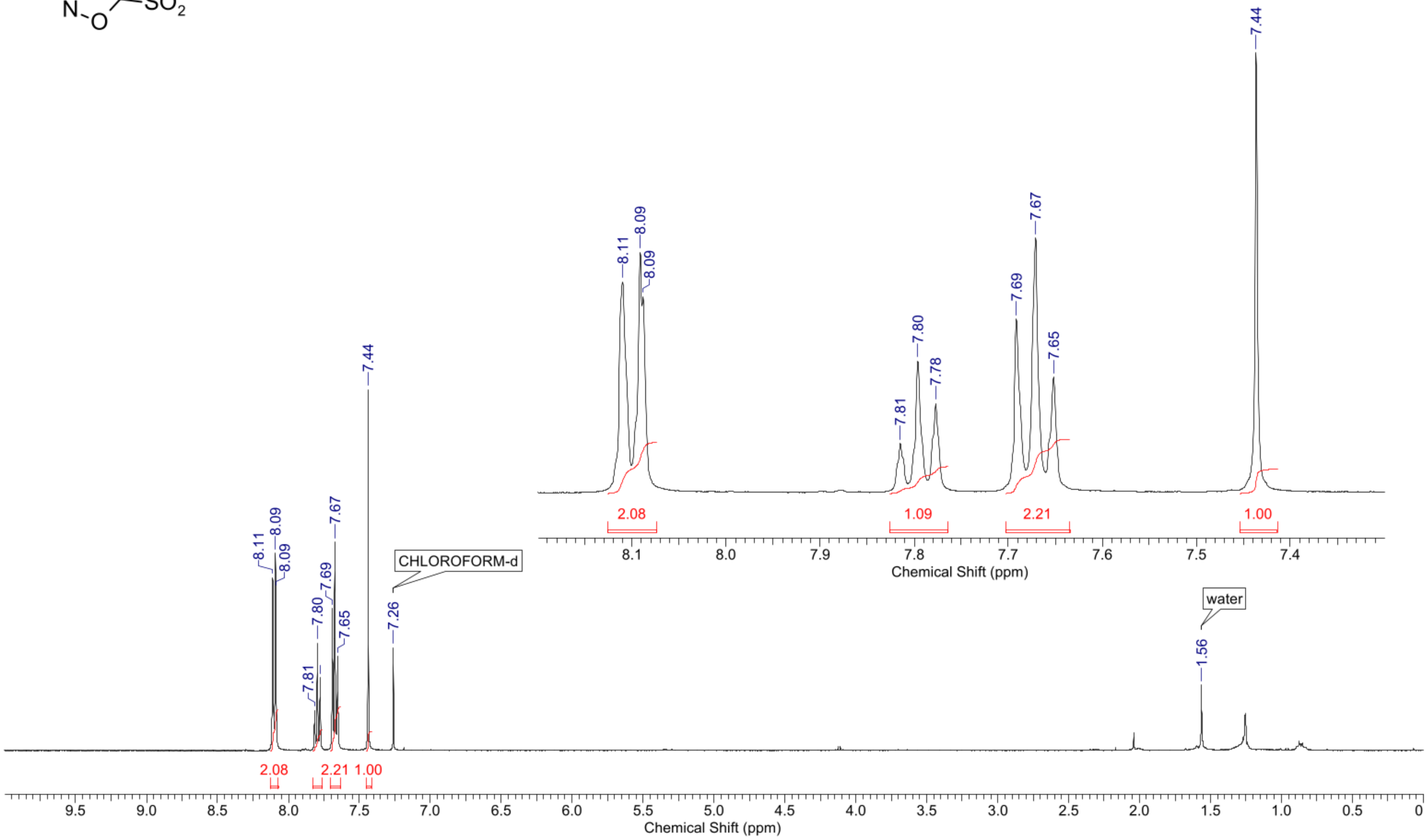

3-Nitro-5-(phenylsulfonyl)isoxazole **3g** (^{13}C NMR)

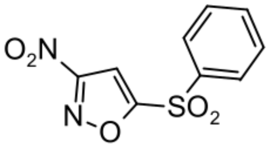

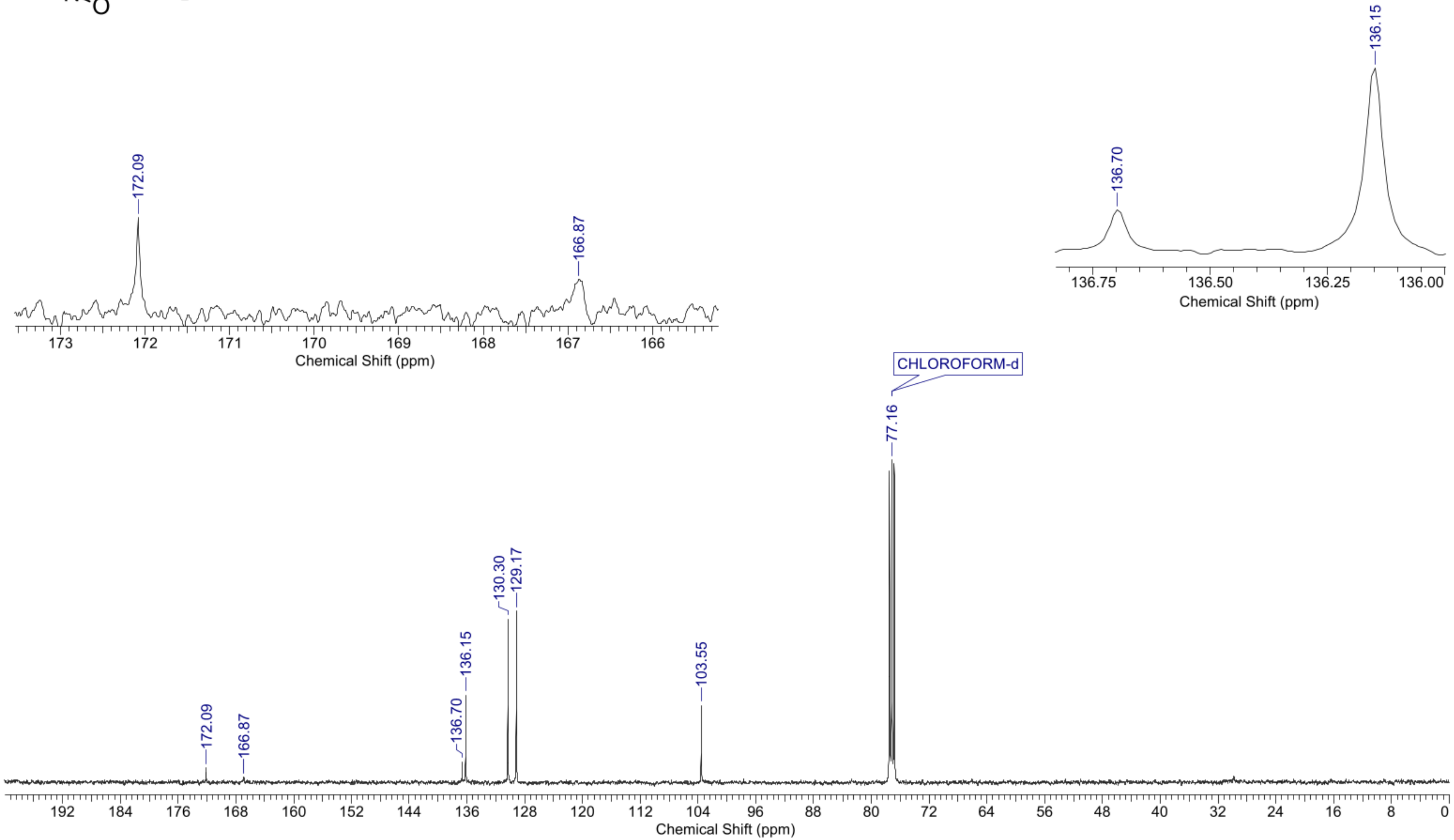

5-((4-Chlorophenyl)sulfonyl)-3-nitroisoxazole **3h** (^{1}H NMR)

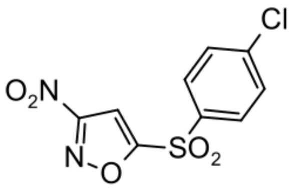

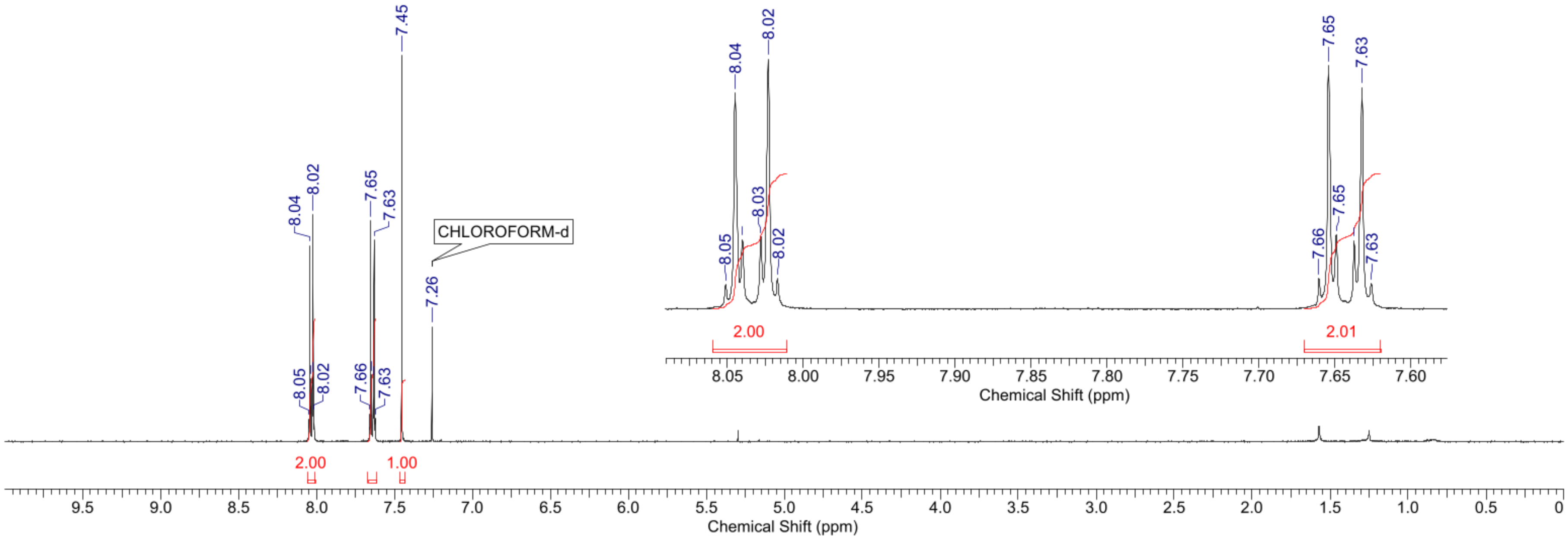

5-((4-Chlorophenyl)sulfonyl)-3-nitroisoxazole **3h** (^{13}C NMR)

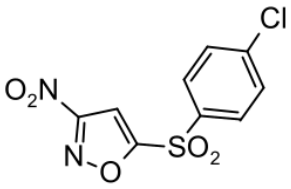

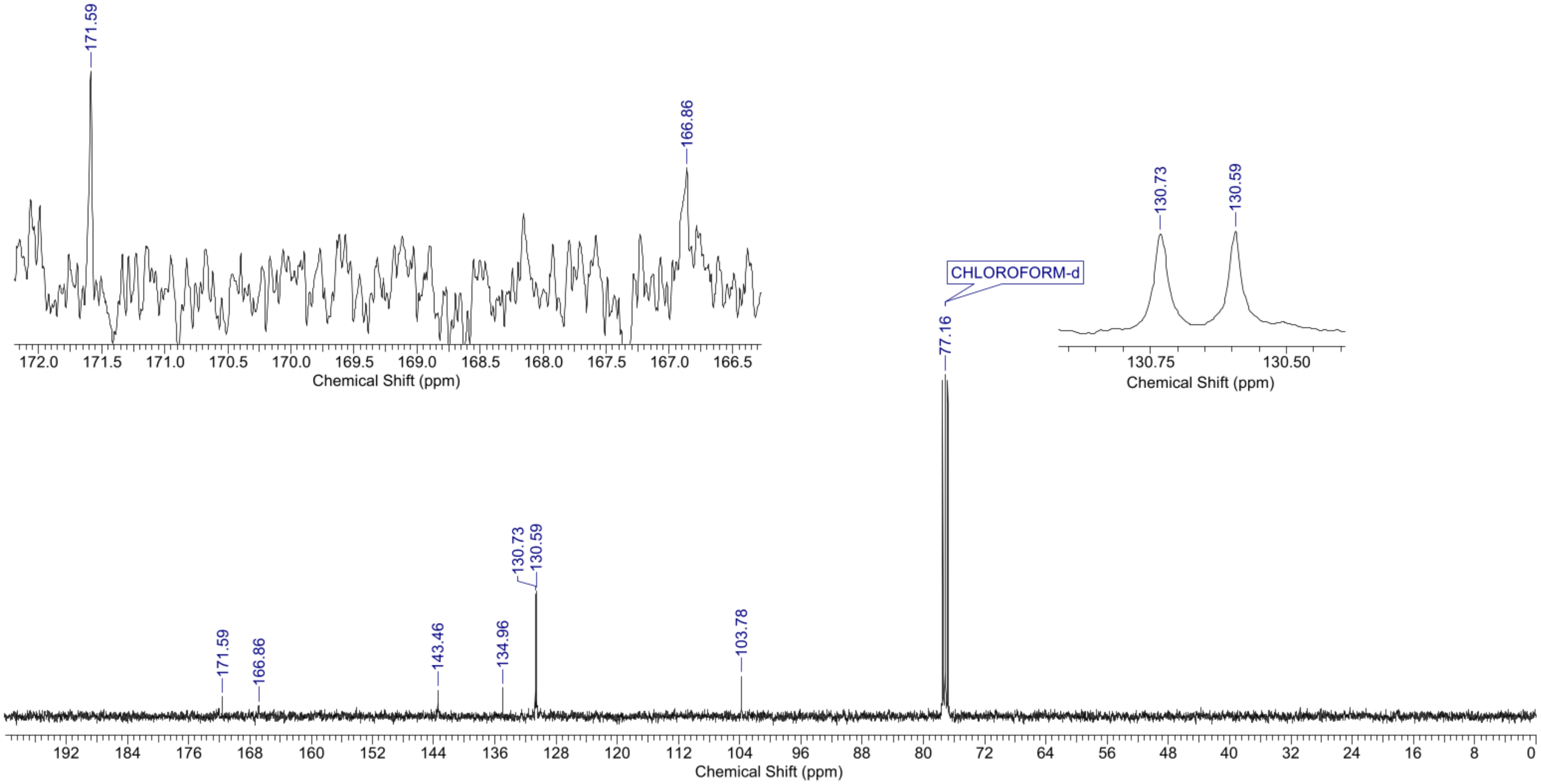

5-((4-Fluorophenyl)sulfonyl)-3-nitroisoxazole **3i** (^{1}H NMR)

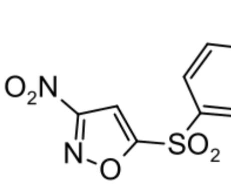

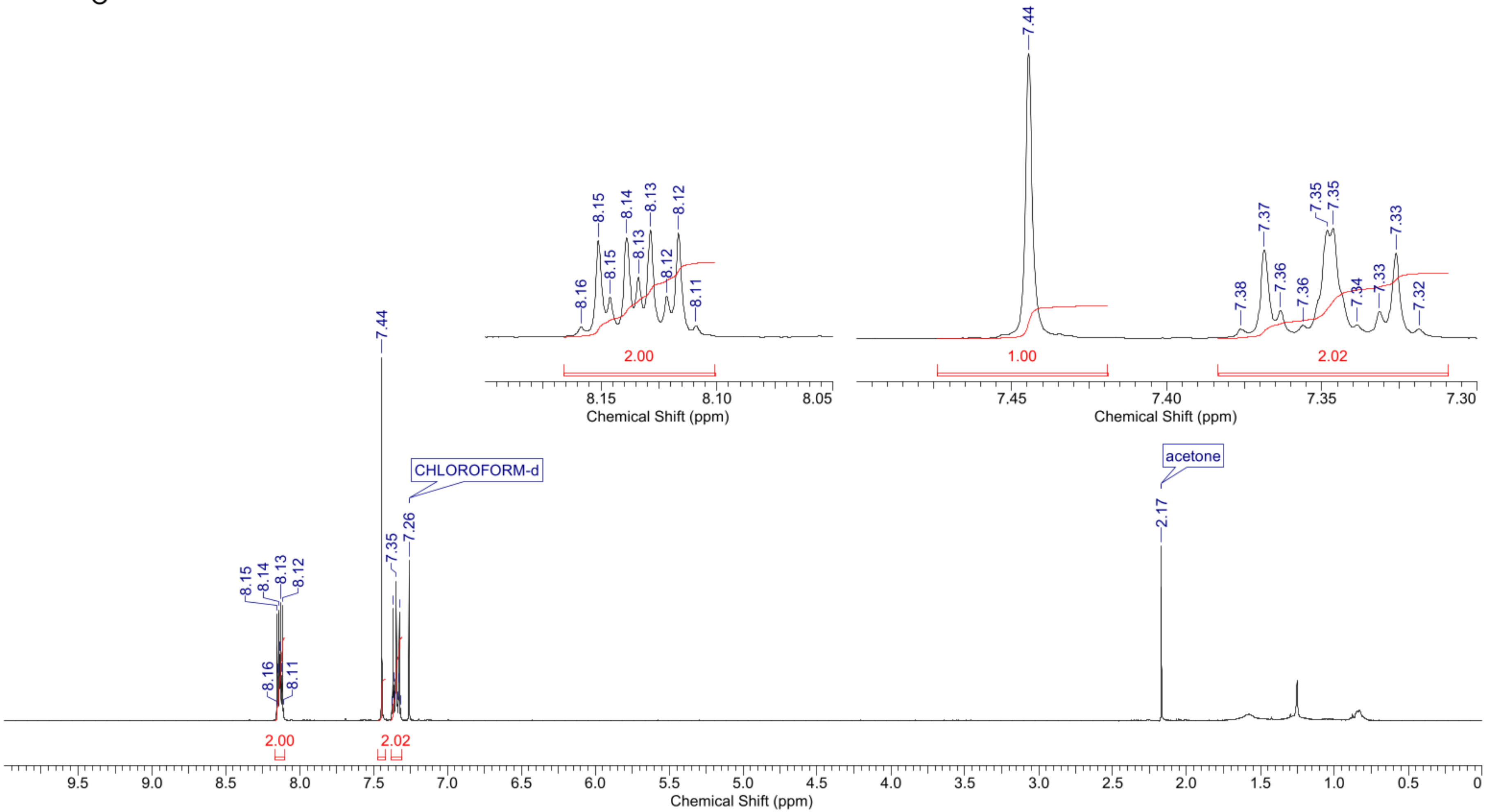

5-((4-Fluorophenyl)sulfonyl)-3-nitroisoxazole **3i** (^{13}C NMR)

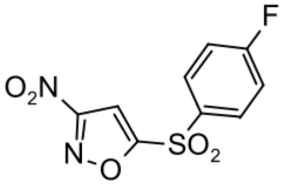

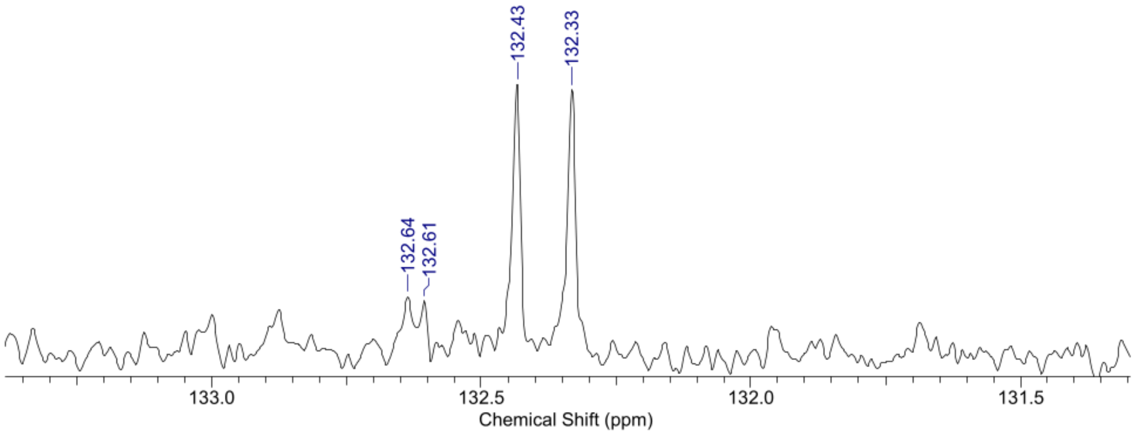

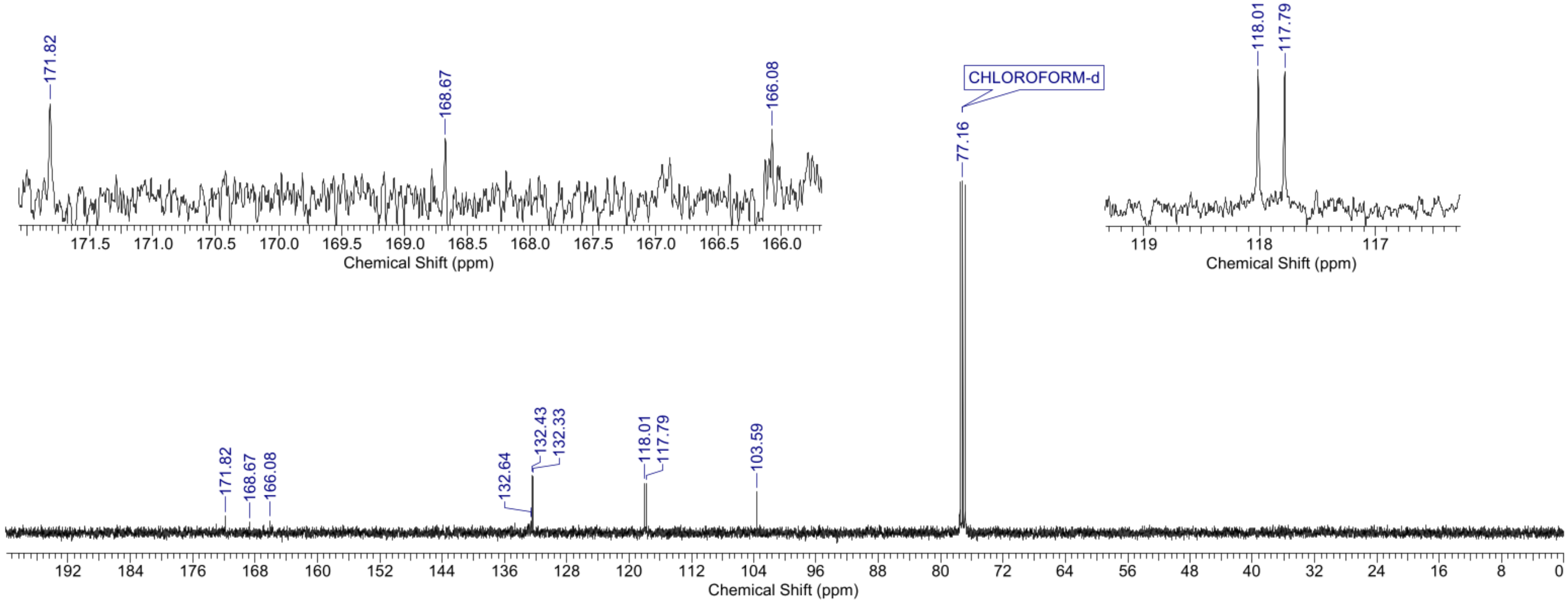

5-((4-Fluorophenyl)sulfonyl)-3-nitroisoxazole **3i** (^{19}F NMR)

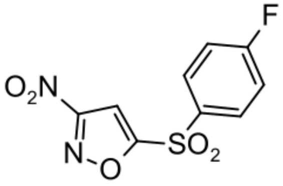

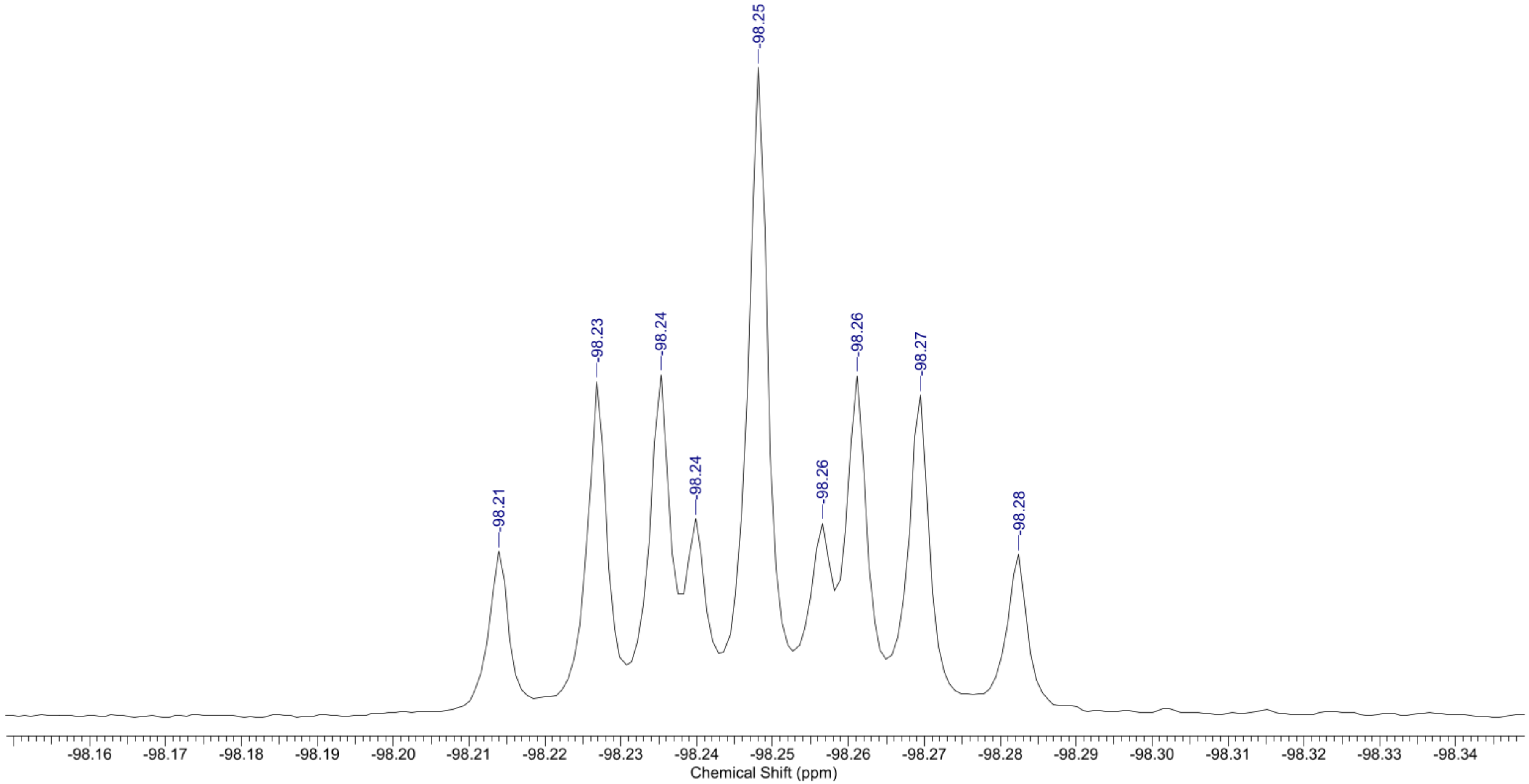

3-Nitro-5-tosylisoxazole **3j** (^{1}H NMR)

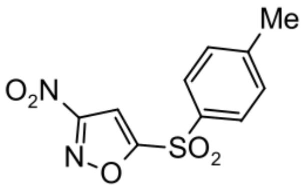

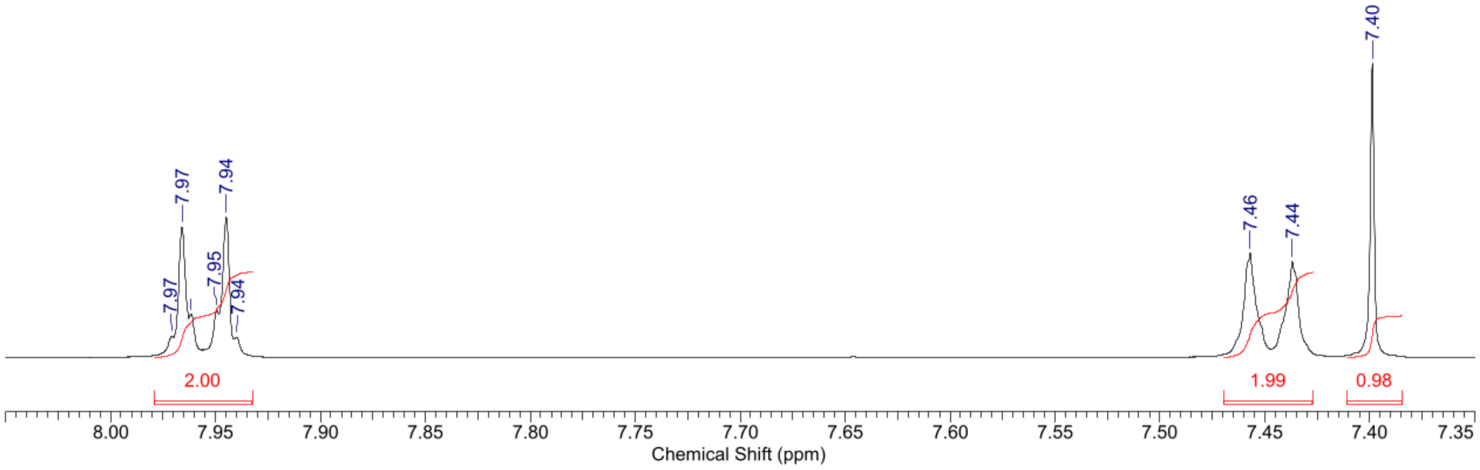

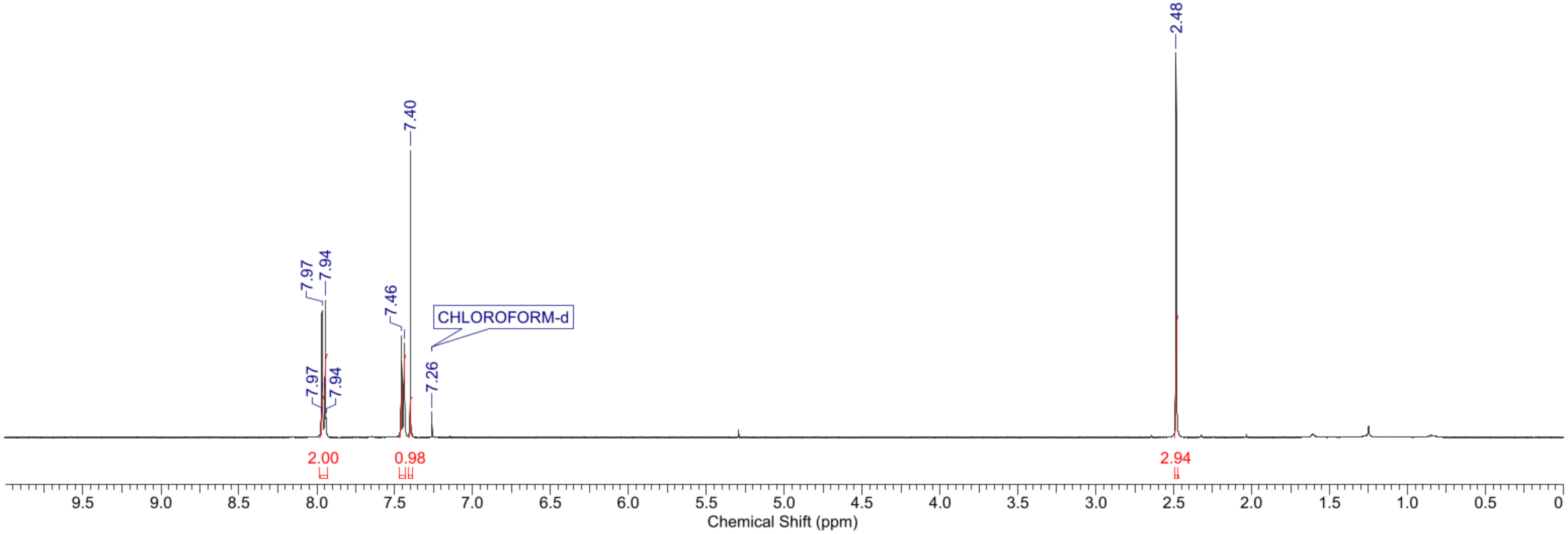

3-Nitro-5-tosylisoxazole **3j** (^{13}C NMR)

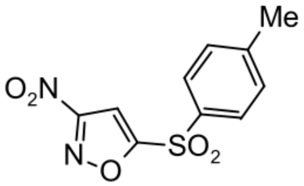

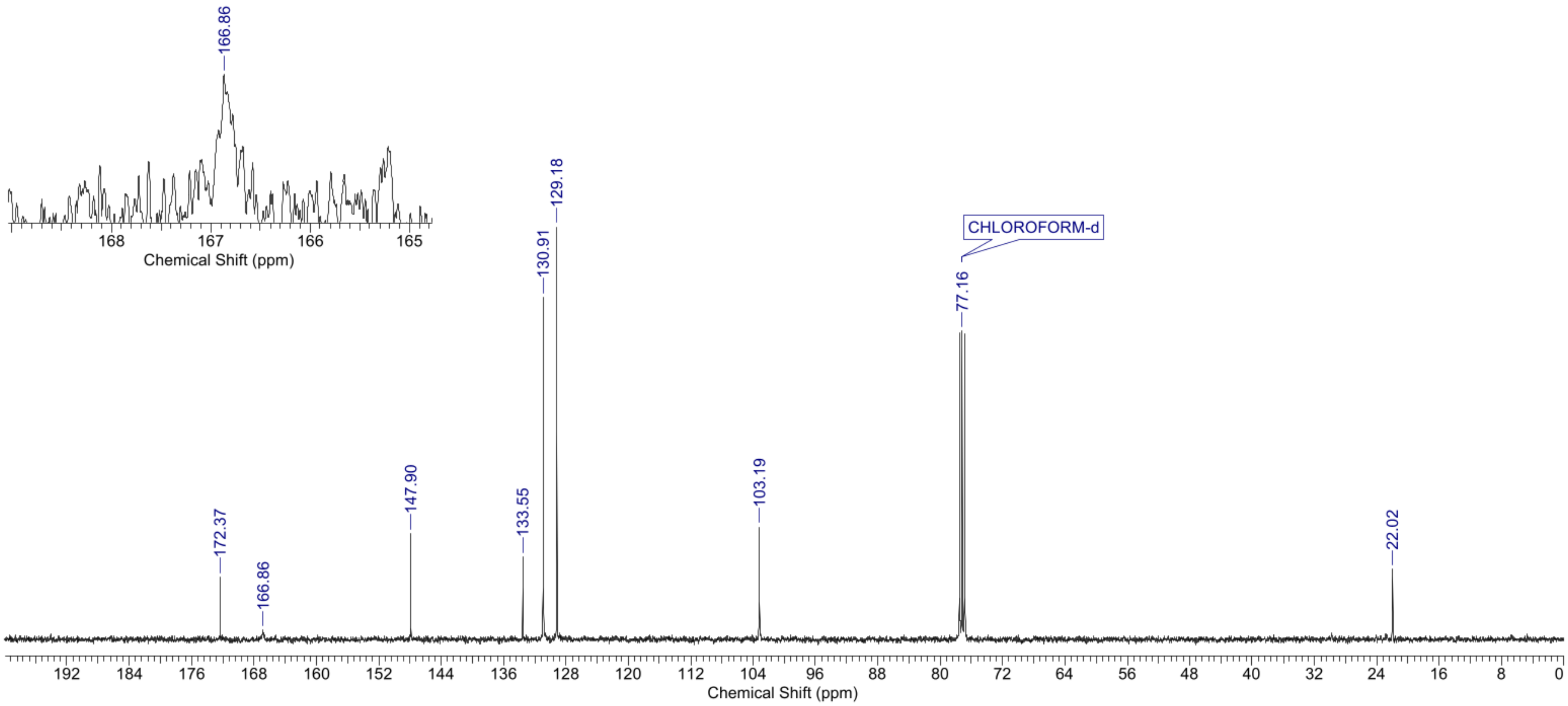

5-((4-Methoxyphenyl)sulfonyl)-3-nitroisoxazole **3k** (^{1}H NMR)

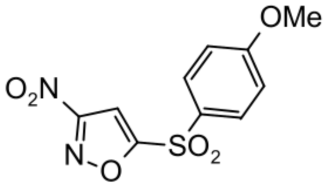

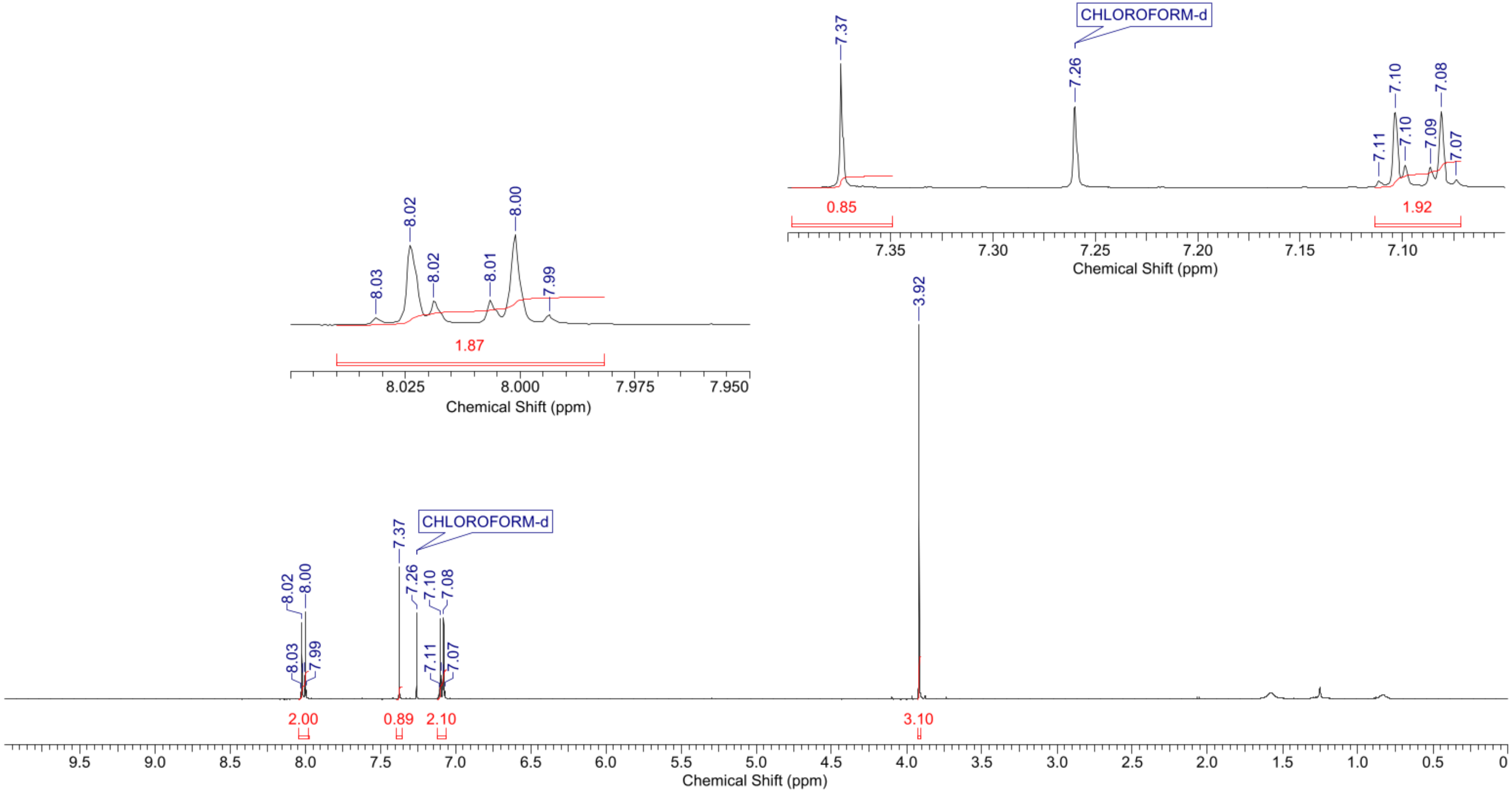

5-((4-Methoxyphenyl)sulfonyl)-3-nitroisoxazole **3k** (^{13}C NMR)

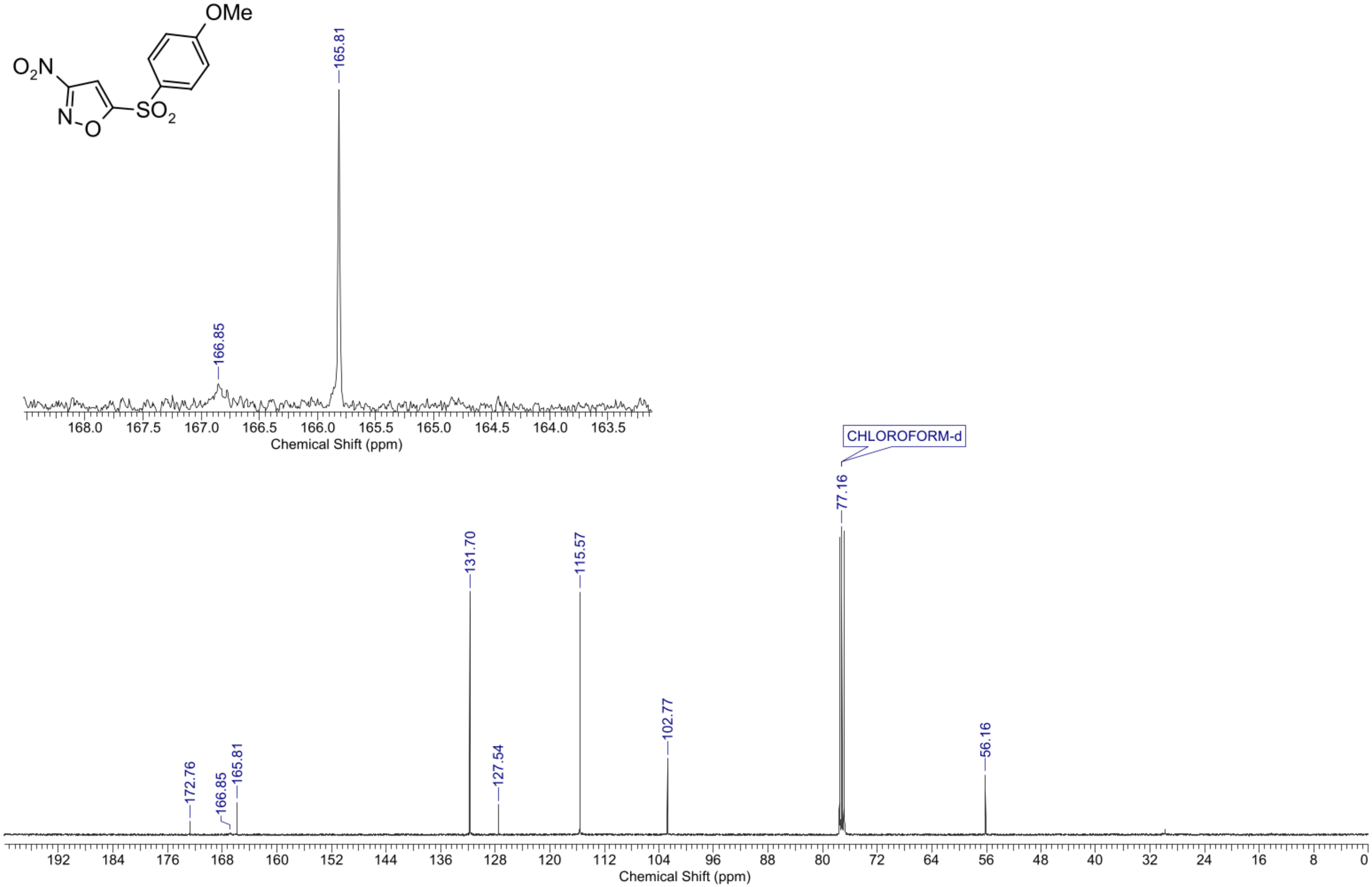

Methyl 5-((3,4-dimethylphenyl)sulfonyl)isoxazole-3-carboxylate **3l** (^{1}H NMR)

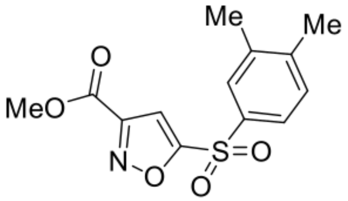

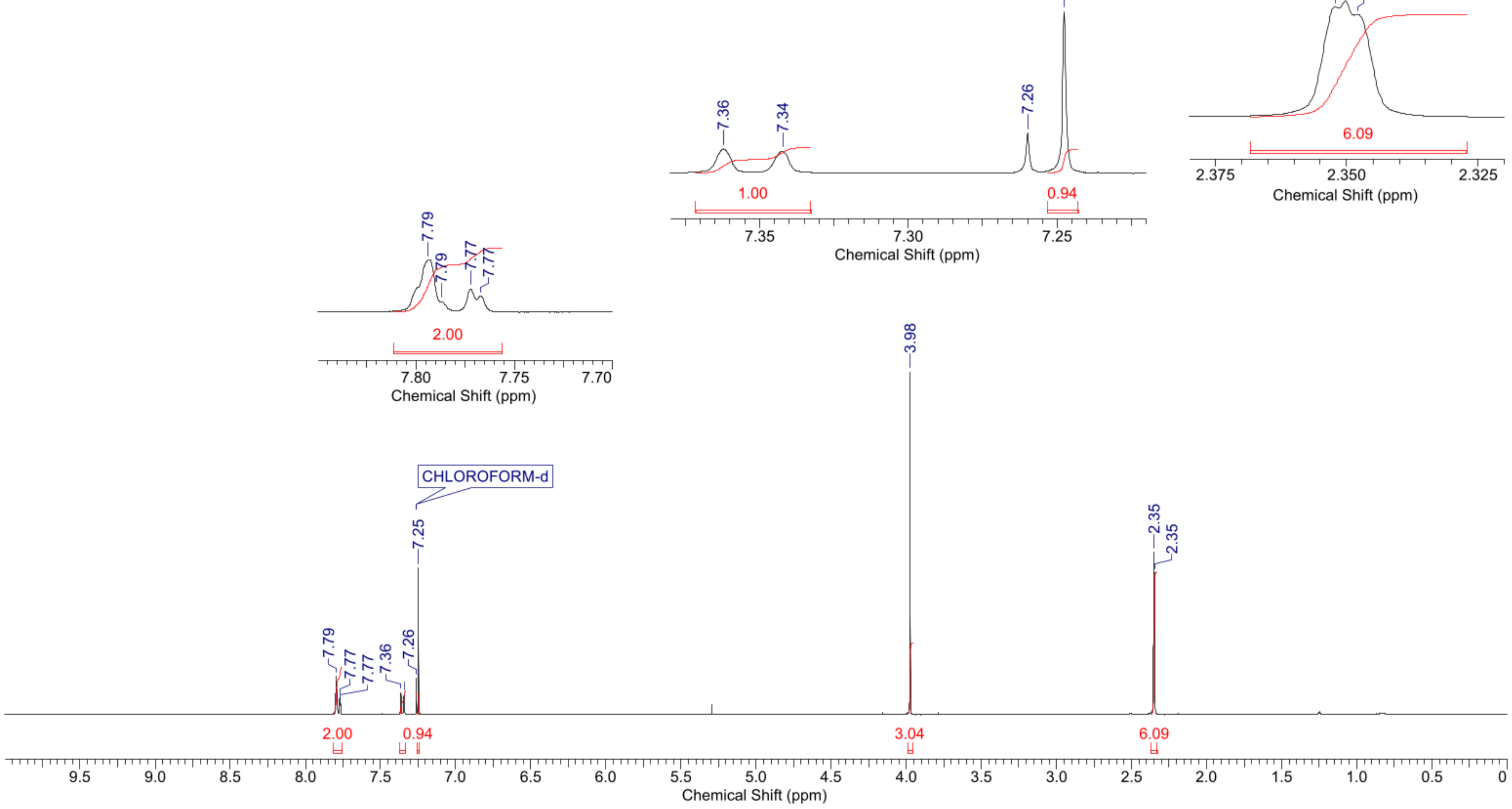

Methyl 5-((3,4-dimethylphenyl)sulfonyl)isoxazole-3-carboxylate **3l** (^{13}C NMR)

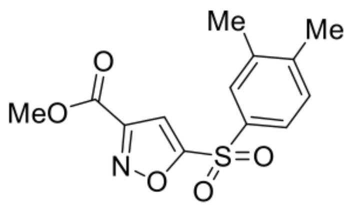

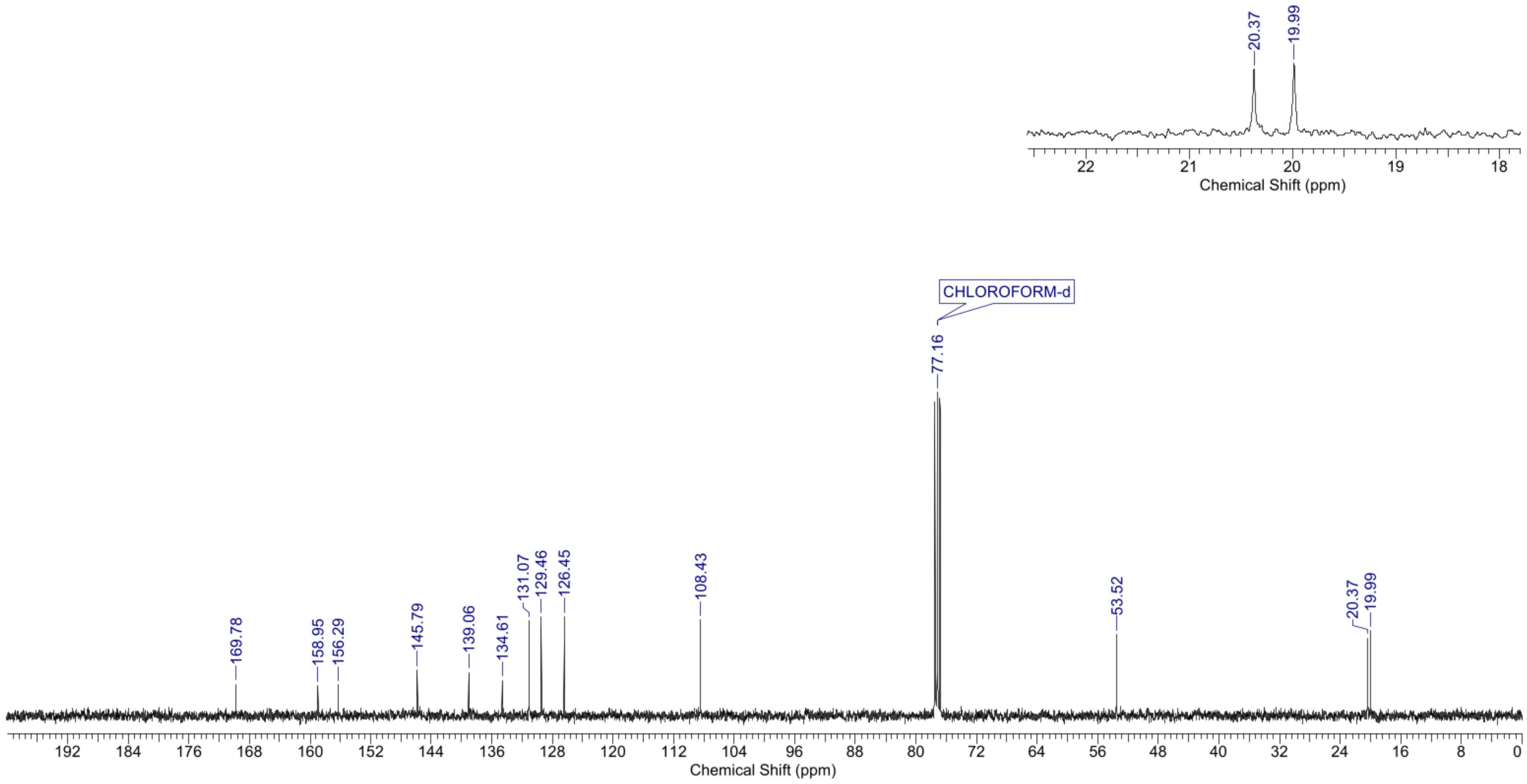

Methyl 5-((4-chlorophenyl)sulfonyl)isoxazole-3-carboxylate **3m** (^{1}H NMR)

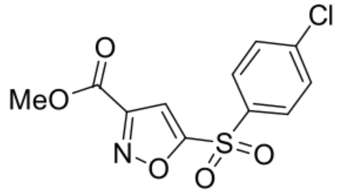

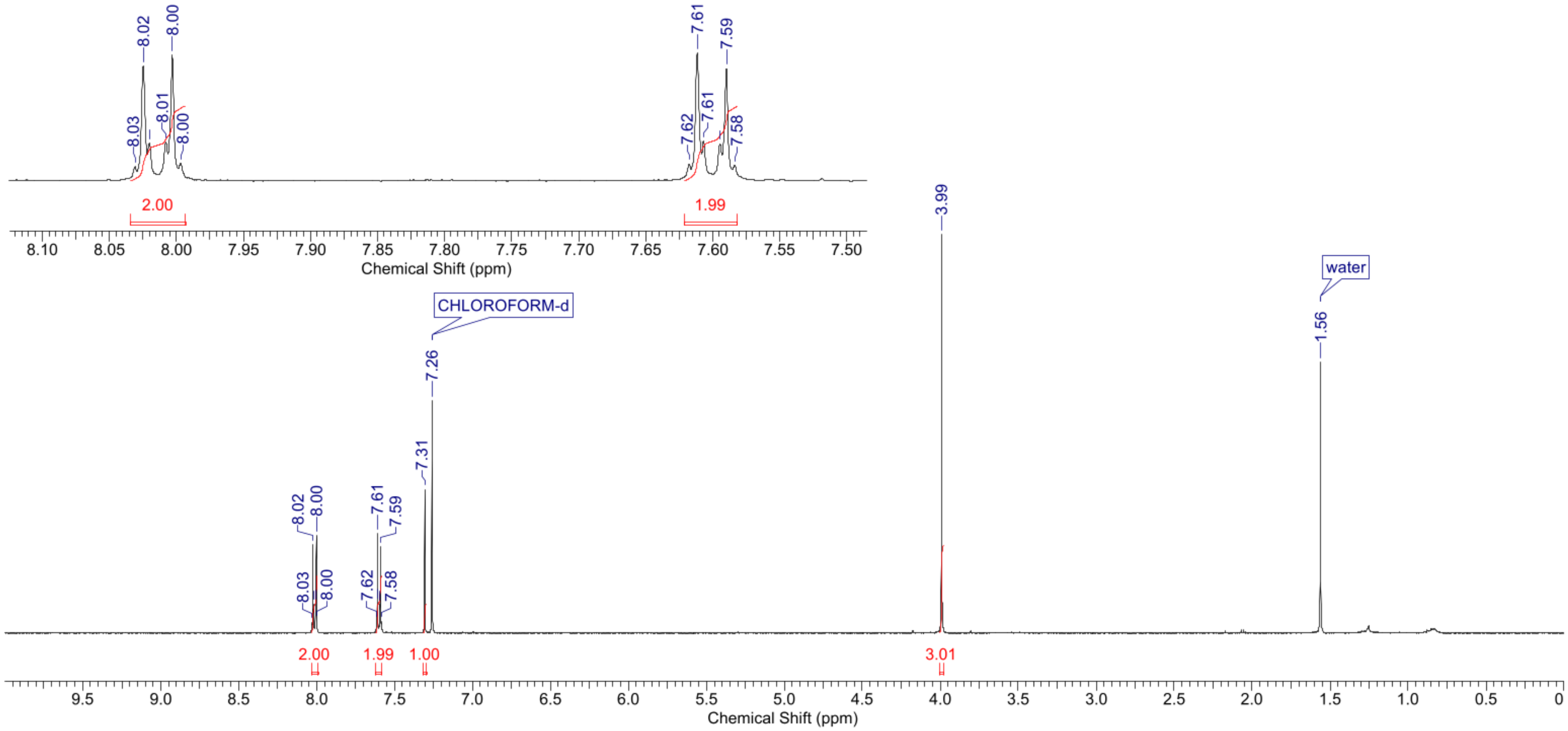

Methyl 5-((4-chlorophenyl)sulfonyl)isoxazole-3-carboxylate **3m** (^{13}C NMR)

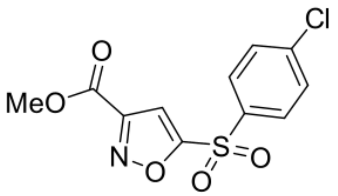

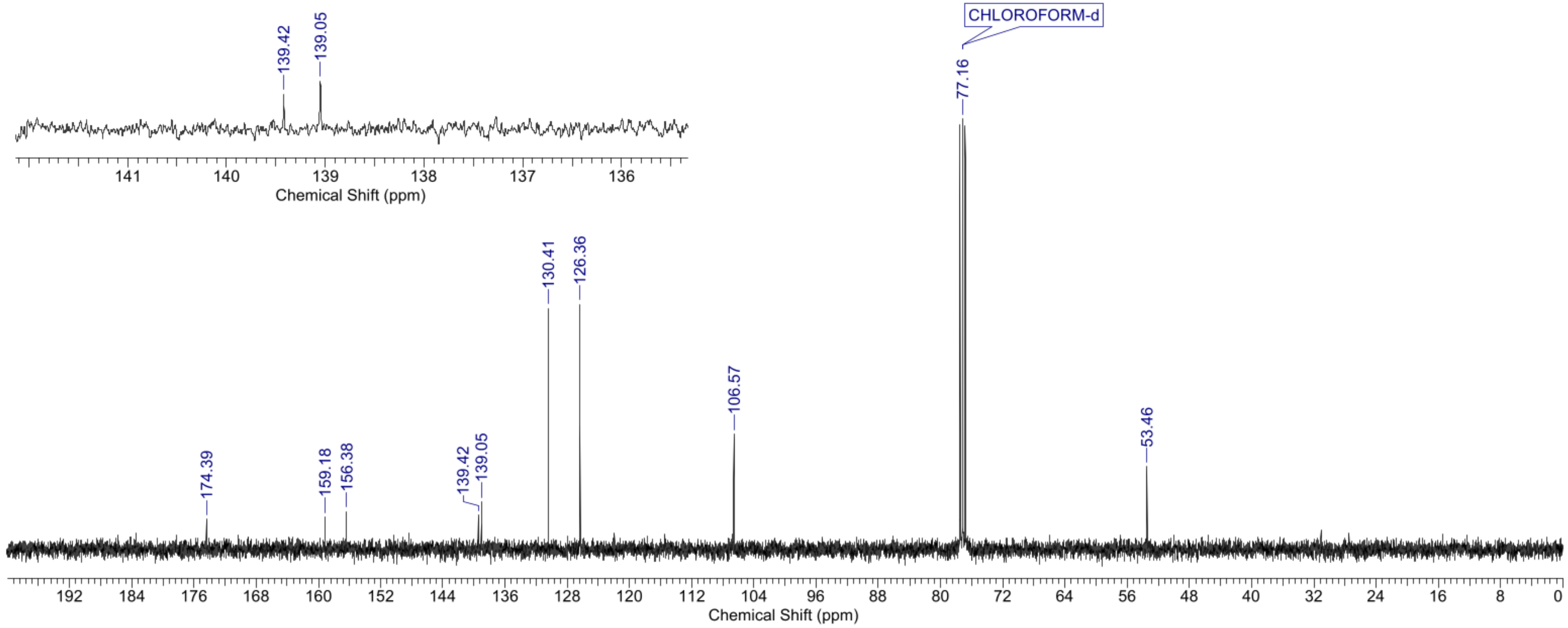

Methyl 5-((4-fluorophenyl)sulfonyl)isoxazole-3-carboxylate **3n** (^{1}H NMR)

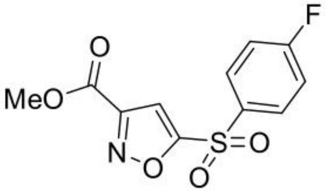

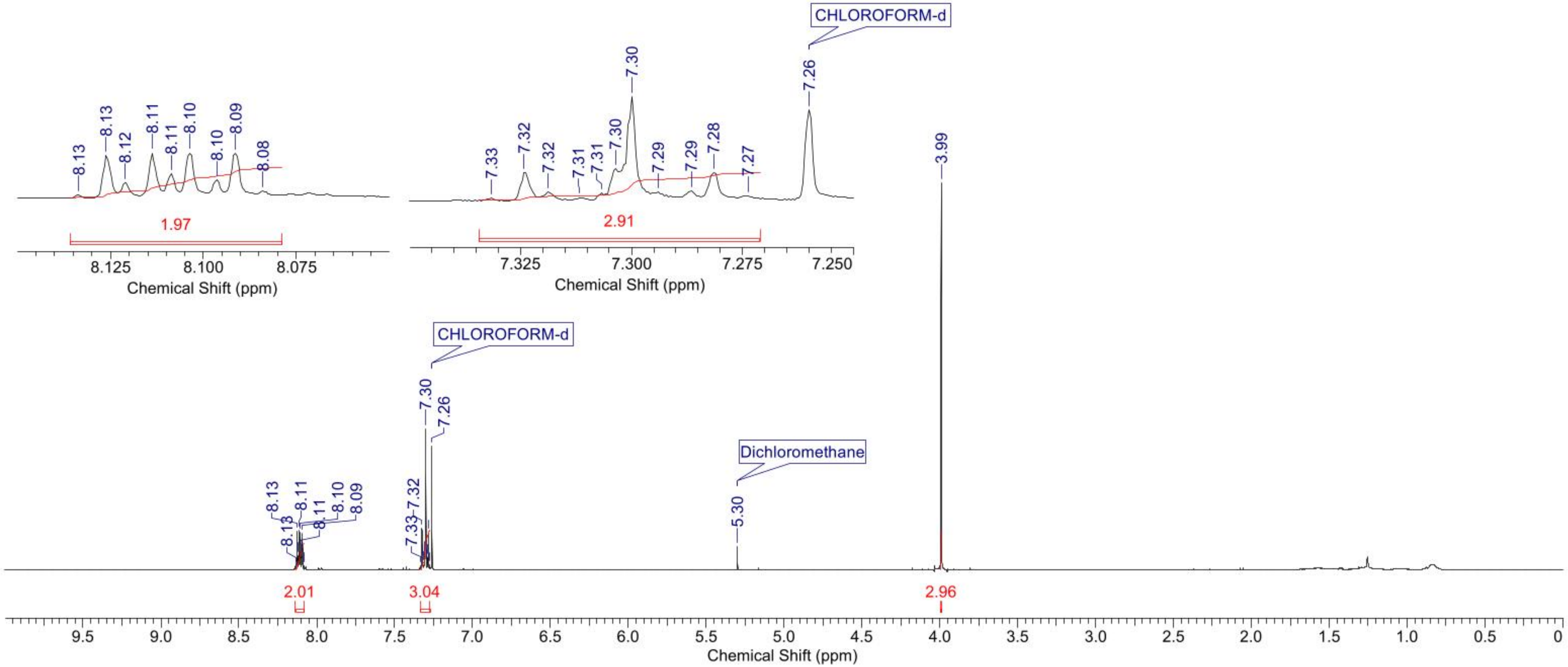

Methyl 5-((4-fluorophenyl)sulfonyl)isoxazole-3-carboxylate **3n** (^{13}C NMR)

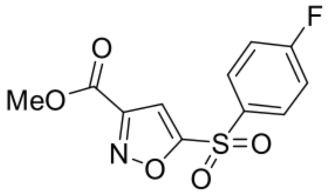

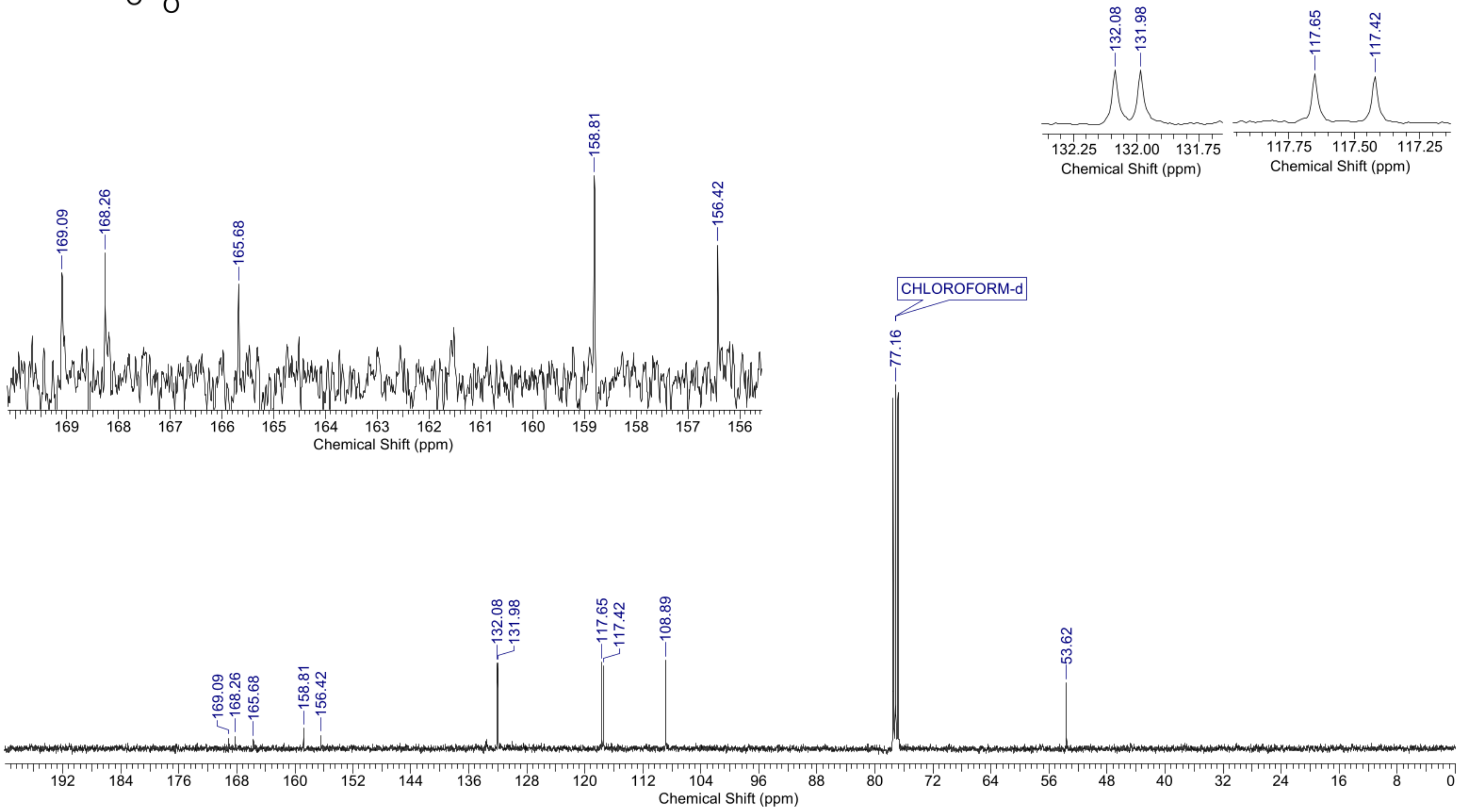

Methyl 5-((4-fluorophenyl)sulfonyl)isoxazole-3-carboxylate **3n** (^{19}F NMR)

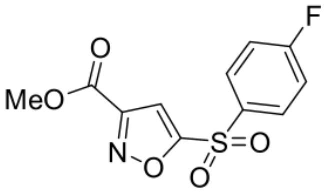

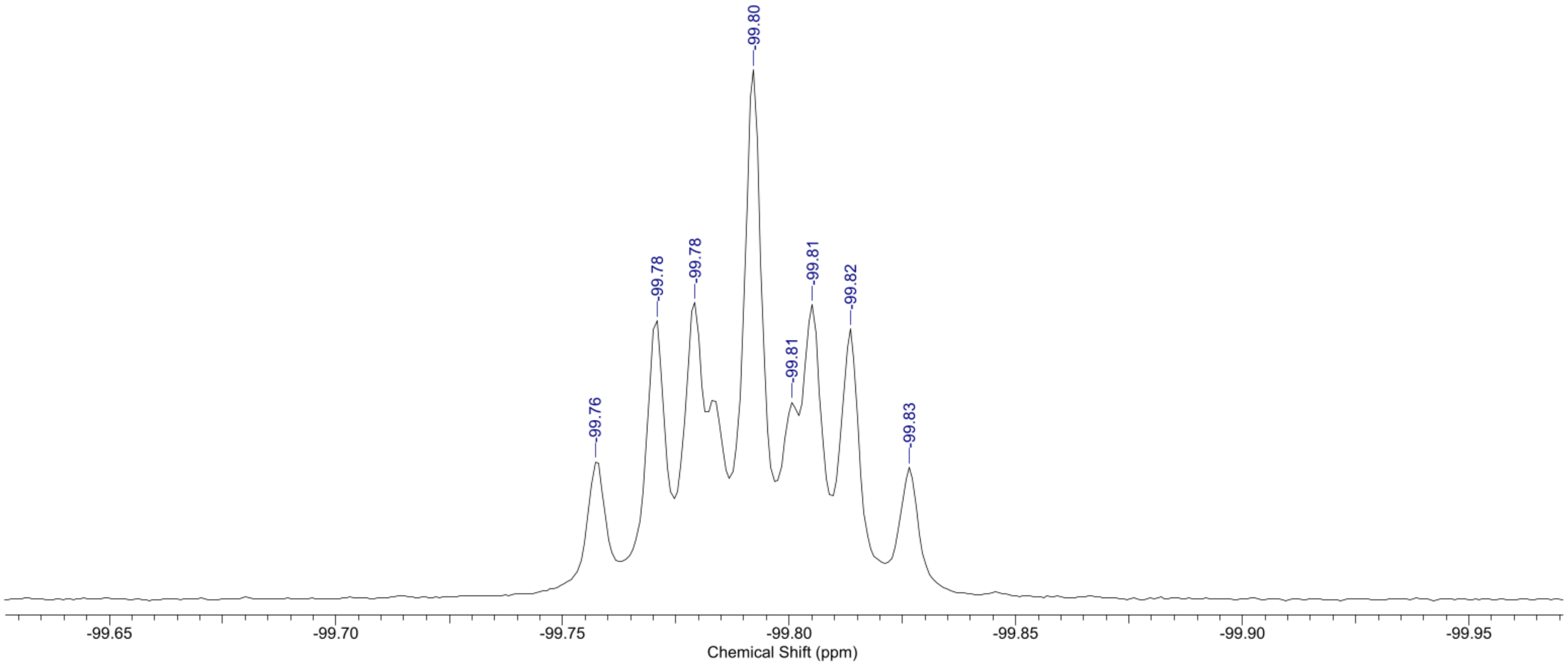

Methyl 5-((4-fluorophenyl)sulfonyl)isoxazole-3-carboxylate **3n** (HMBC)

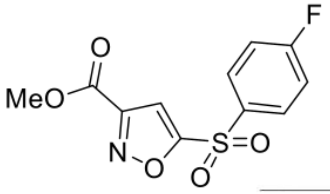

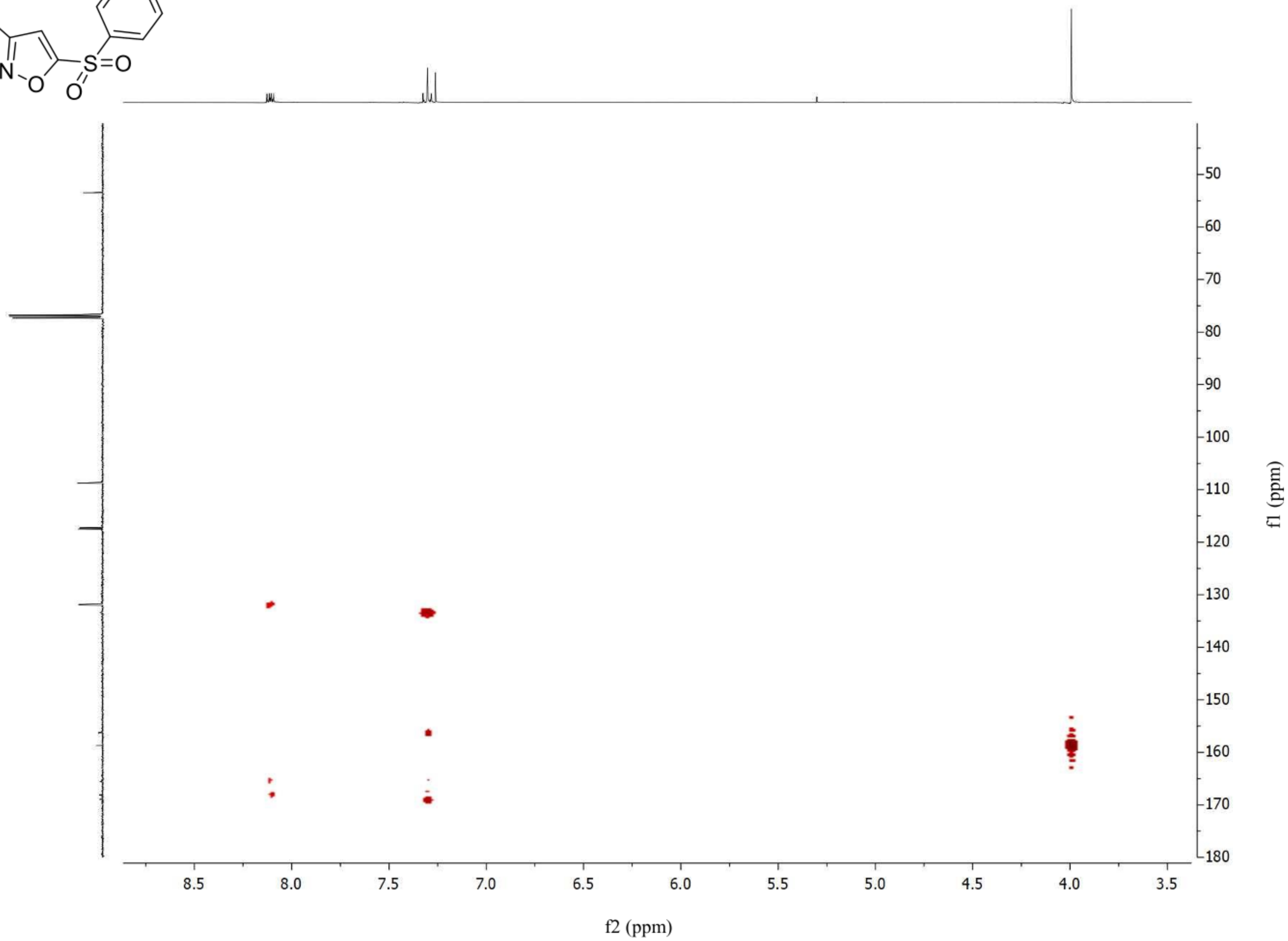

Methyl 5-tosylisoxazole-3-carboxylate **3o** (^{1}H NMR)

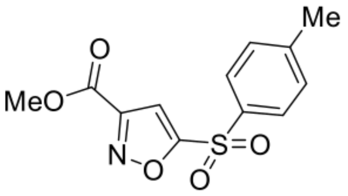

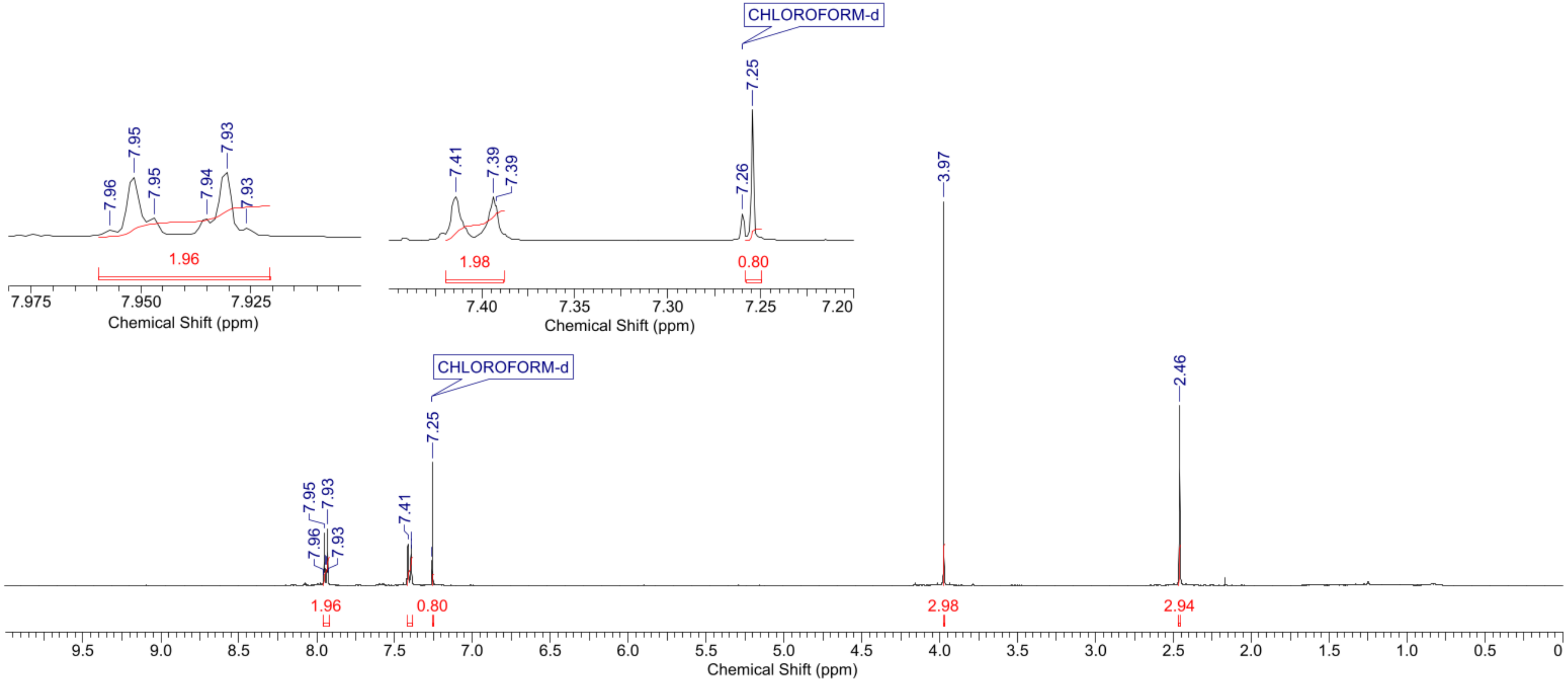

Methyl 5-tosylisoxazole-3-carboxylate **3o** (^{13}C NMR)

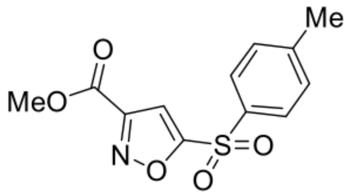

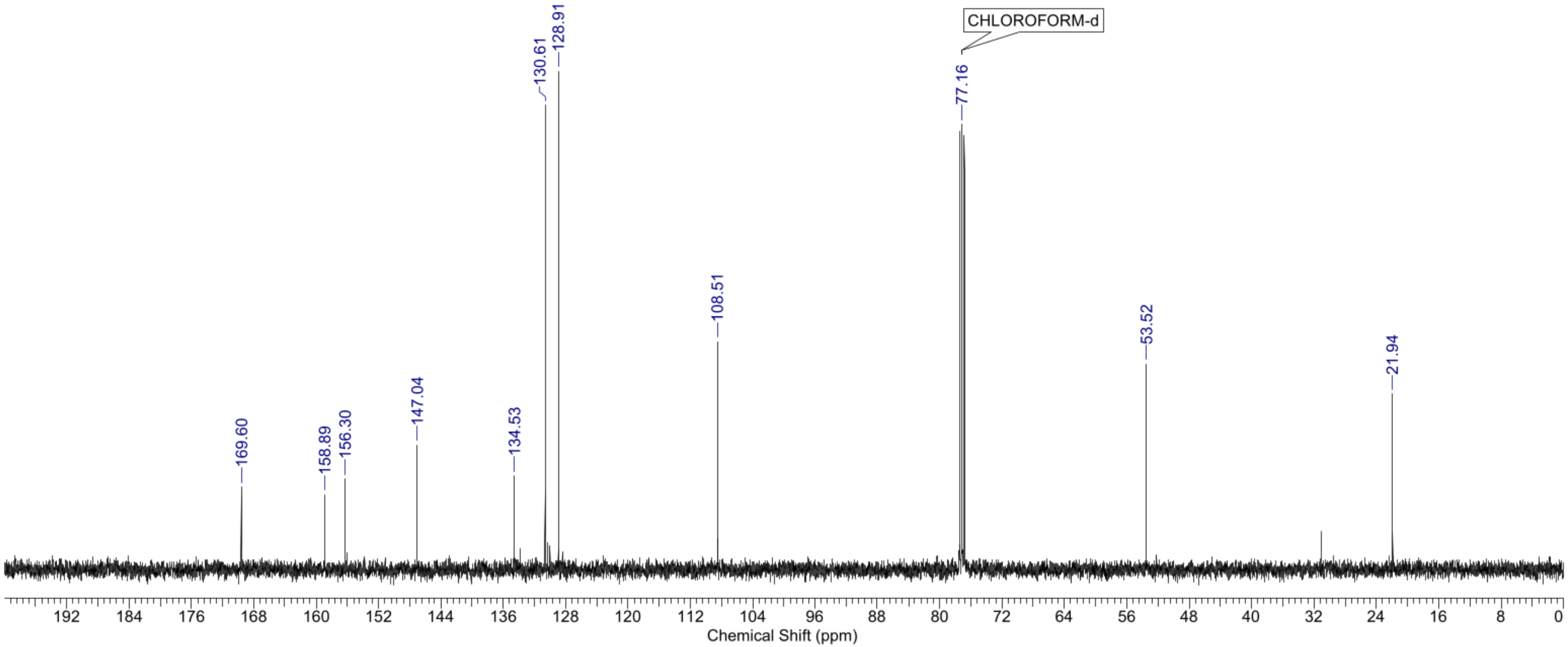

Methyl 5-((4-methoxyphenyl)sulfonyl)isoxazole-3-carboxylate **3p** (^{1}H NMR)

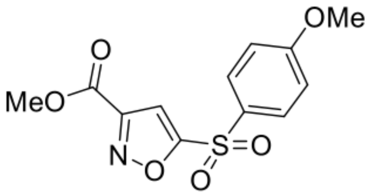

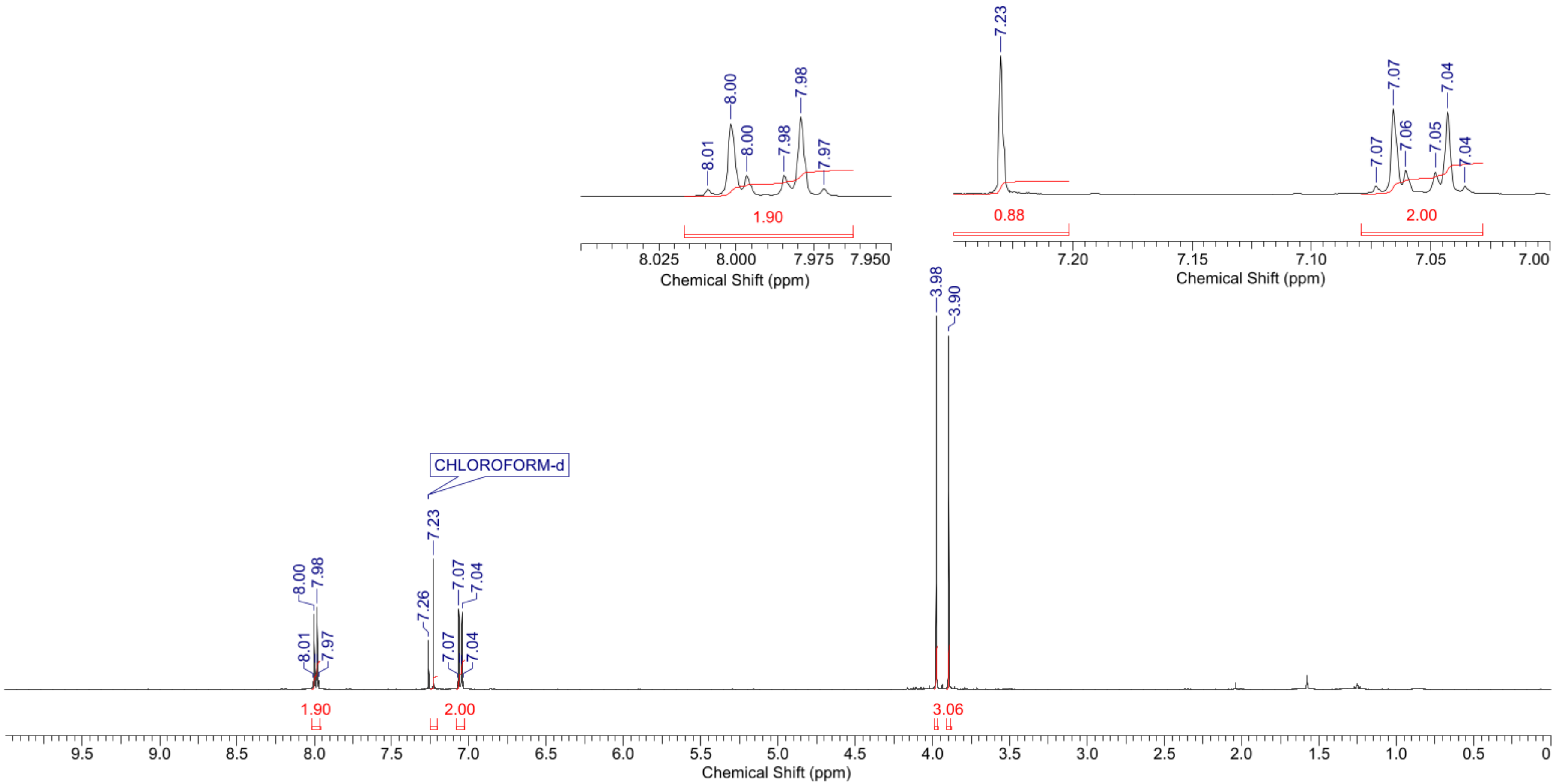

Methyl 5-((4-methoxyphenyl)sulfonyl)isoxazole-3-carboxylate **3p** (^{13}C NMR)

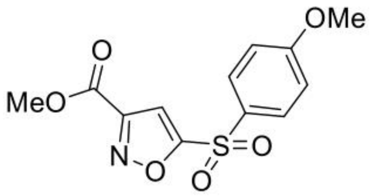

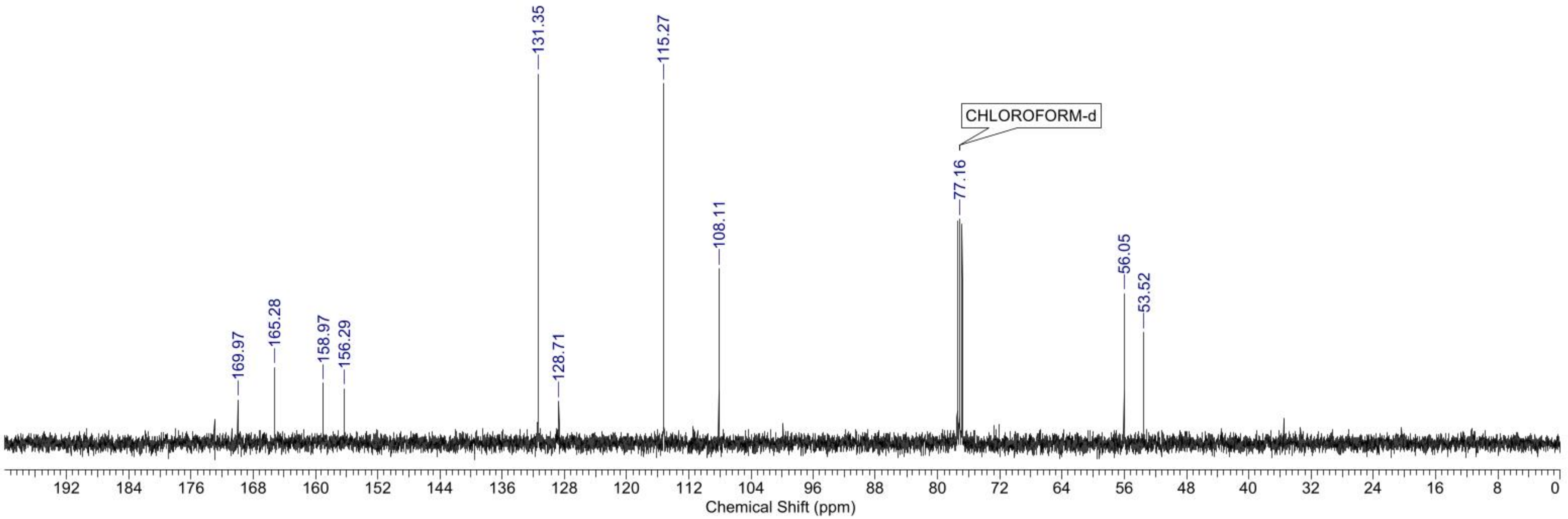

Methyl 5-((4-methoxyphenyl)sulfonyl)isoxazole-3-carboxylate **3p** (HSQC)

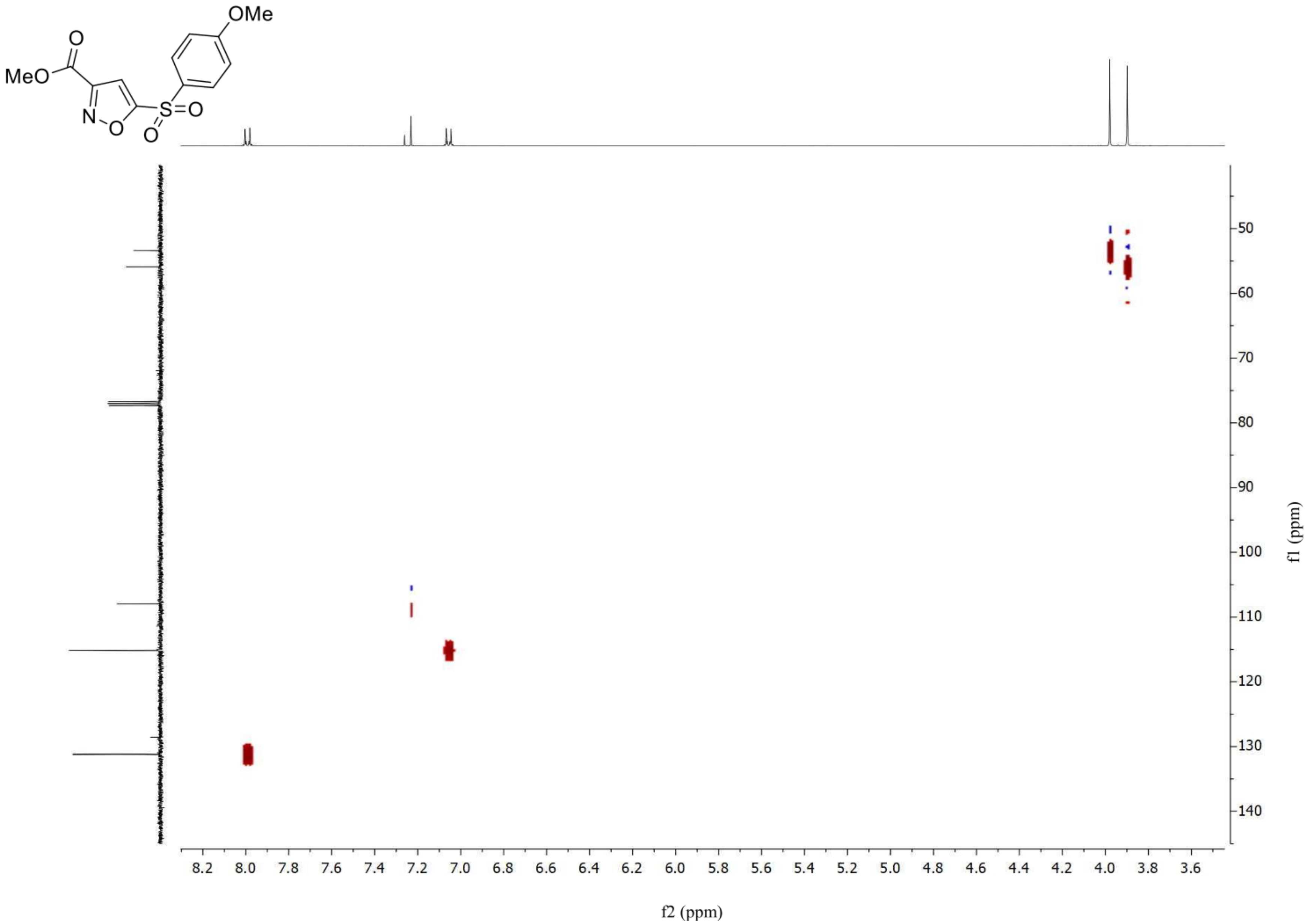

Methyl 5-((4-methoxyphenyl)sulfonyl)isoxazole-3-carboxylate **3p** (HMBC)

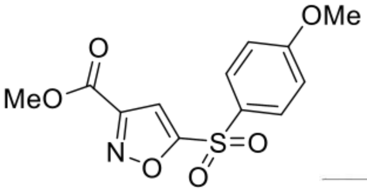

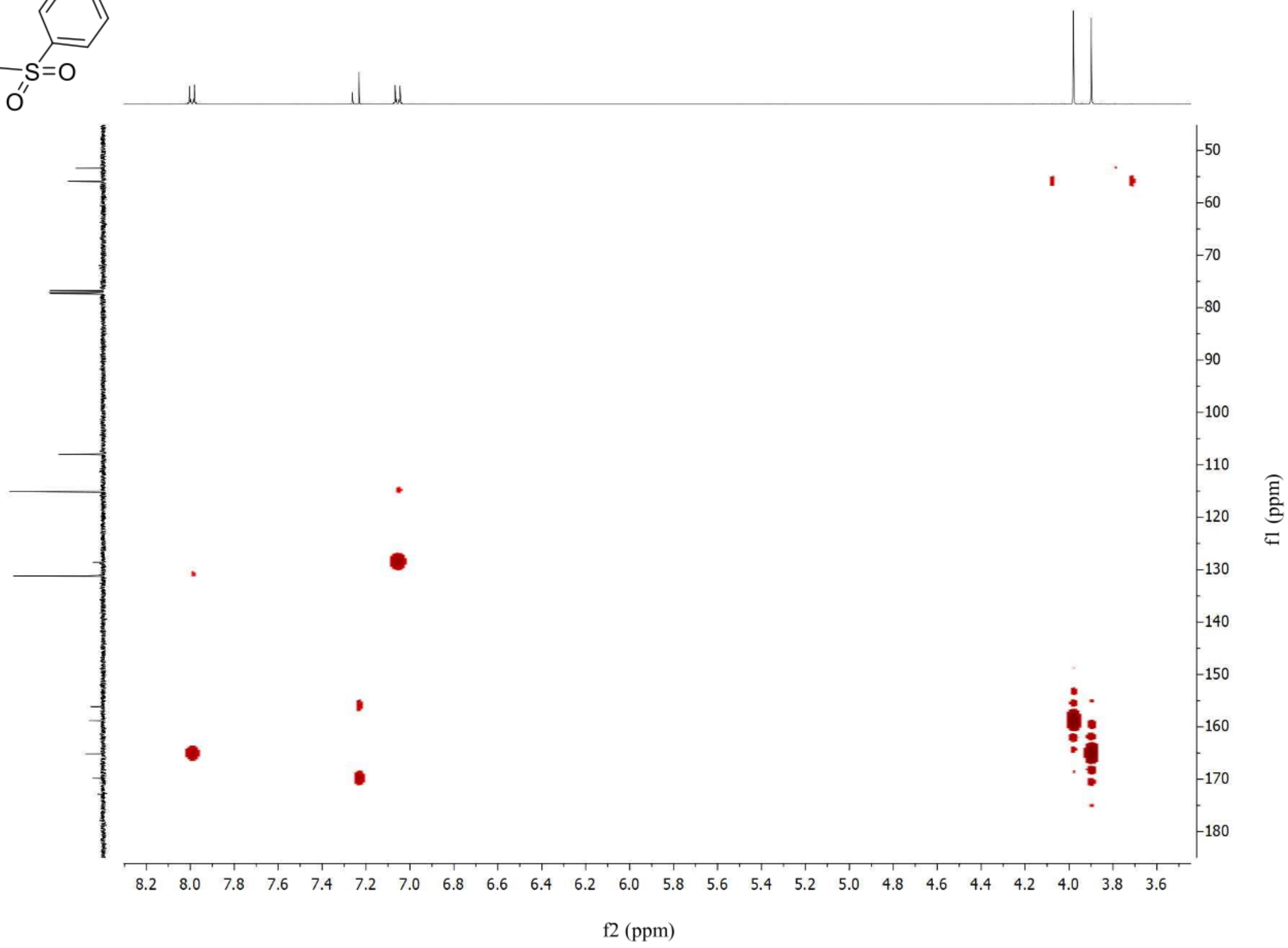

Methyl 5-(phenylsulfinyl)isoxazole-3-carboxylate **4a** (^{1}H NMR)

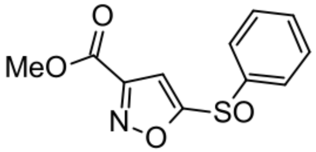

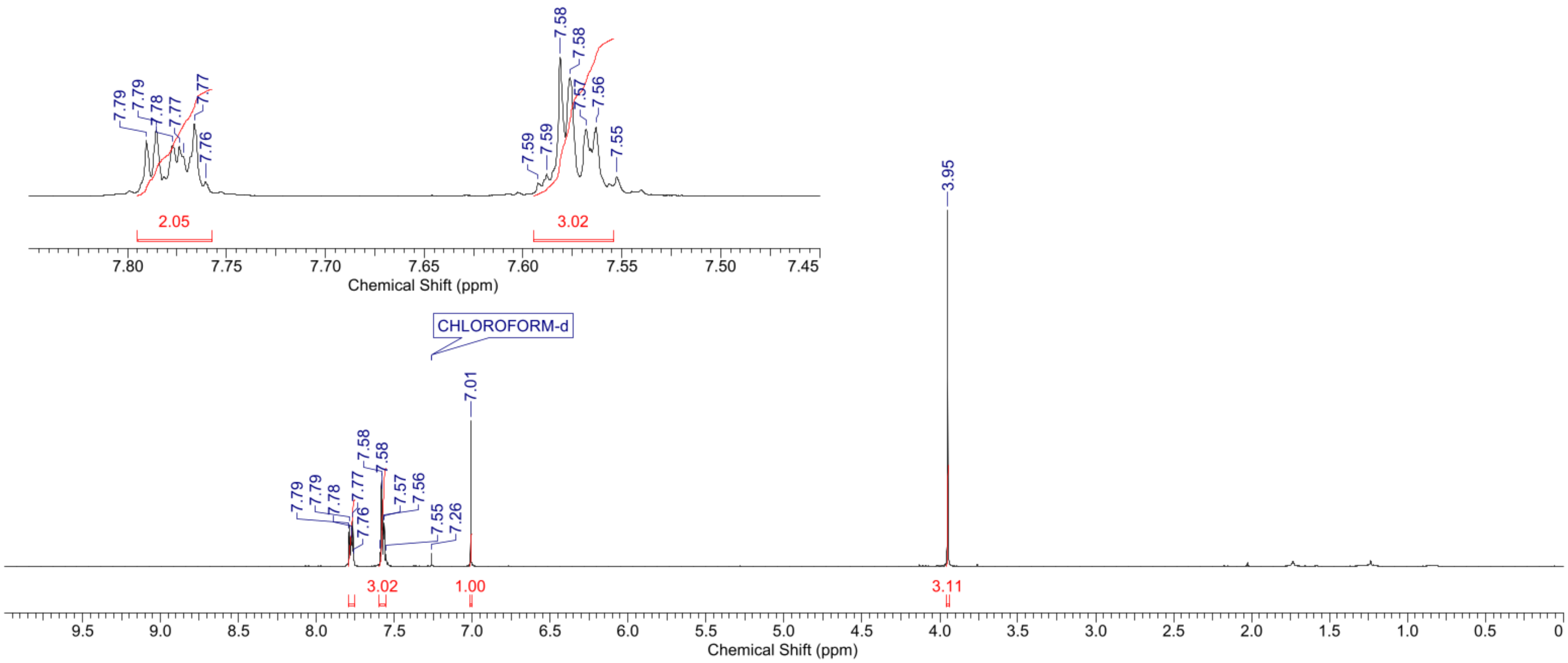

Methyl 5-(phenylsulfinyl)isoxazole-3-carboxylate **4a** (^{13}C NMR)

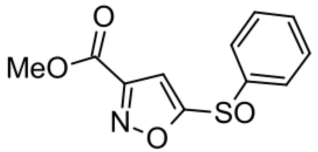

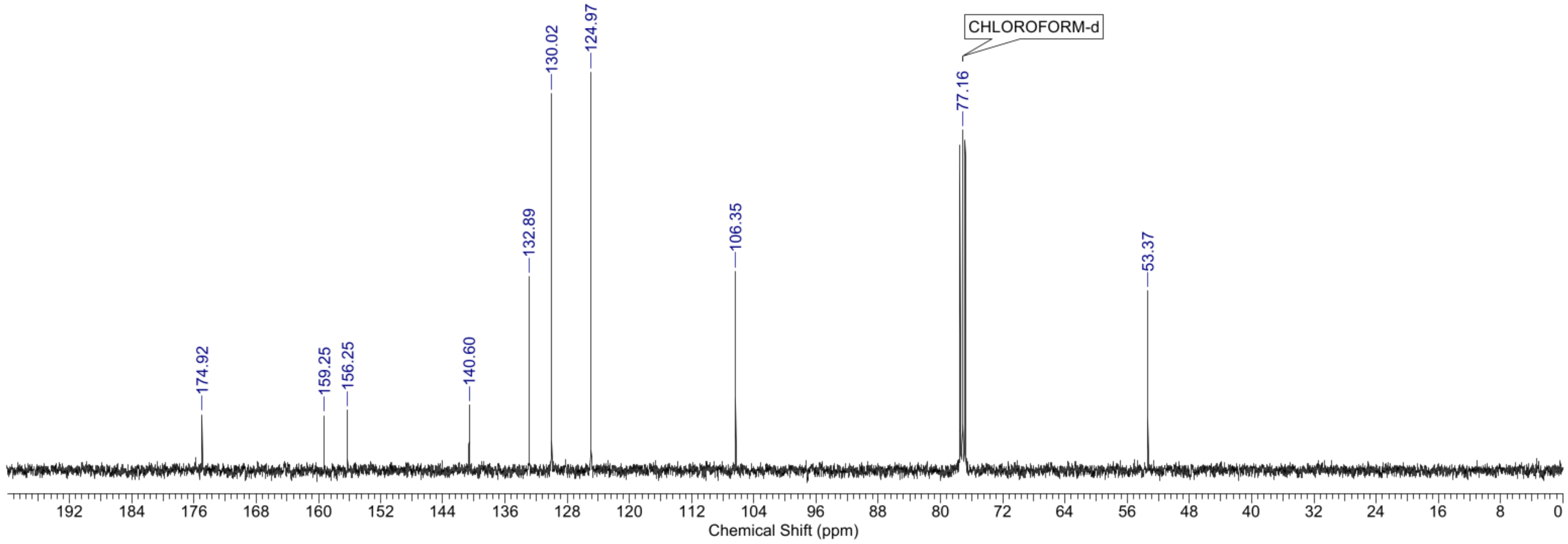

3-nitro-5-(phenylsulfinyl)isoxazole **4b** (^{1}H NMR)

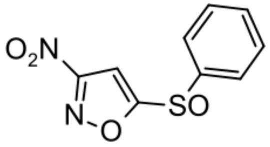

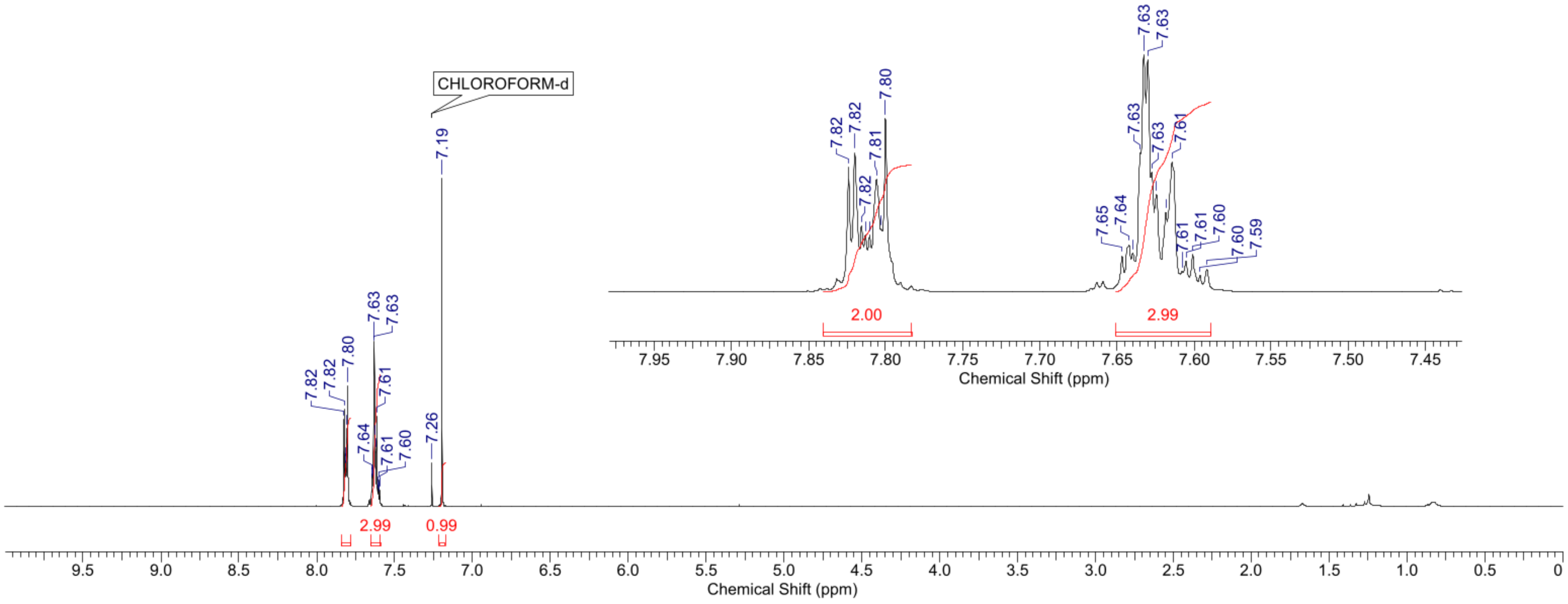

3-nitro-5-(phenylsulfinyl)isoxazole **4b** (^{13}C NMR)

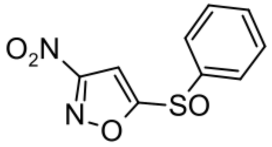

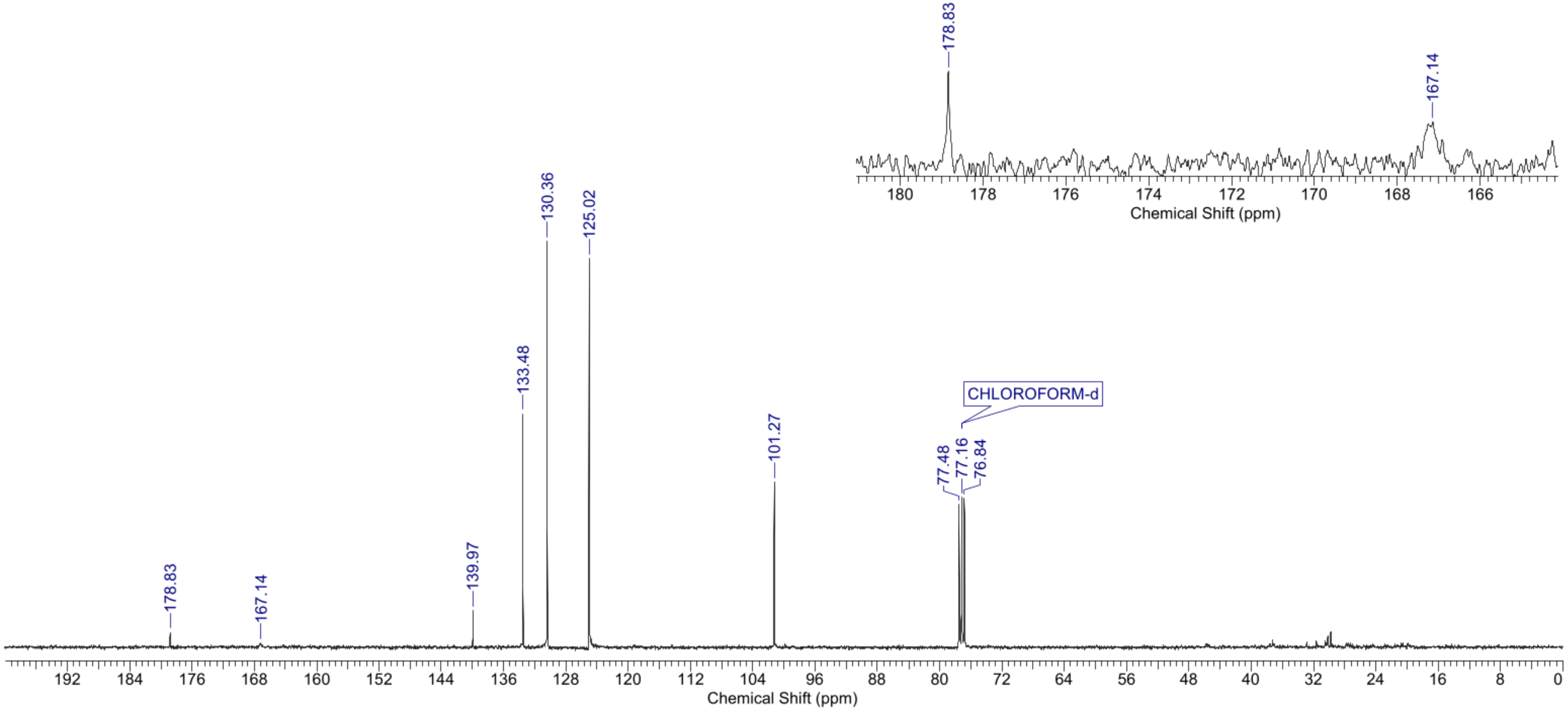

3-nitro-5-(phenylsulfinyl)isoxazole **4b** (HSQC)

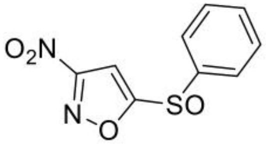

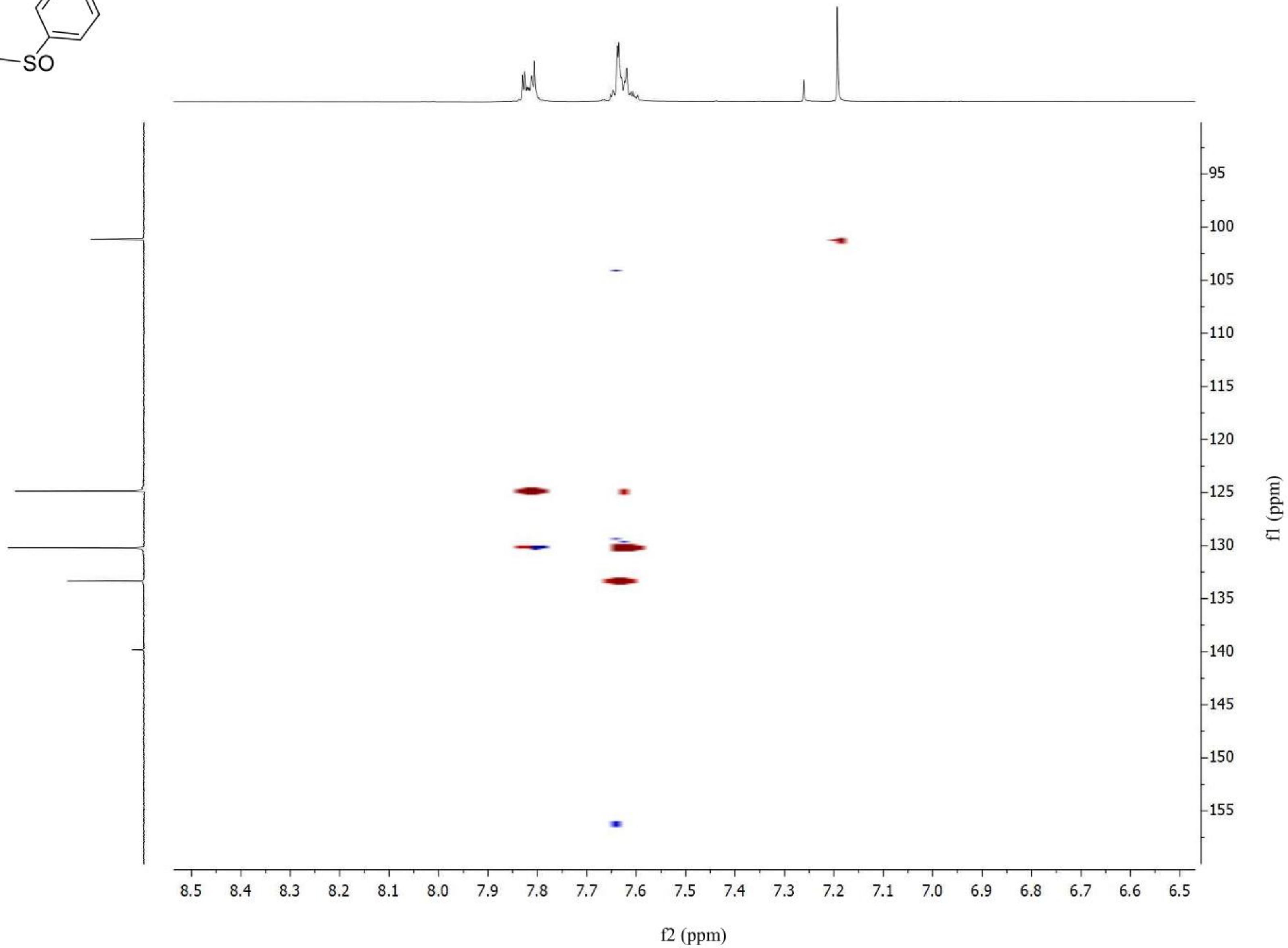

3-nitro-5-(phenylsulfinyl)isoxazole **4b** (HMBC)

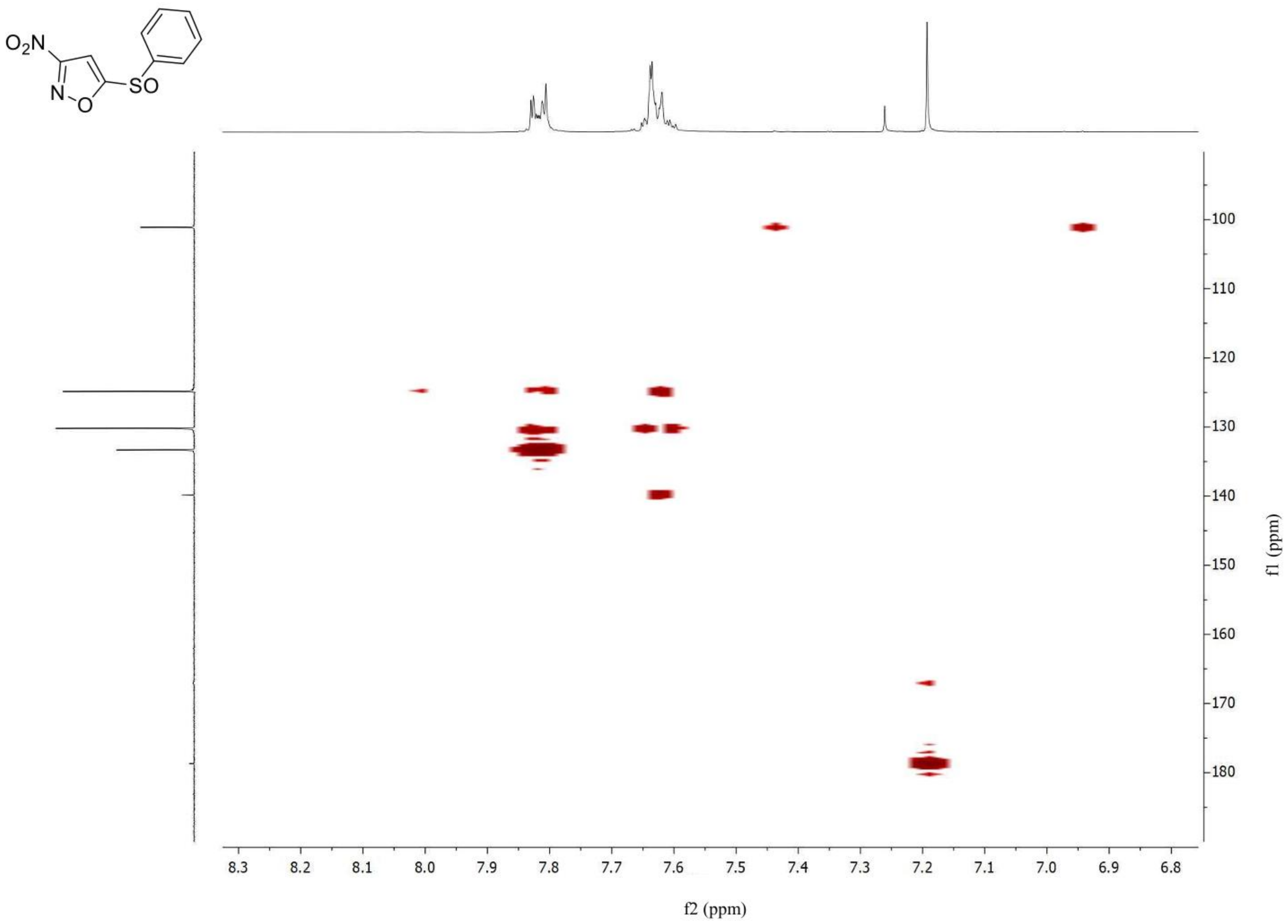

Supplement: File 1 — General synthetic and biological procedures, characterization data and copies of 1H, 13C{1H}, 19F, 31P, 1H-13C HSQC, 1H-13C HMBC NMR spectra, HRMS spectra and the results of the elemental analysis of all synthesized compounds. [file Beilstein_J_Org_Chem-22-592-s001.pdf]
